# ﻿Distribution and diversity of fishes and lampreys in Transylvania (Romania): a complete survey and suggestions for new protected areas

**DOI:** 10.3897/zookeys.1166.102854

**Published:** 2023-06-13

**Authors:** András Attila Nagy, Nándor Erős, István Imecs, Gábor Bóné, Attila Fülöp, Péter László Pap

**Affiliations:** 1 Evolutionary Ecology Group, 3B Centre for Systems Biology, Biodiversity and Bioresources, Hungarian Department of Biology and Ecology, Babeş-Bolyai University, Clinicilor street 5–7, RO–400006 Cluj-Napoca, Romania; 2 Milvus Group Bird and Nature Protection Association, Crinului street 22, RO–540343 Târgu Mureș, Romania; 3 Institute of Aquatic Ecology, Centre for Ecological Research, Bem square 18/C, H–4026 Debrecen, Hungary; 4 Department of Freshwater Fish Ecology, Hungarian University of Agricultural and Life Sciences, H–2100 Gödöllő, Hungary; 5 Department of Evolutionary Zoology and Human Biology, University of Debrecen, Egyetem tér 1, H–4032 Debrecen, Hungary; 6 Juhász-Nagy Pál Doctoral School, University of Debrecen, Egyetem square 1, H–4032 Debrecen, Hungary; 7 STAR-UBB Institute of Advanced Studies in Science and Technology, Babeş-Bolyai University, Mihail Kogălniceanu street 1, RO– 400084 Cluj-Napoca, Romania; 8 ELKH-DE Behavioural Ecology Research Group, Department of Evolutionary Zoology and Human Biology, University of Debrecen, Egyetem square 1, H–4032 Debrecen, Hungary

**Keywords:** Conservation, fish distribution, freshwater ichthyofauna, Natura 2000, non-native species

## Abstract

Freshwater fishes are in a serious state of decline across the world, making them one of the most threatened groups of vertebrates. The Danube River catchment area in Europe holds the richest freshwater fish community, but our knowledge of the current distribution of these species is limited. Transylvania, the largest region of Romania, is one of the important tributaries of the Danube, from where 77 fish and two lamprey species were recorded until now. Despite this large diversity of freshwater fishes, there is a lack of systematic survey of the fish fauna in this region for the past 50 years. In this study, we present data on the occurrence and distribution of fishes and lampreys collected in Transylvania from 2007 to 2022. This data covers 43% of Romania’s surface and includes all major rivers from Transylvania. 65 species of fish and three species of lampreys are recorded, and an additional nine fish species are also reported based on information from competent people. Of the 77 fish and lamprey species recorded 19 (24.7%) are non-native, although their relative abundance was low (5.1%) compared to other similar regions in Europe. The first records of *Eudontomyzonmariae*, *Neogobiusmelanostomus*, *Piaractusbrachypomus*, *Pygocentrusnattereri*, and *Salvelinusalpinus* in Transylvanian rivers are presented, as well as the first record of *Cobitiselongata* outside the Nera River basin (from the Caraș River) and the detection of three new populations of the vulnerable *Umbrakrameri*. Data on changes in distribution that have occurred since the last comprehensive survey 50 years ago are also provided and the importance of our results in conservation planning are discussed, including the designation of new protected areas for freshwater bodies and the compilation of the Romanian Red List of fishes.

## ﻿Introduction

Freshwater fishes make up 50% of all fish species (Fricke et al. 2023) and approximatively 25% of all vertebrates (Pough et al. 1999). They make an important contribution to global biodiversity (Dudgeon et al. 2006; Tedesco et al. 2013), and by providing important ecosystem services, they are essential to the maintenance and functioning of freshwater ecosystems (Miqueleiz et al. 2020). Freshwater fish populations are in serious decline worldwide (Moyle and Leidy 1992; Burkhardt-Holm et al. 2005; Xenopoulos et al. 2005; Freyhof and Brooks 2011), making them one of the most threatened groups of vertebrates (Reid et al. 2013). Nearly half (41.2%) of the European native freshwater fish species assessed by the IUCN Red List are considered threatened (Costa et al. 2021), and similar results have been found by other studies (39% according to Darwall and Freyhof 2015). Consequently, the European Union aims to increase the protected areas of its terrestrial surface, including freshwater bodies, to 30% by 2030, with one third of this area being strictly protected (Miu et al. 2020).

Identification of biodiversity hotspots, areas with high concentrations of individuals, such as those used for reproduction, as well as of endemic species and the invasive species threatening native communities, is critical for actions aiming to reduce biodiversity loss (Myers 1988; Gaston et al. 2002). Therefore, to reach conservation goals, detailed information on the distribution of species is crucial, along with knowledge on their ecology, biogeography, and phylogeography (Margules et al. 2002; Cogălniceanu et al. 2013). For fishes, the conservation status of many species is complicated by frequent changes in nomenclature and the widespread acceptance of the Phylogenetic Species Concept (Economou et al. 2007; Koutsikos et al. 2012). Lastly, among the 25 IUCN threat types affecting European freshwater fish, “Invasive Non-Native/Alien Species/Diseases” is the third largest threat, impacting 33.6% of the species (Costa et al. 2021). Therefore, recent data on the distribution of both native and non-native species is important to have.

The last comprehensive survey of the fish fauna of freshwater habitats in Romania, including Transylvania, dates back to 1964 (Bănărescu 1964). In the recent decades, several surveys have been carried out on major rivers in Transylvania, including the Mureș (Nalbant 1995), Criș (Bănărescu et al. 1997), Someș (Bănărescu et al. 1999), Olt (Bănăduc 1999), and Timiș (Bănăduc et al. 2013). However, surveys of smaller rivers, such as Tur, Bega, Crasna, Barcău, Ier, Lăpuș, Arieș, among others, are still scarce (Harka et al. 1998; Harka and Bănărescu 1999; Wilhelm et al. 2001, 2002; Wilhelm 2007, 2008b). The fish fauna of some protected areas in Transylvania has been recently surveyed (Pricope et al. 2009; Imecs and Nagy 2012; Imecs et al. 2014; Telcean et al. 2014; Năstase and Oţel 2016; Năstase and Tošić 2016; Nagy et al. 2019; Nagy and Imecs 2020), but these areas only account for a small amount of the total protected areas in Transylvania. Few other studies on smaller rivers and lakes have been published (Battes and Pricope 2006; Cocan et al. 2020; Lațiu et al. 2022). Given the current gaps in the distribution, abundance, species identity and occurrence of native and non-native species in the Transylvanian rivers, a comprehensive and large-scale survey is necessary to support the conservation efforts of the highly diverse freshwater fish species in the region. Therefore, our objective in this study is to provide updated information on the distribution and abundance of all freshwater fish and lamprey species in Transylvanian rivers, considering the recent taxonomic nomenclature.

## ﻿Materials and methods

### ﻿Study area

**Figure 1.**
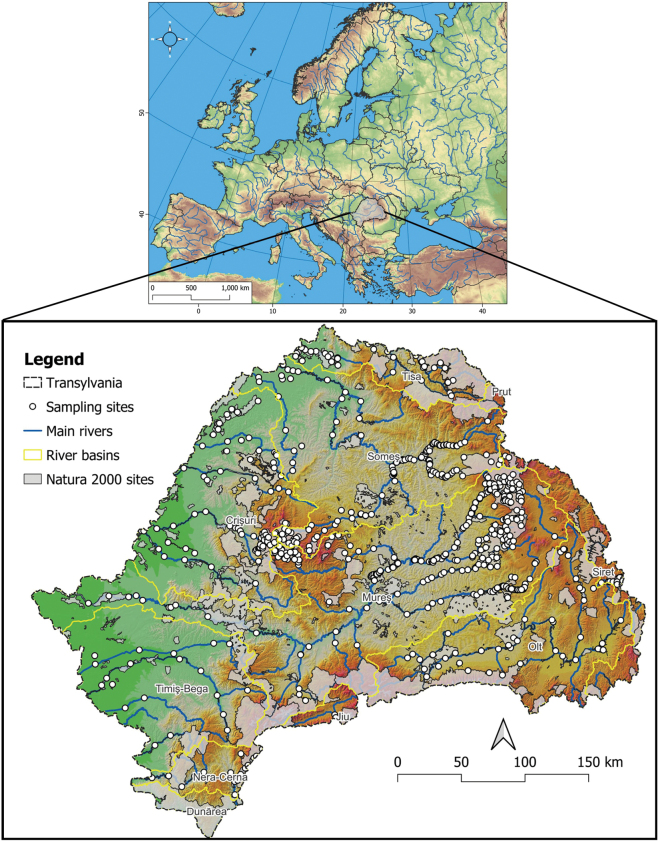
Position of Transylvania, Romania, in Europe, showcasing the main rivers, river basins, Natura 2000 sites (SCIs – Site of Community Interest), and the sampling sites surveyed in the study.

Transylvania is the largest region of Romania, covering an area of 102,226 km^2^ (43% of Romania). It is bordered by the Carpathian Mountains in the north, east, and south, and by the Pannonian Plain in the west (see Fig. 1). It encompasses three biogeographical regions: Alpine (29.7%), Continental (55.3%), and Pannonian (15.0%). The majority of the larger rivers in the region originate from the Carpathians and flow towards the west (Pannonian Plain). The Mureș is the longest river of Transylvania, stretching over 766 km (718 km in Romania) with a discharge/outflow of 165 m^3^/s at the Romanian-Hungarian border. Other large rivers include the Someș River (Someșul Mic and Someșul Mare form the united Someș), which has a length of 345 km in Romania (Transylvania) and a discharge of 118 m^3^/s, the Criș Rivers (Crișul Repede with 148 km, Crișul Negru with 144 km, and Crișul Alb with 238 km), Bega River (244 km), and the Timiș River (359 km). The lowest sampling site from our study is located at 78 m above sea level on the Timiș River, while the highest sampling site is situated at 1356 m above sea level on the Someșul Rece River.

In the past two centuries, river regulations have affected Transylvanian rivers, especially floodplains and marshes. The largest marsh, the Ecsed Moor, situated on the boundary between Romania and Hungary, was drained in the 19^th^ century. Another important lowland floodplain, the Ier River valley, was also drained. Most of this land has been converted into agricultural land. After accession to the European Union in 2007, agriculture intensified significantly, with monocultures taking priority over small parcels of land, which probably have an effect on the fish communities of the rivers. Although there are relatively few large cities in the region, the numerous villages may pose an important source of pollution. The five largest cities in Transylvania are Cluj Napoca (with 286,598 inhabitants), Timișoara (250,849), Brașov (237,589), Oradea (183,105), and Arad (145,078) (2021 population census).

### ﻿Data collection

Data were collected between 31 March 2007 and 29 October 2022 from a total of 679 sampling sites, including all rivers and major tributaries in Transylvania (Fig. 1). Our survey was focused on rivers, still we occasionally sampled backwaters, ponds, and drainage channels with stagnant water to gather data on species that inhabit these waters and are threatened by habitat loss. Standing waters represent 6% of the total sampling sites. Fishponds and artificial lakes were not surveyed at all. We chose the location of sampling sites to ensure relatively uniform coverage of each river. Although we invested higher sampling efforts in some areas, we attempted to achieve representative coverage of all rivers (Fig. 1). Fishing was carried out with a 12V battery-powered electrofishing device (Samus 725 or Samus 1000), and data collection was performed by wading (Sály et al. 2009). This method consists of moving slowly upstream in the shallow waters and fishing on one side of the stream in a single pass. Due to current legislative constraints, we were unable to fish from inflatable boats, which is preferable on larger rivers and lakes. Therefore, the fishing method used in our study may underestimate the presence and abundance of species inhabiting deep and/or large (stagnant) water bodies. The length of the sampling sites was set to 150 m in small and medium-sized rivers and 200–300 m in larger rivers, although occasionally, the length of the sampling sites had to be adjusted according to the local field conditions (e.g., shortened if the site was inaccessible). After capture, identification, and taking occasionally morphometric measurements, all individuals were released in good condition. The raw survey data are stored in the OpenFishMaps database, an open-source database available at https://openfishmaps.ro/, and are available in Suppl. material 1.

We compiled the distribution maps of species using the data from our survey and information provided by anglers, angling associations, fish stocking projects, or the Facebook page “Ichthyology of Romania” (https://www.facebook.com/groups/ichthyologyofromania). From the sources other than our own capture data we only used data that were supported by documentary photographs so that the species could be accurately identified. Information obtained from these sources and additional personal occurrence data for four species (*Cottusgobio*, *Eudontomyzonmariae*, *Sabanejewiaromanica*, *Umbrakrameri*) is not included in the raw data of distribution (Suppl. material 1) but is indicated separately in the distribution maps (Suppl. material 2). We excluded all hybrids from the dataset, particularly the *Barbusbarbus* × *Barbusbiharicus*, *Romanogobiouranoscopus* × *Gobiogobio* sensu lato, and the *Barbuspetenyi* × *Barbusbarbus*. The two *Sabanejewia* species previously belonging to *S.aurata* (*S.balcanica* and *S.bulgarica*) were treated as *Sabanejewia* sp. because the identification of these species in some rivers was uncertain.

### ﻿Spatial analyses

The survey data stored in the OpenFishMaps database were exported to the R statistical environment (v. 4.2.2; R Core Team 2022) using ESRI shape files. As the database contains data from all regions of Romania, we first selected only the data points within the boundary of Transylvania, then we applied descriptive statistics. For the graphical visualization of the data, to better showcase the distributional patterns of species and different groups of species within Transylvania, we assigned to all sampling sites the cell codes of the overlapping 50 × 50 km ETRS grid, and counted the total number of species, number of native species, number of non-native species, and the number of Natura 2000 species (found in Annex II of the EU’s Habitat Directive) for each grid cell. Detailed distribution maps for each species separately are provided in Suppl. material 2. The filtering of the spatial data was performed with the “sf” R package (Pebesma 2018), while data operations were performed with the “dplyr” R package (Wickham et al. 2022). Data visualization was performed using “tmap” R package (Tennekes 2018) and Quantum GIS (version 3.22; QGIS Development Team 2022).

## ﻿Results

Between 2007 and 2022 we have identified 129,212 individuals belonging to a total of 68 species (65 fish and 3 lamprey species) (Table 1; Suppl. material 1). In addition, the presence of nine other species (*Acipenserruthenus*, *Ballerusballerus*, *Coregonus* sp., *Gymnocephalusschraetser*, *Neogobiusmelanostomus*, *Piaractusbrachypomus*, *Pygocentrusnattereri*, *Salvelinusalpinus* and *Sandervolgensis*) was confirmed based on information from other verified sources (anglers, angling associations, fish stocking projects, or the Facebook page “Ichthyology of Romania”). Of the 77 identified species (74 fish and 3 lamprey), 19 are non-native. 21 fish and all three lamprey species are protected under the Natura 2000 legislation (Table 1).

**Table 1.** The complete checklist of freshwater fish and lamprey species of Transylvania (Romania). The taxonomy follows the FishBase online database (Froese and Pauly 2023) with slight modifications.

No.	Scientific name	Recorded until 1969 (Bănărescu 1964, 1969)	New species recorded between 1964 and 2022	Present study	Origin	Natura 2000 protection	Observation
**Petromyzontidae**							
1	*Eudontomyzondanfordi* Regan, 1911	x		x	native	yes	
2	*Eudontomyzonmariae* (Berg, 1931)			x	native	yes	
3	*Eudontomyzonvladykovi* Oliva & Zanandrea, 1959	x		x	native	yes	
**Acipenseridae**							
4	*Acipensergueldenstaedtii* Brandt & Ratzeburg, 1833	x			native	no	
5	*Acipenserruthenus* Linnaeus, 1758	x		x	native	no	
**Anguillidae**							
6	*Anguillaanguilla* (Linnaeus, 1758)	x			native	no	
**Cobitidae**							
7	*Cobitiselongata* Heckel & Kner, 1858	x		x	native	yes	
8	*Cobitiselongatoides* Băcescu & Maier, 1969	x		x	native	yes	
9	*Misgurnusfossilis* (Linnaeus, 1758)	x		x	native	yes	
10	*Sabanejewia* sp. (incuding *S.balcanica* (Karaman, 1922) and *S.bulgarica* (Drensky, 1928))	x Bănărescu (1964) treated these two spp as ssp: *Sabanejewiaauratabalcanica and S.a.bulgarica*.		x	native	yes	
11	*Sabanejewiaromanica* (Băcescu, 1943)	x		x	native	no	
**Nemacheilidae**							
12	*Barbatulabarbatula* (Linnaeus, 1758)	x		x	native	no	
**Cyprinidae**							
13	*Barbusbarbus* (Linnaeus, 1758)	x		x	native	no	
14	*Barbusbalcanicus* Kotlík, Tsigenopoulos, Ráb & Berrebi, 2002	x All species were treated together as *Barbusmeridionalispetenyi* by Bănărescu (1964).	x (Kotlík et al. 2002)	x	native	yes	
15	*Barbusbiharicus* Antal, László & Kotlík, 2016		x (Antal et al. 2016)	x	native	yes	
16	*Barbuscarpathicus* Kotlík, Tsigenopoulos, Ráb & Berrebi, 2002		x (Kotlík et al. 2002)	x	native	yes	
17	*Barbuspetenyi* Heckel, 1852			x	native	yes	
18	*Carassiuscarassius* (Linnaeus, 1758)	x		x	native	no	
19	*Carassiusgibelio* (Bloch, 1782)	x		x	non-native	no	
20	*Cyprinuscarpio* Linnaeus, 1758	x		x	native	no	
**Xenocyprididae**							
21	*Ctenopharyngodonidella* (Valenciennes, 1844)		x (Bănărescu 1981)	x	non-native	no	
22	*Hypophthalmichthysmolitrix* (Valenciennes, 1844)		x (Bănărescu 1981)	x	non-native	no	
23	*Hypophthalmichthysnobilis* (Richardson, 1845)		x (Bănărescu 1981)	x	non-native	no	
**Tincidae**							
24	*Tincatinca* (Linnaeus, 1758)	x		x	native	no	
**Acheilognathidae**							
25	*Rhodeusamarus* (Bloch, 1782)	x		x	native	yes	
**Gobionidae**							
26	*Gobiogobio* sensu lato (Linnaeus, 1758)	x		x	native	no	The taxonomic position of stream dwelling gudgeons is still not clearly detailed (see Takács et al. 2021). Nowak et al. (2008) and Takács (2018) recommended the use of this taxonomic concept.
27	*Gobioobtusirostris* Valenciennes, 1842	The species was treated as a subspecies of *Gobiogobio* by Bănărescu (1964).	x (Takács et al. 2021)	x	native	no	
28	*Pseudorasboraparva* (Temminck & Schlegel, 1846)		x (Bănărescu 1981)	x	non-native	no	
29	*Romanogobiokesslerii* (Dybowski, 1862)	x		x	native	yes	
30	*Romanogobiouranoscopus* (Agassiz, 1828)	x		x	native	yes	
31	*Romanogobiovladykovi* (Fang, 1943)	x		x	native	yes	
**Leuciscidae**							
32	*Abramisbrama* (Linnaeus, 1758)	x		x	native	no	
33	*Alburnoidesbipunctatus* (Bloch, 1782)	x		x	native	no	
34	*Alburnusalburnus* (Linnaeus, 1758)	x		x	native	no	
35	*Ballerusballerus* (Linnaeus, 1758)	x		x	native	no	
36	*Ballerussapa* (Pallas, 1814)	x		x	native	no	
37	*Bliccabjoerkna* (Linnaeus, 1758)	x		x	native	no	
38	*Chondrostomanasus* (Linnaeus, 1758)	x		x	native	no	
39	*Leucaspiusdelineatus* (Heckel, 1843)	x		x	native	no	
40	*Leuciscusaspius* (Linnaeus, 1758)	x		x	native	yes	
41	*Leuciscusidus* (Linnaeus, 1758)	x		x	native	no	
42	*Leuciscusleuciscus* (Linnaeus, 1758)	x		x	native	no	
43	*Pelecuscultratus* (Linnaeus, 1758)	x			native	yes	
44	*Phoxinusphoxinus* (Linnaeus, 1758)	x		x	native	no	
45	*Rutilusrutilus* (Linnaeus, 1758)	x		x	native	no	
46	*Rutilusvirgo* (Heckel, 1852)	x			native	yes	
47	*Scardiniuserythrophthalmus* (Linnaeus, 1758)	x		x	native	no	
48	*Squaliuscephalus* (Linnaeus, 1758)	x		x	native	no	
49	*Telestessouffia* (Risso, 1827)	x		x	native	yes	
50	*Vimbavimba* (Linnaeus, 1758)	x		x	native	no	
**Serrasalmidae**							
51	*Piaractusbrachypomus* (Cuvier, 1818)			x	non-native	no	
52	*Pygocentrusnattereri* Kner, 1858			x	non-native	no	
**Siluridae**							
53	*Silurusglanis* Linnaeus, 1758	x		x	native	no	
**Ictaluridae**							
54	*Ameiurusmelas* (Rafinesque, 1820)		x (Wilhelm 1998)	x	non-native	no	
55	*Ameiurusnebulosus* (Leseur, 1819)	x		x	non-native	no	
**Esocidae**							
56	*Esoxlucius* Linnaeus, 1758	x		x	native	no	
**Umbridae**							
57	*Umbrakrameri* Walbaum, 1792		x (Bănărescu 1981)	x	native	yes	
**Salmonidae**							
58	*Coregonusalbula* (Linnaeus, 1758)	x		x *Coregonus*. sp.	non-native	no	
59	*Coregonuslavaretus* (Linnaeus, 1758)	x			non-native	no	
60	*Huchohucho* (Linnaeus, 1758)	x		x	native	yes	
61	*Oncorhynchusmykiss* (Walbaum, 1792)	x		x	non-native	no	
62	*Salmotrutta* Linnaeus, 1758	x		x	native	no	
63	*Salvelinusalpinus* (Linnaeus, 1758)			x	non-native	no	
64	*Salvelinusfontinalis* (Mitchill, 1814)	x		x	non-native	no	
65	*Thymallusthymallus* (Linnaeus, 1758)	x		x	native	no	
**Lotidae**							
66	*Lotalota* (Linnaeus, 1758)	x		x	native	no	
**Odontobutidae**							
67	*Perccottusglenii* Dybowski, 1877		x (Covaciu-Marcov et al. 2011)	x	non-native	no	
**Gobiidae**							
68	*Babkagymnotrachelus* (Kessler, 1857)		x (Cocan et al. 2016)	x	non-native	no	
69	*Neogobiusfluviatilis* (Pallas, 1814)		x (Cocan et al. 2014)	x	non-native	no	
70	*Neogobiusmelanostomus* (Pallas, 1814)			x	non-native	no	
71	*Proterorhinussemilunaris* (Heckel, 1837)	x		x	non-native	no	
**Poeciliidae**							
72	*Gambusiaaffinis* (Baird & Girard, 1853)	x			non-native	no	
73	*Poeciliareticulata* Peters, 1859		x (Bănărescu et al. 1997)		non-native	no	
**Centrarhidae**							
74	*Lepomisgibbosus* (Linnaeus, 1758)	x		x	non-native	no	
**Percidae**							
75	*Gymnocephalusbaloni* Holčic & Hensel, 1974		x (Bănărescu 1981)	x	native	yes	
76	*Gymnocephaluscernua* (Linnaeus, 1758)	x		x	native	no	
77	*Gymnocephalusschraetser* (Linnaeus, 1758)	x		x	native	yes	
78	*Percafluviatilis* Linnaeus, 1758	x		x	native	no	
79	*Sanderlucioperca* (Linnaeus, 1758)	x		x	native	no	
80	*Sandervolgensis* (Gmelin, 1789)		x (Telcean and Bănărescu 2002)	x	native	no	
81	*Zingelstreber* (Siebold, 1863)	x		x	native	yes	
82	*Zingelzingel* (Linnaeus, 1766)	x		x	native	yes	
**Cottidae**							
83	*Cottusgobio* Linnaeus, 1758	x		x	native	yes	
84	*Cottuspoecilopus* Heckel, 1837	x		x	native	no	

Note: *Petroleuciscusborysthenicus* (Kessler, 1859) was reported from Mureș River basin by Nalbant (1995) but later the author admitted that it was a misidentification (pers. comm. Vasile Oțel, 27 February 2023).

Species with the highest number of occurrences in our sampling sites were the *Squaliuscephalus* (present in 56.6% of the sampling sites), *Alburnoidesbipunctatus* (51%), *Gobiogobio* sensu lato (39.3%), *Sabanejewia* sp. (including *S.balcanica* and *S.bulgarica*) (37.8%) and the *Rhodeusamarus* (37.7%). Species with the highest number of individuals captured were the *Alburnoidesbipunctatus* (16.8% of all individuals), *Squaliuscephalus* (10.5%), *Barbuspetenyi* (9.8%), *Rhodeusamarus* (7.8%) and the *Alburnusalburnus* (5.6%). The following species had the lowest occurrence: *Ameiurusnebulosus* (captured at one site), *Eudontomyzonvladykovi* (1), *Hypophthalmichthysnobilis* (1), *Babkagymnotrachelus* (2), *Eudontomyzonmariae* (2), *Gymnocephalusbaloni* (2), *Hypophthalmichthysmolitrix* (2), *Leuciscusidus* (2), *Ctenopharyngodonidella* (3) and *Gymnocephaluscernua* (3), while the 10 least abundant species were the *Ameiurusnebulosus* (one individual), *Eudontomyzonmariae* (4), *Gymnocephalusbaloni* (4), *Perccottusglenii* (5), *Hypophthalmichthysnobilis* (5), *Babkagymnotrachelus* (7), *Leuciscusidus* (8), *Huchohucho* (10), *Gymnocephaluscernua* (13) and *Lotalota* (14).

Four fish species caught by fishermen are reported for the first time from Transylvanian natural waters: *Neogobiusmelanostomus*, *Pygocentrusnattereri*, *Salvelinusalpinus* (all three species caught in 2022) and *Piaractusbrachypomus* (caught in 2020 and 2021). *Eudontomyzonmariae* is recorded for the first time from Transylvanian waters, and *Cobitiselongata* is recorded for the first time in Transylvania (in the Caraș River), out of its exclusive occurence in the Nera River basin. We found three new populations of the vulnerable *Umbrakrameri*.

**Figure 2.**
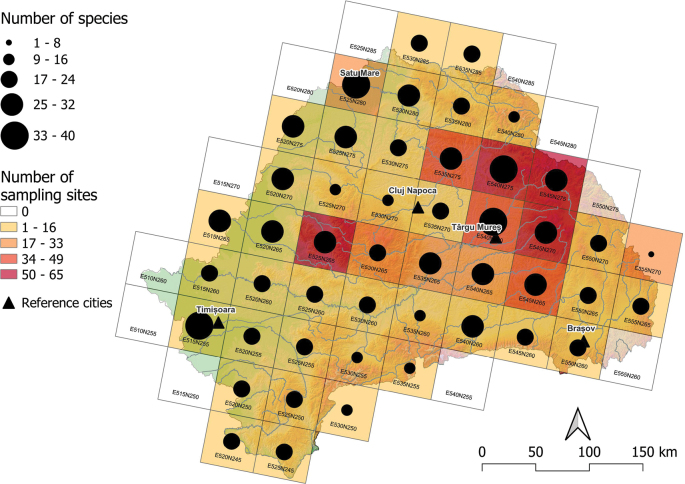
Distribution of total fish and lamprey species, surveyed in 50 × 50 km ETRS grids in Transylvania, Romania. The color of the ETRS grid cells indicates the number of sampling sites, and the size of the dots is proportional with the number of species.

The number of fish and lamprey species was the highest (33–40 species; Fig. 2) in the following 50×50 km ETRS grid cells: E525N280 (in the lower Someș basin, 38 species), E540N275 (in the upper Mureș basin, 36) and E515N255 (in the lower Timiș-Bega basin, 36). In contrast, low species numbers (1–8) were observed in E555N270 (Olt and Trotuș River basin, 4 species), E535N255 (9), E530N250 (10) and E530N255 (11) grid cells (upper Jiu, upper Nera-Cerna River, and upper Strei from Mureș basin), albeit these grid cells fall on the boundary of Transylvania and are in mountainous areas (Fig. 2).

**Figure 3.**
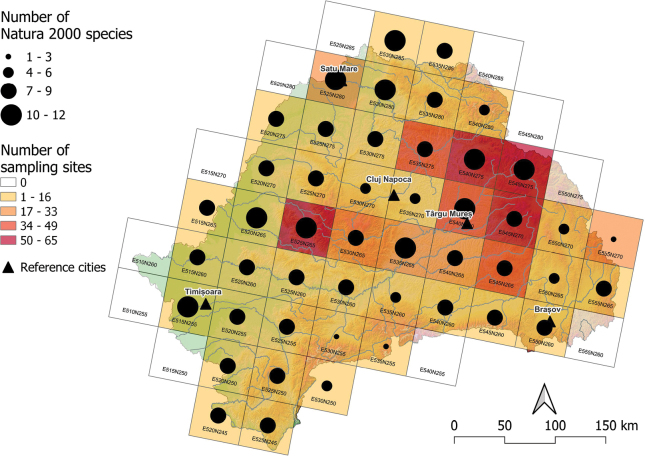
Distribution of Natura 2000 fish and lamprey species, surveyed in 50 × 50 km ETRS grids in Transylvania, Romania. The color of the ETRS grid cells indicates the number of sampling sites, and the size of the dots is proportional with the number of species.

The highest number of Natura 2000 species (10–12 Natura 2000 species) were located mainly in lowland areas, but not exclusively: E515N255 (in the lower Timiș-Bega River basin, 12 species), E540N275 (in the upper Mureș River basin, 12 spp), E525N280 (in the lower Someș River basin, 12 spp), E530N285 (Tisa River basin, 11 spp), E530N280 (in the lower Someș River basin, 11 spp), E540N270 (in the upper Mureș River basin, 11 spp), E520N265, E525N265 (in the Crișuri River Basin, 10 spp), E535N265 (middle Mureș River basin, 10 spp) and E545N275 (upper Mureș River basin, 10), (Fig. 3), while the least Natura 2000 fish and lamprey species (1–3 spp) were found in mountain areas: E555N270 (Olt and Trotuș River basin, 1 species), E535N255 (upper Jiu River, 2) and E530N255 (upper Timiș and Mureș River basins, 3 spp).

**Figure 4.**
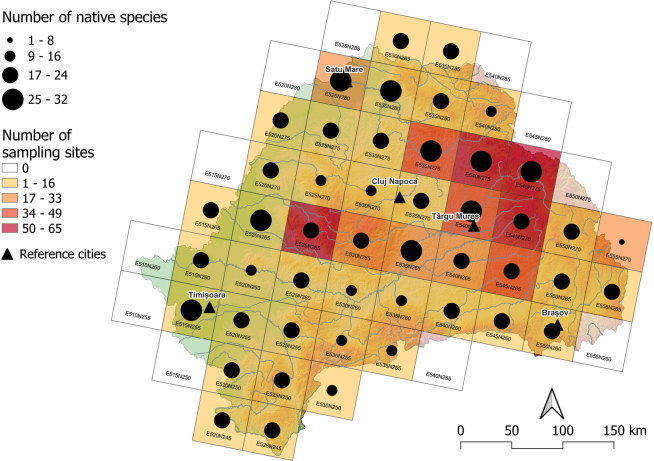
Distribution of native fish and lamprey species, surveyed in 50 × 50 km ETRS grids in Transylvania, Romania. The color of the ETRS grid cells indicates the number of sampling sites, and the size of the dots is proportional with the number of species.

The highest number of native species was found in lowland areas (E525N280 in the lower Someș River basin, 32 species) and in one grid cell from the hilly-mountainous area (E540N275 upper Mureș River basin, 31 spp) (Fig. 4). Lowest native species number was found in mountainous regions (E555N270, 4 spp, E535N255, 9 spp, and E530N250, 10 spp), albeit these grid cells fall on the boundary of Transylvania (Fig. 4).

**Figure 5.**
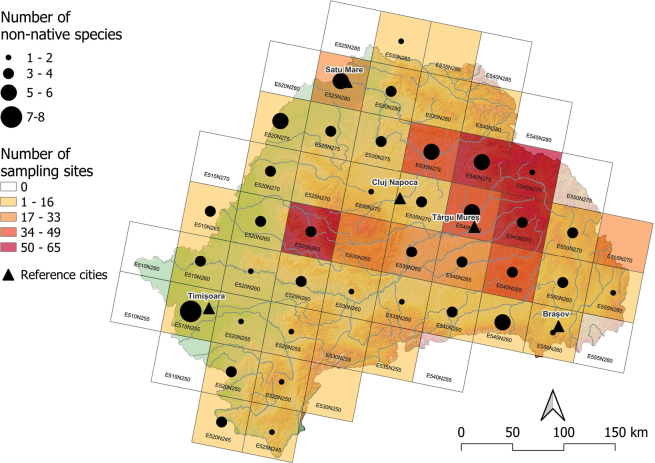
Distribution of non-native fish species, surveyed in 50 × 50 km ETRS grids in Transylvania, Romania. The color of the ETRS grid cells indicates the number of sampling sites, and the size of the dots is proportional with the number of species.

The abundance of non-native species was overall low (5.1%). The grid cell with the highest number of non-native species (7) was found in the lowland, in the lower Timiș-Bega River basins (E515N255), while the least invaded areas were found in mountainous areas (Fig. 5).

## ﻿Discussion

Out of our survey, a total of 77 species of fish and 2 species of lampreys have been recorded in Transylvanian rivers until now (Table 1). Our study reveals the occurrence of 74 fish and three lamprey species in the Transylvanian rivers and ponds. It is important to note that for comparison, we calculated the total species number recorded until now using the same nomenclature as used in this study. Out of the 60 fish and two lamprey species recorded by Bănărescu (1964, 1969) in his comprehensive survey carried out more than 50 years ago, we captured 55 fish and two lamprey species, suggesting a slight change in species pool in Transylvanian rivers (Table 1). This change is due to the absence of the following species that were recorded before: *Anguillaanguilla*, *Gambusiaaffinisholbrooki*, *Pelecuscultratus*, *Rutilusvirgo* and one of the two *Coregonus* species (*Coregonusalbula* or *Coregonuslavaretus*). These species were recorded sparsely by Bănărescu (1964), and unless there has been a significant increase in their occurrence and abundance between surveys, the likelihood of their recovery is low. Our survey provides an updated overview of the Transylvanian fish fauna. However, the descriptive nature of our study limits our ability to determine the causes of distribution changes. Nonetheless, this study serves as a strong background for future investigations and conservation planning (see below).

### ﻿Distribution and proportion of native and non-native species

Out of the 77 identified species recorded during our survey, 19 (24.7%) are introduced, while 24 (31.2%) species belong to Natura 2000 species. Of the total of 129,212 captures, 6,553 individuals (5.1%) belong to non-native species, and 46,497 individuals (36%) belong to Natura 2000 species. Overall, the abundance of non-native species in our study region can be considered relatively low. For example, in Hungarian waters, 28.8% of identified species and 18.3% of total captures are non-native (Takács et al. 2017). In various parts of the Mediterranean Basin 25% of fish species are non-native, and in the Iberian Peninsula, where the majority of Europe’s threatened fish populations can be found, the proportion of alien species reaches 50% (Leprieur et al. 2008; Clavero et al. 2010; Maceda-Veiga 2013). The distribution of both native and Natura 2000 species throughout Transylvania (Figs 3, 4) and the small number and low abundance of non-native species (Fig. 5) demonstrates that the ichthyofauna in the rivers of Transylvania is much closer to a natural state. Although anecdotal evidence suggests that stocking is still low, irresponsibly repopulating river sectors and lakes could potentially exert significant pressure on the river ecosystems from this region. For instance, *Salvelinusalpinus* is reported for the first time in Transylvanian natural waters. A few individuals were caught by fisherman in the Someșul Cald River upstream of the Fântânele reservoir, where it is presumed the species was introduced without authorization.

Comparing our data with those collected during the last comprehensive survey by Bănărescu (1964, 1969), major changes can be observed in the distribution of several species. We present the status of these species below.

*Carassiuscarassius* was prevalent in most floodplains in the past (i.e., before 1964) but has now vanished from most of its former habitats (Suppl. material 2: map S17). On the other hand, *Carassiusgibelio*, which was present in only a few habitats before 1964, has now expanded its distribution over the main rivers of Transylvania, excluding mountainous habitats (Suppl. material 2: map S18).

Our data indicates that the distribution range of *Zingelzingel* has decreased, as the species has disappeared from the Someșul Mare, Someșul Mic, Crișul Repede, Olt Rivers and the middle part of the Mureș River. We found viable populations of the species in the Someș, Crișul Negru, Crișul Alb, Mureș, and Timiș Rivers, and a very fragile population in the Bega River (Suppl. material 2: map S77). *Zingelstreber* has apparently disappeared from the Tur, Someșul Mic, Crasna, Barcău, Arieș, and Bega Rivers (Suppl. material 2: map S76). Our observation supports the findings of Brinker et al. (2018), who also noted a reduction in the historical range of the species in the upper Danube basin due to population fragmentation and habitat loss. It has to be mentioned though that our fishing method is not proper for evaluating populations of *Zingel* species (Szalóky et al. 2021), therefore the occurrence and abundance of these two *Zingel* species might be underestimated. Our results suggest that the species still maintains significant populations in the Mureș, Crișul Negru, Crișul Alb, and Nera Rivers. The last recorded sighting of the species in the Someș River was in 1964 (Bănărescu 1964), although several studies have been conducted on the ichthyofauna of the Someș River since then (Bănărescu et al. 1999; Năstase and Oţel 2016). Our survey found the species at five sampling sites along the Someș River and at one site along the Someșul Mare River. When studying the ichthyofauna of the Mureș River, Nalbant (1995) only found a few individuals of this species in the fishermen’s catch at the Gura Arieșului locality. Our survey found viable populations in the lower and upper-middle part of the Mureș River (the species was present at 24 sampling stations). The species is also present in the Timiș, Olt, and Târnava Rivers, but in much smaller numbers (Suppl. material 2: map S76).

We have observed a drastic reduction in the distribution of *Gymnocephalusschraetser*, as this species was not identified during our surveys, except a few records from other verified sources, although it was found in several rivers (Mureș, Crișul Repede, Crișul Negru, Crișul Alb Rivers) in the 1990s (Nalbant 1995; Bănărescu et al. 1999) and later in the Timiș River (Bănăduc et al. 2013). The species is still present in the Someș, Timiș, and Crișul Negru Rivers (single individuals were observed by local anglers; Suppl. material 2: map S35). Harka and Csipkés (2009) observed a similar drastic contraction of distribution in the Hungarian Bodrog River. Further surveys are needed to map the remaining populations of this species.

*Umbrakrameri* has disappeared from most of its known habitats, particularly from the Ier River valley and from the Carei Plane in north-west Transylvania. In a survey, the species was found only in two out of 13 sites where the species was formerly recorded (Wilhelm 2008a), but three new populations were discovered in the upper valley of the Ier River and one new, but fragile population in the Homorod River of the Crasna River basin. A new population was also found in the Timiș River basin by Covaciu-Marcov et al. (2018) and confirmed by the present study (Suppl. material 2: map S74). These new findings are likely not due to a range expansion of the species in recent decades, but rather because this region of the Romania is understudied.

The presence of *Romanogobiovladykovi* has increased as a result of human activities in the Tisa River basin (Telcean and Bănărescu 2002) and our study confirms former findings (Suppl. material 2: map S59). Another species, *Leuciscusleuciscus*, which had only one confirmed occurrence in the 1990s, has shown significant recovery and was detected in the catchment area of 7 rivers (Suppl. material 2: map S43).

*Huchohucho* has returned to the upper Mureș River basin due to stocking (Cengher 2007) after a few decades of absence, but the construction of the Răstolița dam may affect the survival of the species. We found viable population of the species in the Tisa and upper Mureș River basins. (Suppl. material 2: map S36).

Although the method used in our study was moderately suitable for assessing *Cyprinuscarpio* populations, our data indicates a massive decline of the species (Suppl. material 2: map S26), especially of the wild form. This change is possibly due to hybridization and river regulation (Freyhof 2010).

*Eudontomyzonmariae* is reported for the first time in Transylvanian waters and is present in the Olt River basin (Suppl. material 2: map S29). A new, large population of *Cobitiselongata* was found in the Caraș River, in addition to the previously known population in the Nera River basin (Suppl. material 2: map S20).

Three species, *Babkagymnotrachelus*, *Neogobiusfluviatilis*, and *Perccottusglenii*, have recently appeared in Transylvania (Covaciu-Marcov et al. 2011; Cocan et al. 2014, 2016). Our observations indicate that these species have expanded their range of distribution. *Babkagymnotrachelus* was found in the Timiș River basin (Suppl. material 2: map S7), *Neogobiusfluviatilis* was found in the Someș, Timiș, and Olt River basins (Suppl. material 2: map S46), and *Perccottusglenii* was found in the Tur, Crișul Repede, and Bega Rivers (Suppl. material 2: map S50). The rapid spread of *Perccottusglenii* in Europe and its impact on native fish fauna (Koščo et al. 2003; Reshetnikov 2003, 2013; Reshetnikov and Ficetola 2011; Horvatić et al. 2022) raise concerns, as it is already present in three Transylvanian rivers and its further spread is expected, posing a significant threat to the native fish fauna, especially to the vulnerable *Umbrakrameri* (Grabowska et al. 2019).

*Pseudorasboraparva*, a non-native species, was not present in Transylvania before 1964, but we found it in almost all river basins and at 19.9% of the sampling sites (Suppl. material 2: map S54). *Lepomisgibbosus* was present only in the western part of the region before 1964, but we found it in most of the river basins surveyed (Suppl. material 2: map S39). *Ameiurusnebulosus* was the dominant *Ameiurus* species in Transylvania’s waters until the 2000s (Wilhelm 2013), but it has now been almost completely replaced by *Ameiurusmelas* (Suppl. material 2: maps S5, S6). Only one specimen of *A.nebulosus* was identified in the Tur River. This replacement of *A.nebulosus* is similar to what has been observed in Hungarian waters (Takács et al. 2017) and confirms the observation of Jaćimović et al. (2019) regarding the invasive potential of this species.

### ﻿Conservation implications

Many of the Natura 2000 sites from Transylvania have been designated predominantly in mountainous areas to enhance the protection of Natura 2000 fish species, although only a few of these species occur there (as seen in E530N255 and E535N255). However, important river sectors in hilly and lowland areas, which have a high number of Natura 2000 fish and lamprey species, remain unprotected (such as parts of the Crișul Alb River from E520N265, the Bega and Bega Veche River from E515N255, the lower part of the Niraj River from E540N270, and important sectors of the Someș River and the middle and lower part of the Lăpuș River at E530N280). These hilly and lowland river sectors require protection as they are vulnerable to anthropogenic disturbances, particularly due to river regulation. These areas are home to most of the native and Natura 2000 species (Figs 3, 4). Furthermore, the presence of rare or endangered species alone is a sufficient reason to protect an entire aquatic habitat (such as the *Umbrakrameri* in the Timișul Mort River or Homorodul Vechi River). Therefore, we propose that in future designation of protected areas, not only the number of Natura 2000 species should be considered (Fig. 3), but also species occurrence maps. This is because, in some cases, the presence of rare or endangered species is a compelling argument for the establishment and designation of a protected area. Based on our survey, we suggest several river sectors for inclusion in the Natura 2000 network to ensure the protection of the most valuable and diverse river stretches (as listed in Table 2).

**Table 2.** River sections from Transylvania, Romania, proposed for protection and reasoning for designation. The ROSCI codes define the current Natura 2000 sites.

Proposed SCI	Reasoning for designation	Natura 2000 species for which protection is recommended	Description
Timișul Mort River	The largest *Umbrakrameri* population from Transylvania, according to our present knowledge	*Umbrakrameri*	From Pădureni to Macedonia (the whole sector of the Timișul Mort River and its floodplain that is not included currently in ROSCI0109 and ROSCI0348)
Homorodul Vechi River	The last and only known *Umbrakrameri* population from the Crasna River basin	*Umbrakrameri*	The whole Homorodul Vechi River and its floodplain (between Cionchești and confluence with the Crasna River)
Lăpuș River	One of the best preserved highland river sector with high fish diversity	*Romanogobiovladykovi*, *Romanogobiouranoscopus*, *Romanogobiokesslerii*, *Rhodeusamarus*, *Barbuscarpathicus*, *Cobitiselongatoides*, *Sabanejewiabalcanica*	From ROSCI0030 to the confluence with the Săsar River.
Someș River between Dej and Tămaia	High species diversity	*Romanogobiovladykovi*, *Romanogobiouranoscopus*, *Romanogobiokesslerii*, *Rhodeusamarus*, *Barbuscarpathicus*, *Cobitiselongatoides*, *Sabanejewiabalcanica*, *Zingelstreber*	From Dej to Tămaia, excluding the two short sections which are already Natura 2000 SCI (ROSCI0314 and ROSCI0435).
Upper basin of the Barcău River	High species diversity. The only known Transylvanian population of the *Salmotrutta* characterized with the phenotype of missing red spots.	*Eudontomyzondanfordi*, *Romanogobiokesslerii*, *Rhodeusamarus*, *Sabanejewiabalcanica*, *Cottusgobio*	Barcău River and its tributaries (Iaz, Valea Mare, Drighiu) from the ROSCI0322 to Marca locality
The middle sector of the Timiș River, between Prisaca and Lugoj	High species diversity	*Romanogobiouranoscopus*, *Romanogobiokesslerii*, *Rhodeusamarus*, *Barbuspetenyi*, *Sabanejewiabalcanica*, *Cobitiselongatoides*	Between Prisaca and Lugoj
Upper and middle sector of the Bega River	High species diversity. One of the few rivers from Transylvania where *Eudontomyzonvladykovi* still have stronghold populations	*Eudontomyzonvladykovi*, *Leuciscusaspius*, *Romanogobiovladykovi*, *Romanogobiouranoscopus*, *Romanogobiokesslerii*, *Rhodeusamarus*, *Barbuspetenyi*, *Sabanejewiabalcanica*, *Cobitiselongatoides*, *Misgurnusfossilis*, *Zingelzingel*	Between Luncanii de Jos and Timișoara
Bega Veche River	Natural lowland river habitat	*Misgurnusfossilis*, *Rhodeusamarus*	Between Săcălaz and the Romanian-Serbian national border
Upper Crișul Negru River	High species diversity	*Romanogobiovladykovi*, *Romanogobiouranoscopus*, *Romanogobiokesslerii*, *Rhodeusamarus*, *Barbusbiharicus*, *Sabanejewiabalcanica*, *Cobitiselongatoides*	Between Ștei and Uilacu de Beiuș
Crișul Alb River	High species diversity	*Romanogobiovladykovi*, *Romanogobiokesslerii*, *Rhodeusamarus*, *Leuciscusaspius*, *Sabanejewiabalcanica*, *Sabanejewiabulgarica*, *Cobitiselongatoides*, *Zingelstreber*, *Zingelzingel*	Between Ineu and Chișineu Criș.
Mureș River Between Aiud and Mintia	High species diversity	*Romanogobiovladykovi*, *Romanogobiouranoscopus*, *Romanogobiokesslerii*, *Rhodeusamarus*, *Barbuspetenyi*, *Sabanejewiabalcanica*, *Cobitiselongatoides*, *Zingelstreber*	Mureș River between Aiud and Mintia, except ROSCI0419
Niraj River	High species diversity	*Eudontomyzondanfordi*, *Romanogobiovladykovi*, *Romanogobiokesslerii*, *Rhodeusamarus*, *Barbuspetenyi*, *Sabanejewiabalcanica*, *Cobitiselongatoides*	From Eremitu to the confluence with the Mureș River

Considering that Romania does not currently have an officially adopted Red List for fish and lamprey species, our results can contribute to the creation of such a list. Some species, although not listed as Natura 2000 species, are of prime conservation concern due to drastic reductions in their distribution (e.g., *Carassiuscarassius*, *Leucaspiusdelineatus*, *Lotalota*, *Tincatinca*) or diminished abundance (*Thymallusthymallus*) in recent decades. It is crucial to assess their current conservation status to ensure their long-term survival. Many environmental impact assessments are hindered by a lack of up-to-date data on fish fauna, and often rely on assessments that are not appropriate for studying fish communities. Our data can provide valuable information for these conservation studies.

### ﻿Remarks on sampling

The species pool and distribution of some species in the study area is likely greater than what our survey shows due to several reasons. Firstly, we mainly sampled rivers and several species that were recorded in the past or are present in neighboring countries are expected to occur in the area, especially in stagnant or enclosed water bodies. Although we did sample a few backwaters to gather data on species that inhabit stagnant waters and are threatened by habitat loss (e.g., *Carassiuscarassius*, *Leucaspiusdelineatus*, *Umbrakrameri*), a comprehensive survey of these habitats was not conducted. Additionally, the fish fauna of thermal springs and lakes was excluded from the study, despite of some of these habitats are known to host exotic fish populations (Bănărescu et al. 1997). These waters are important sources and dispersal hotspots for some aquaristic cultivated fish species (Takács et al. 2015; Weiperth et al. 2015; Kordás and Juhász 2020) and may also serve as starting points for invasive species. Further sampling of these water bodies is likely to increase the number of introduced species in the Transylvanian fish fauna. Finally, the absence of certain species from our survey (e.g., *Pelecuscultratus*) and apparent gaps in the distribution of others, such as *Abramisbrama*, *Leuciscusaspius* or *Sanderlucioperca*, can be attributed to the limitations of our sampling method.

## ﻿Conclusions

Our study provides the most comprehensive and up-to-date data on the ichthyofauna of Transylvanian rivers in the last 50 years. Compared to the historically recorded 77 species of fish and two species of lampreys, we identified 74 fish and three lamprey species. The discovery of one lamprey and four new fish species for Transylvania (*Eudontomyzonmariae*, *Neogobiusmelanostomus*, *Piaractusbrachypomus*, *Pygocentrusnattereri* and *Salvelinusalpinus*) and new populations of several rare species (*Cobitiselongata*, *Sandervolgensis*, *Umbrakrameri*) highlights the need for further ichthyological research. There is also a need for a similar systematic assessment of the ichthyofauna of standing waters and ponds. Despite the negative impact of human activities on rivers in recent decades, these water bodies still hold a rich fish community that should be protected through designation of new protected areas as part of the Natura 2000 network. Urgent conservation measures are needed to ensure the long-term survival of non-Natura 2000 fish species, particularly those that have suffered significant range reductions. Anthropogenic pressure on fish populations is increasing, making necessary immediate conservation action in order to protect the diverse Transylvanian freshwater fish and lamprey populations.

## ﻿Additional information

### Author ORCIDs

Nándor Erős https://orcid.org/0000-0001-8921-482X

Attila Fülöp https://orcid.org/0000-0001-5337-336X

Péter László Pap https://orcid.org/0000-0002-3659-7684

### Data availability

All of the data that support the findings of this study are available in the main text or Supplementary Information.

## Supplementary materials

Supplementary material 1 Raw data of sampling sites, fish and lamprey species and their numbers Data type occurrences (excel file) https://binary.pensoft.net/file/863309This dataset is made available under the Open Database License (http://opendatacommons.org/licenses/odbl/1.0/). The Open Database License (ODbL) is a license agreement intended to allow users to freely share, modify, and use this Dataset while maintaining this same freedom for others, provided that the original source and author(s) are credited.András Attila Nagy, Nándor Erős, István Imecs, Gábor Bóné, Attila Fülöp, Péter László Pap

Supplementary material 2 Maps S1–S77 Data type occurrences (pdf file) Explanation note Distribution maps of fish and lamprey species in Transylvania, Romania. https://binary.pensoft.net/file/863310This dataset is made available under the Open Database License (http://opendatacommons.org/licenses/odbl/1.0/). The Open Database License (ODbL) is a license agreement intended to allow users to freely share, modify, and use this Dataset while maintaining this same freedom for others, provided that the original source and author(s) are credited.András Attila Nagy, Nándor Erős, István Imecs, Gábor Bóné, Attila Fülöp, Péter László Pap
