# Supplementary material for: ﻿Distribution and diversity of fishes and lampreys in Transylvania (Romania): a complete survey and suggestions for new protected areas
Source: Zookeys. 2023 Jun 13;1166:351–73. doi: 10.3897/zookeys.1166.102854 (PMC10848864; doi:10.3897/zookeys.1166.102854)
Supplement: Supplementary material 2 — Maps S1–S77 [file zookeys-1166-351_article-102854__-s002.pdf]

Supplementary material 2. Nagy et al. Distribution and diversity of fishes and lampreys in Transylvania (Romania): a complete survey and suggestions of new protected areas. ZooKeys xx
Maps S1–S77. Distribution maps of fish and lamprey species in Transylvania, Romania.

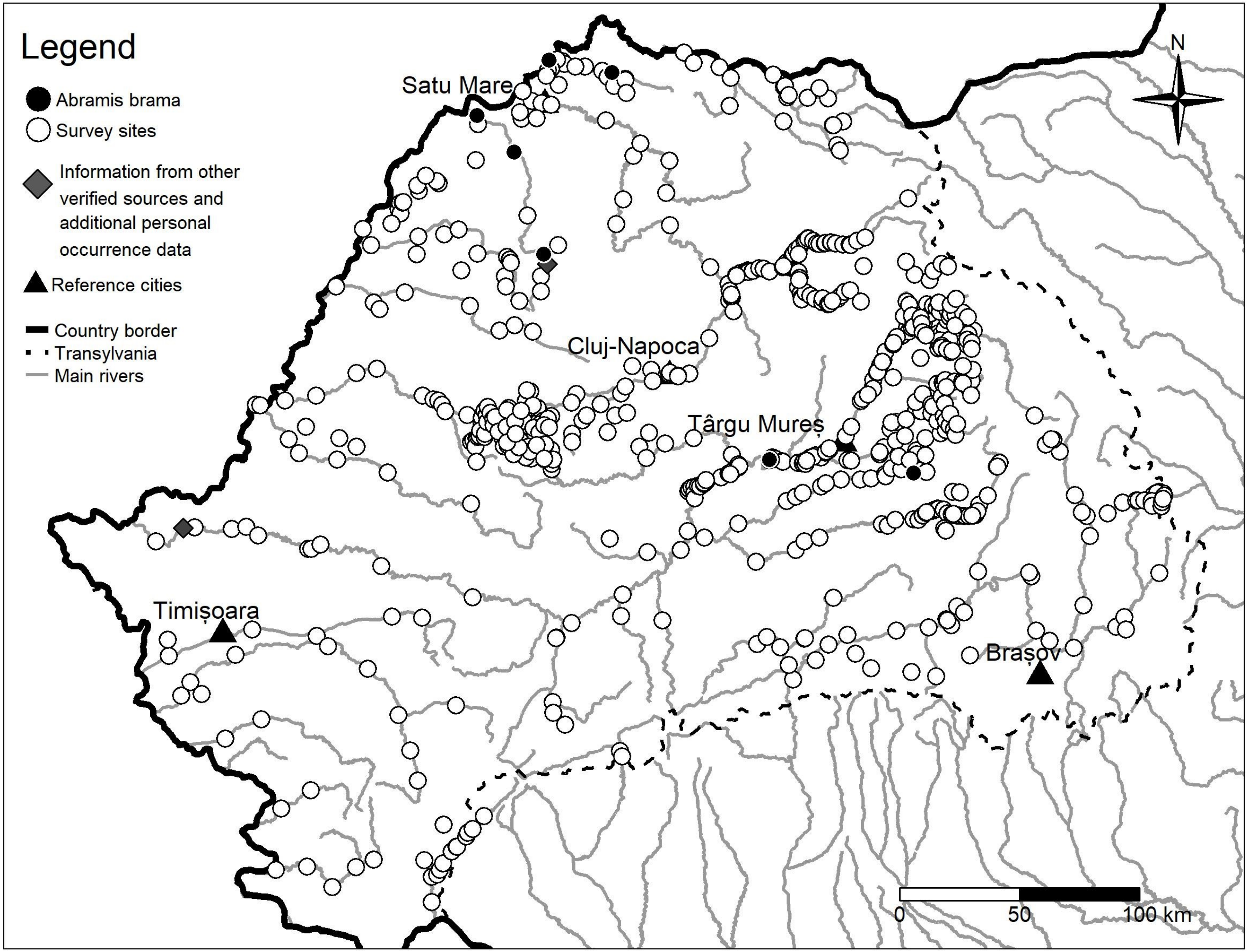

Map S1. Distribution of *Abramis brama*

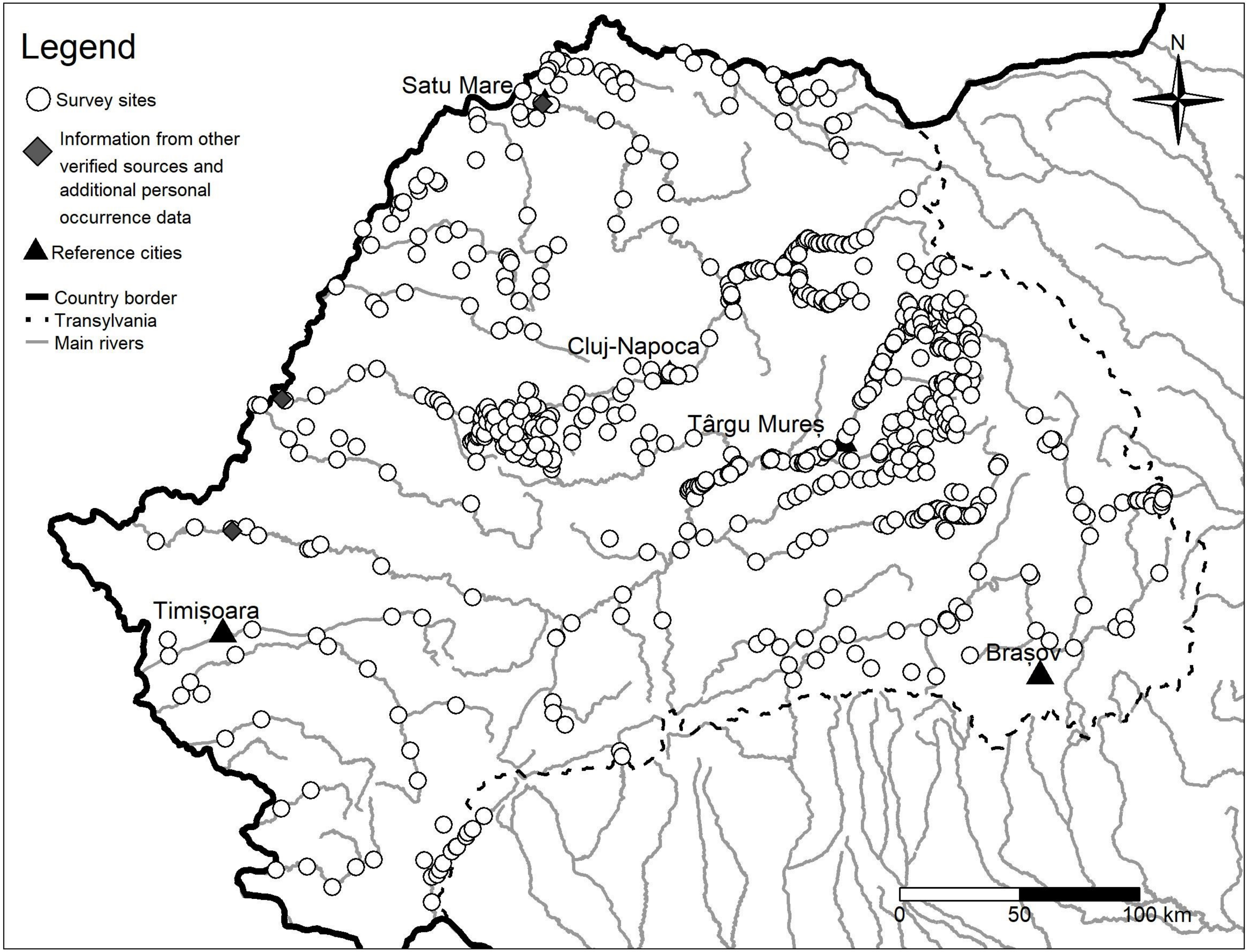

Map S2. Distribution of *Acipenser ruthenus*

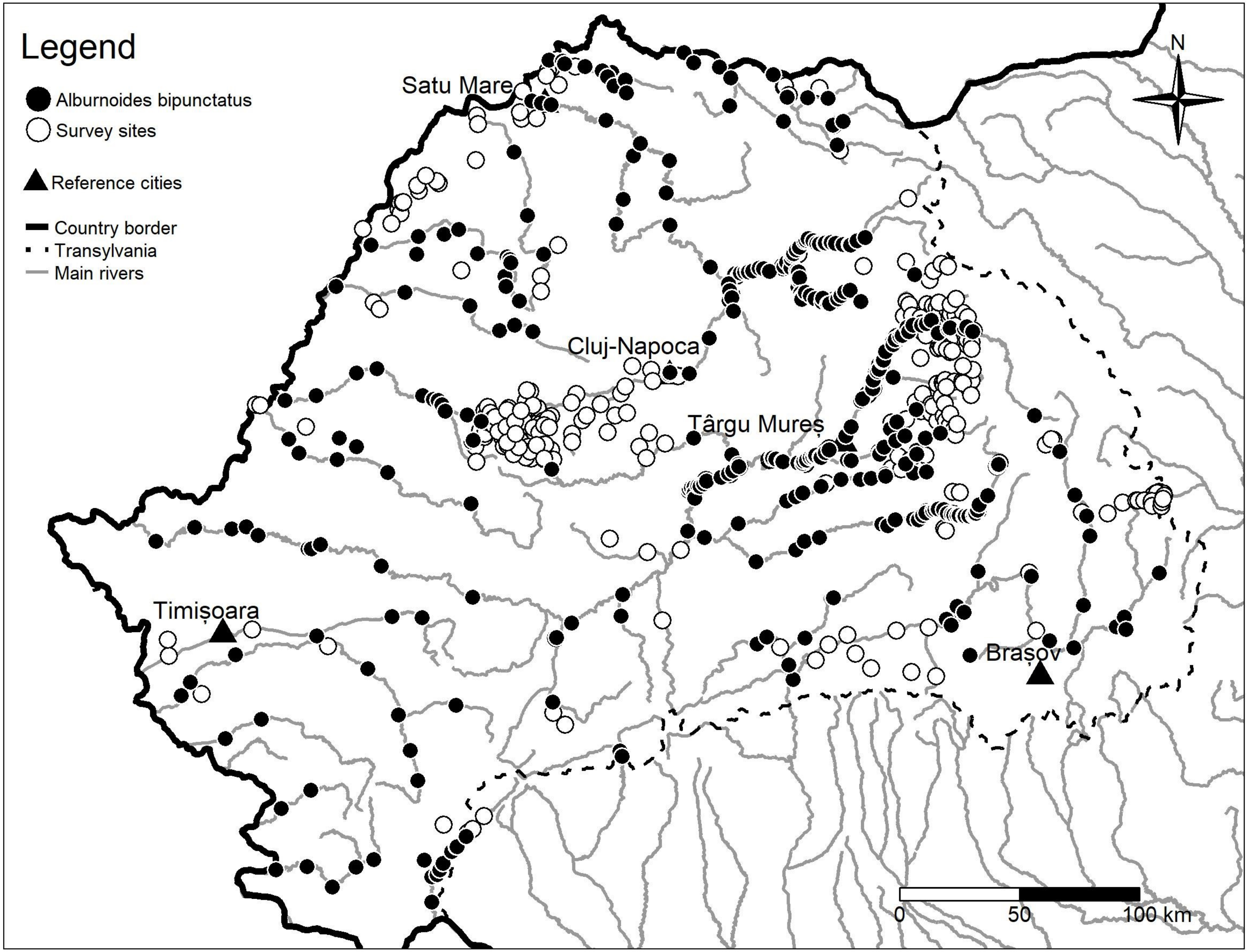

Map S3. Distribution of *Alburnoides bipunctatus*

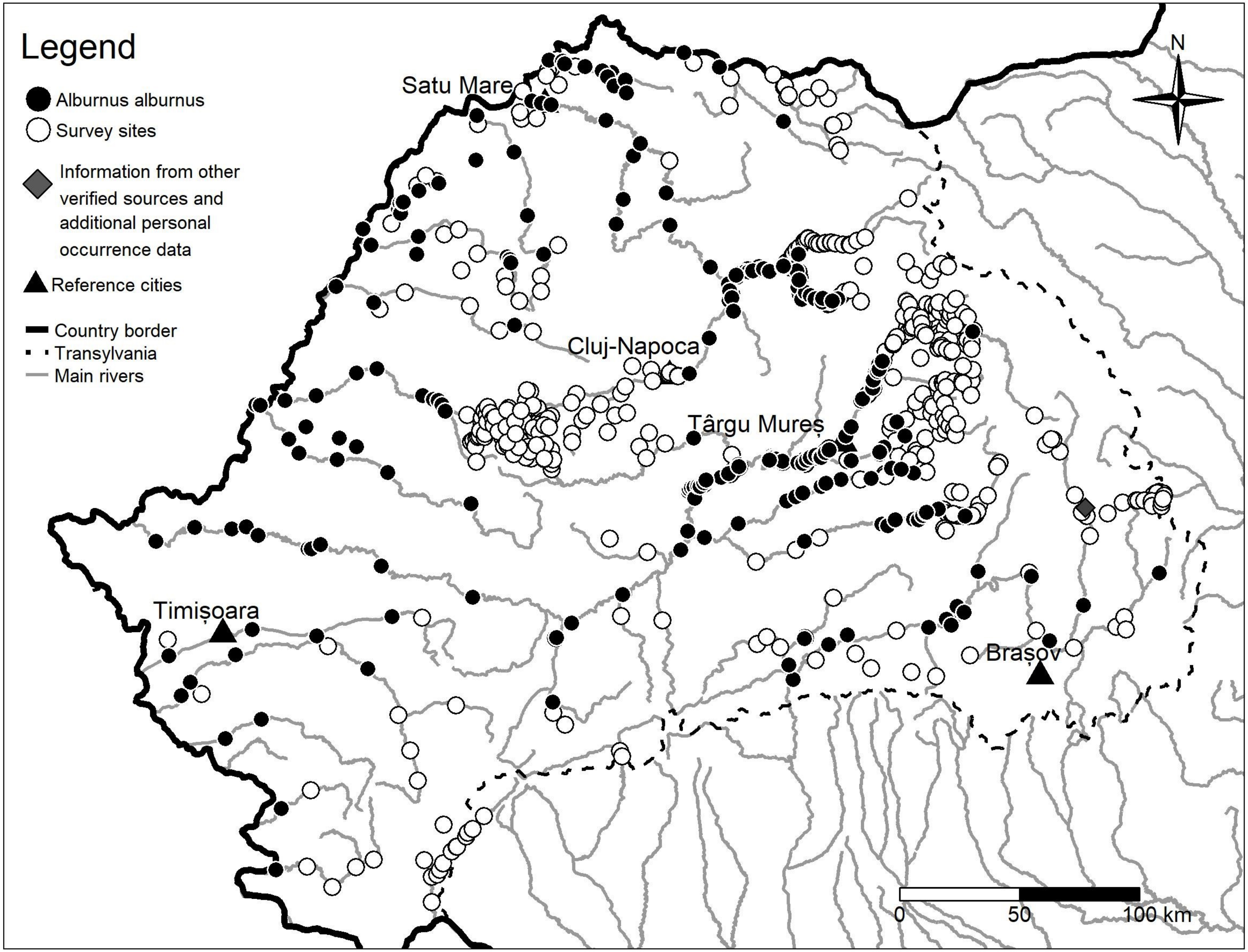

Map S4. Distribution of *Alburnus alburnus*

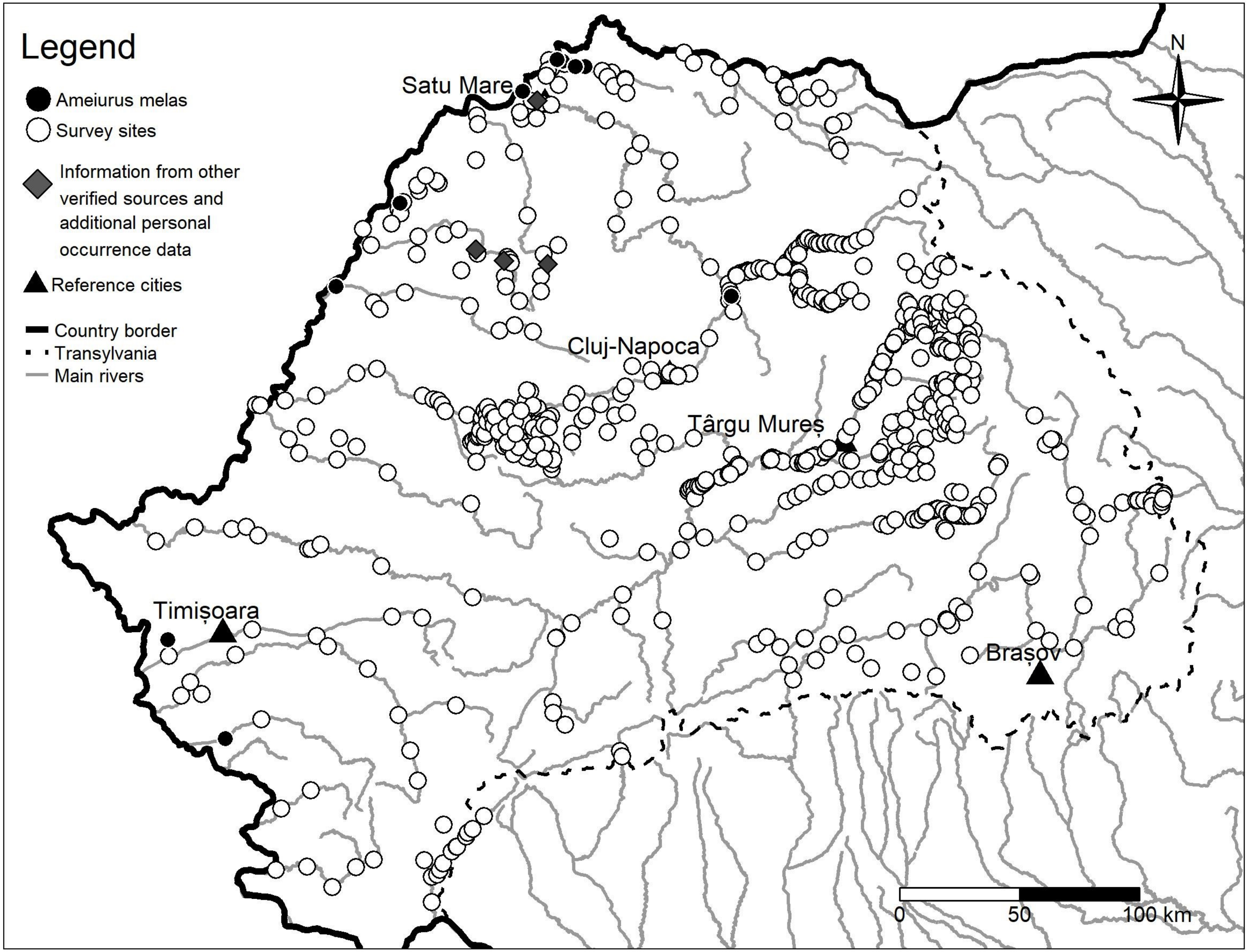

Map S5. Distribution of *Ameiurus melas*

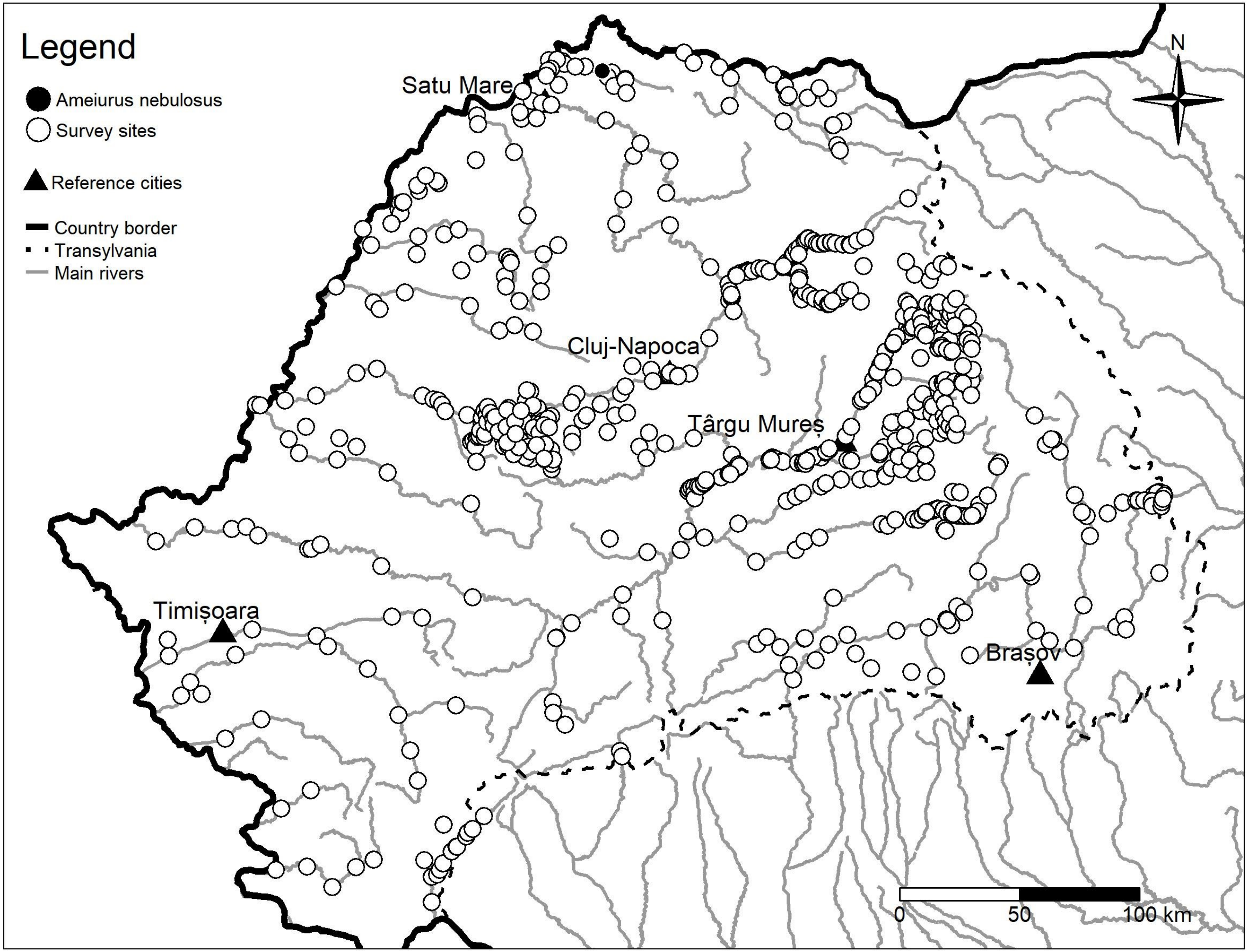

Map S6. Distribution of *Ameiurus nebulosus*

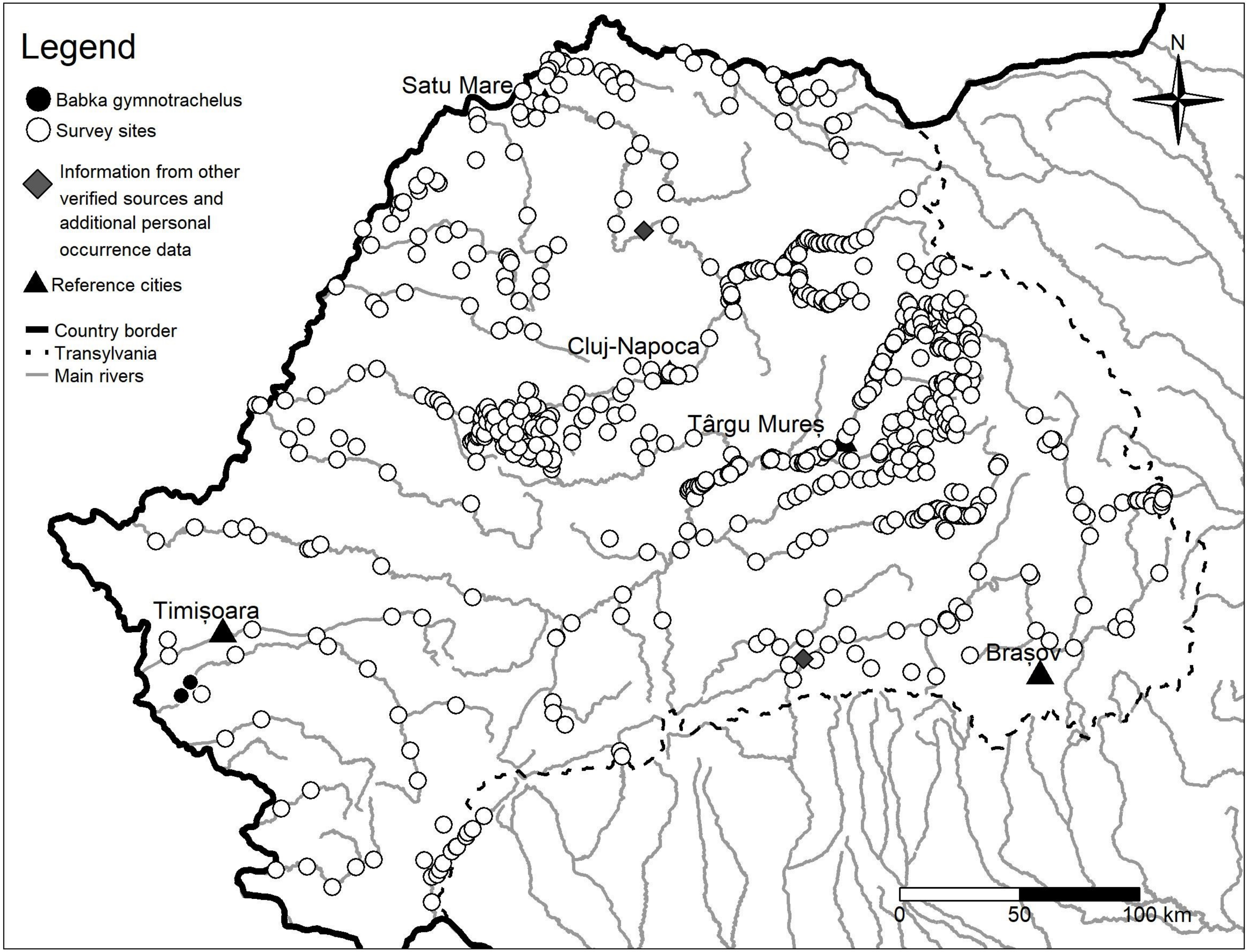

Map S7. Distribution of *Babka gymnotrachelus*

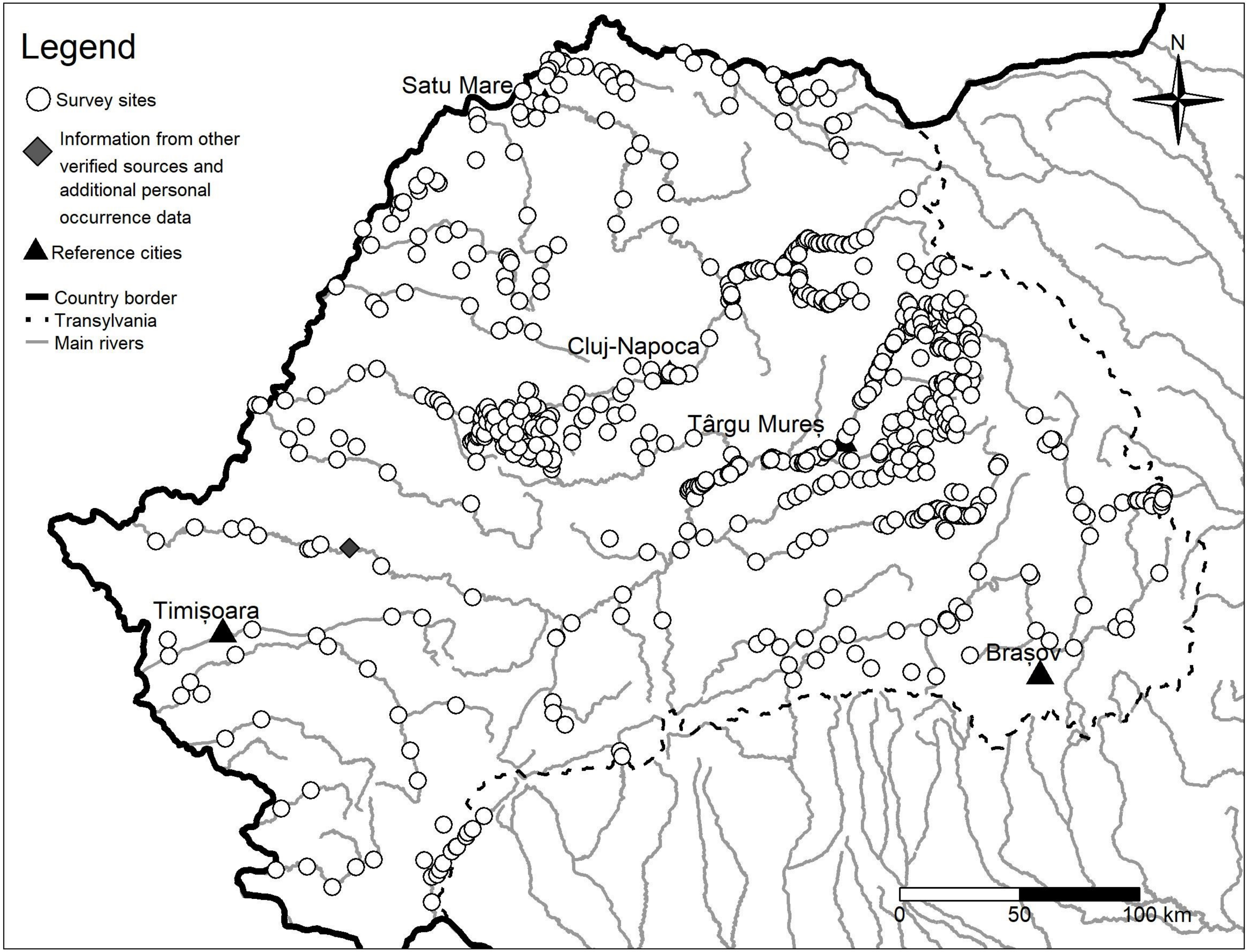

Map S8. Distribution of *Ballerus ballerus*

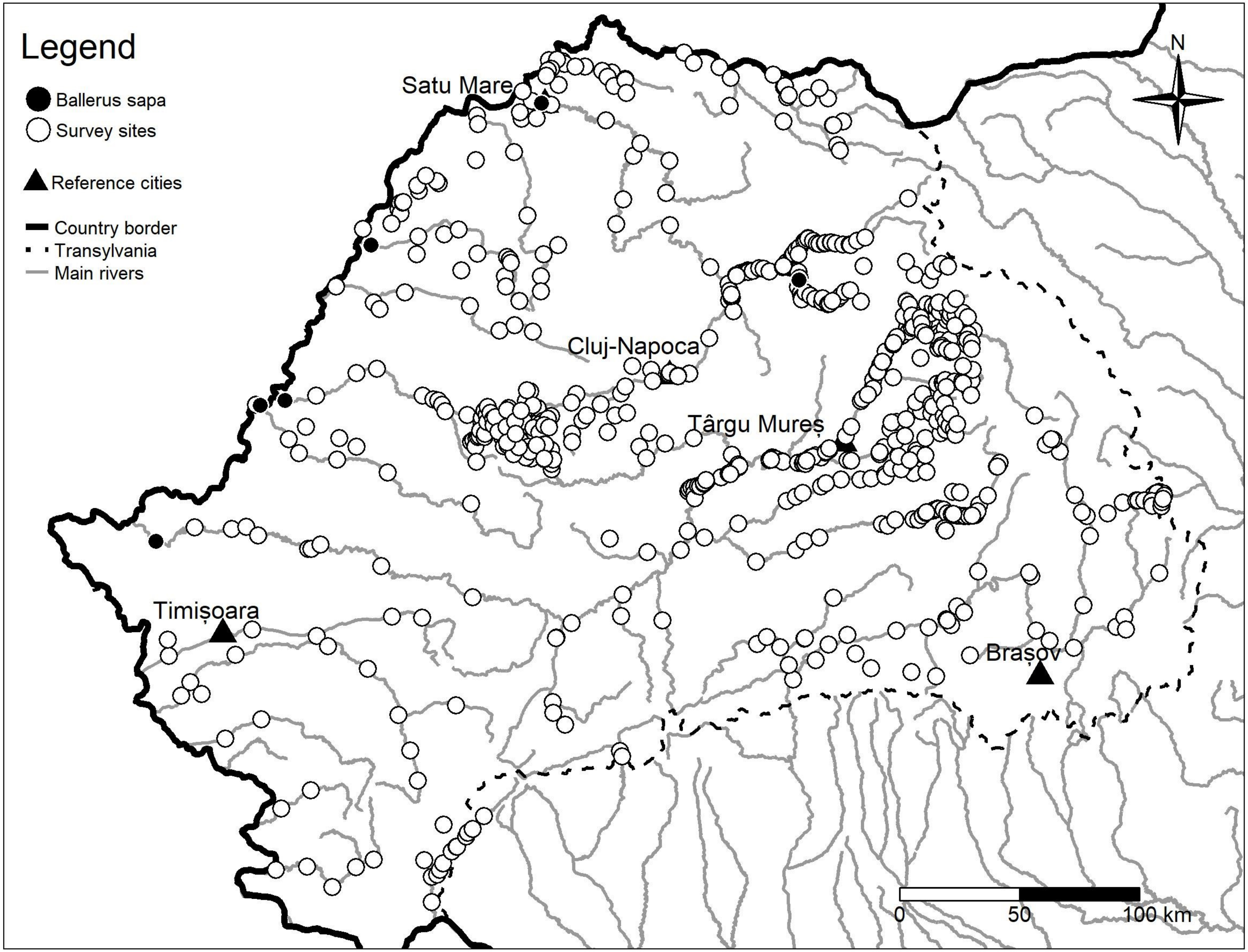

Map S9. Distribution of *Ballerus sapa*

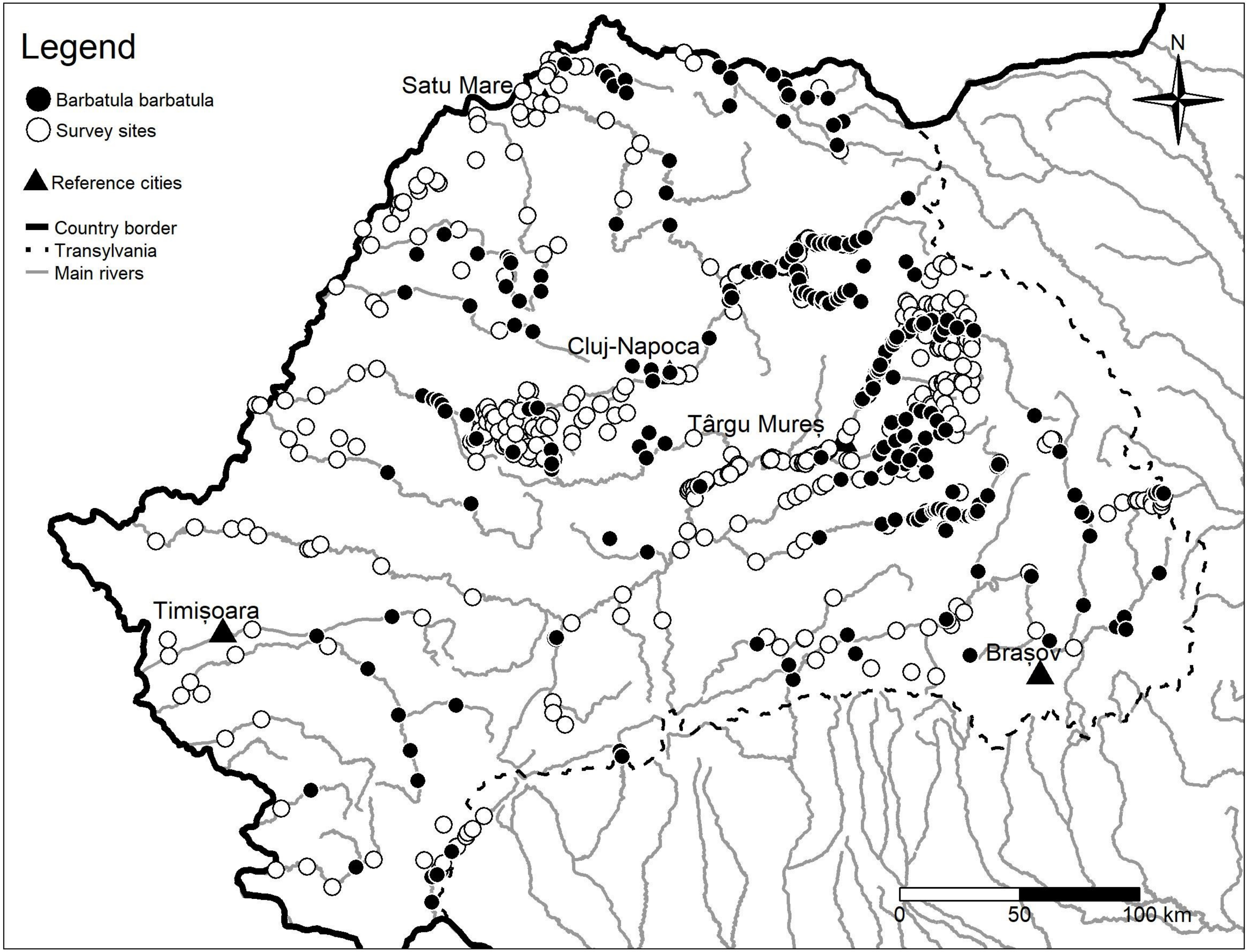

Map S10. Distribution of *Barbatula barbatula*

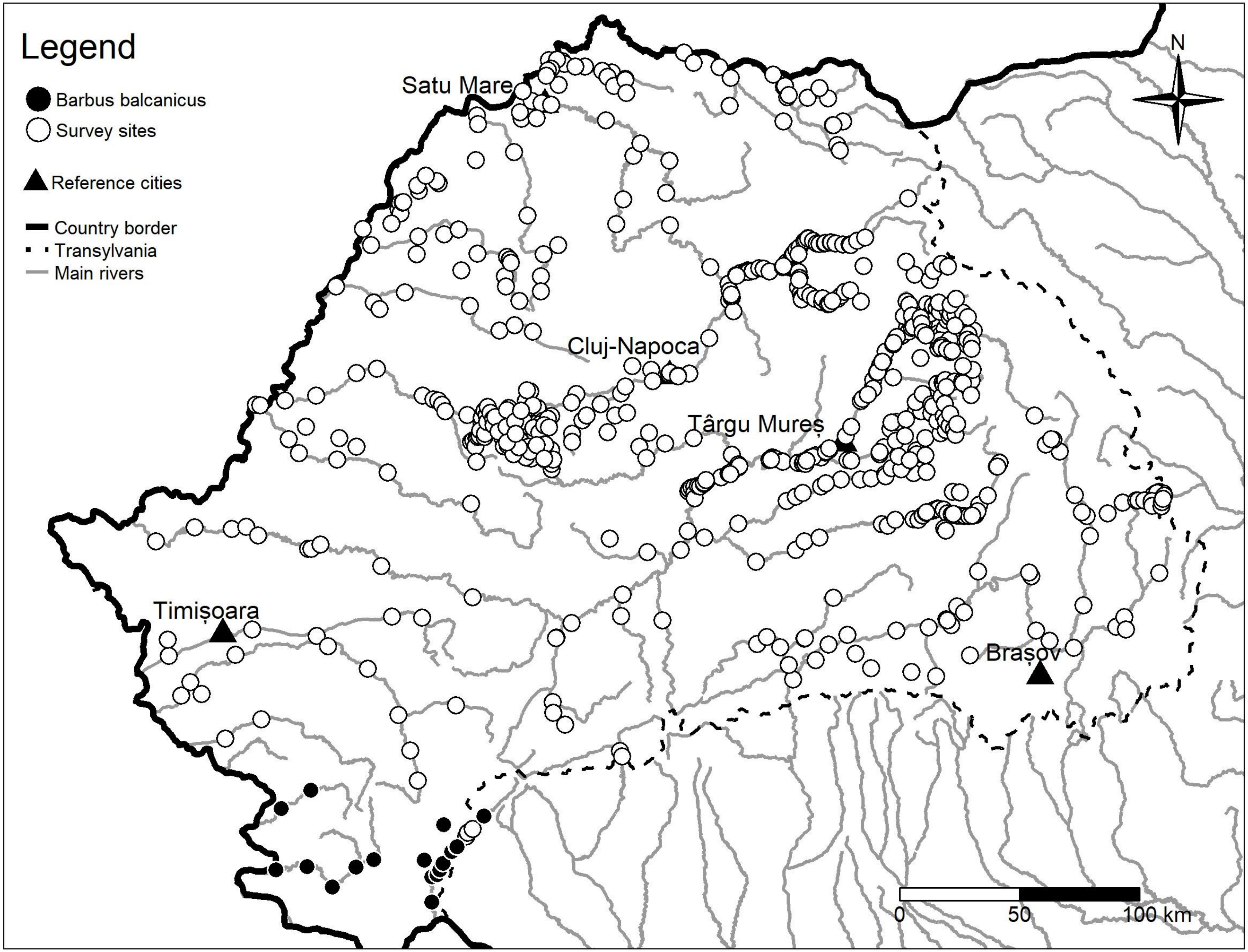

Map S11. Distribution of *Barbus balcanicus*

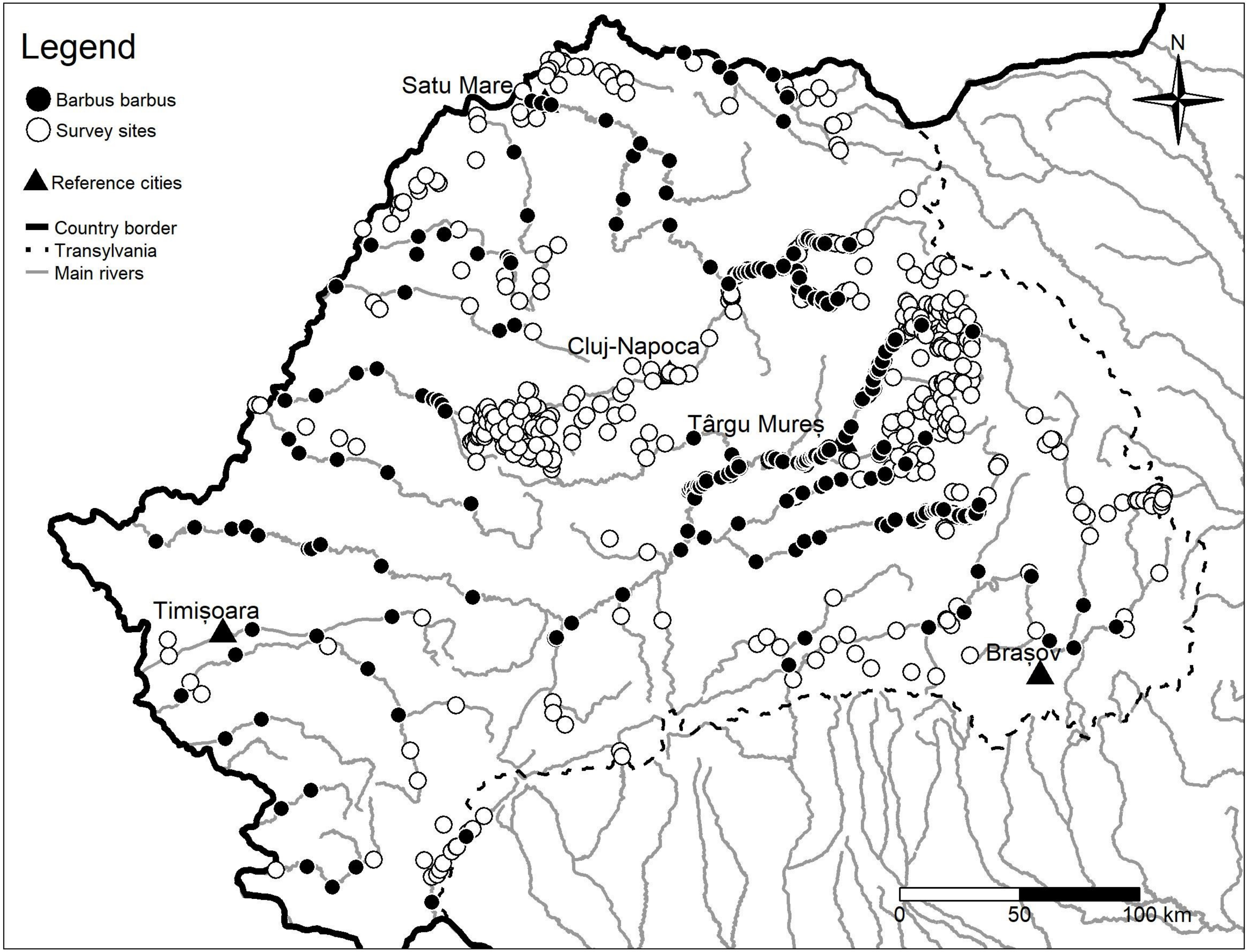

Map S12. Distribution of *Barbus barbus*

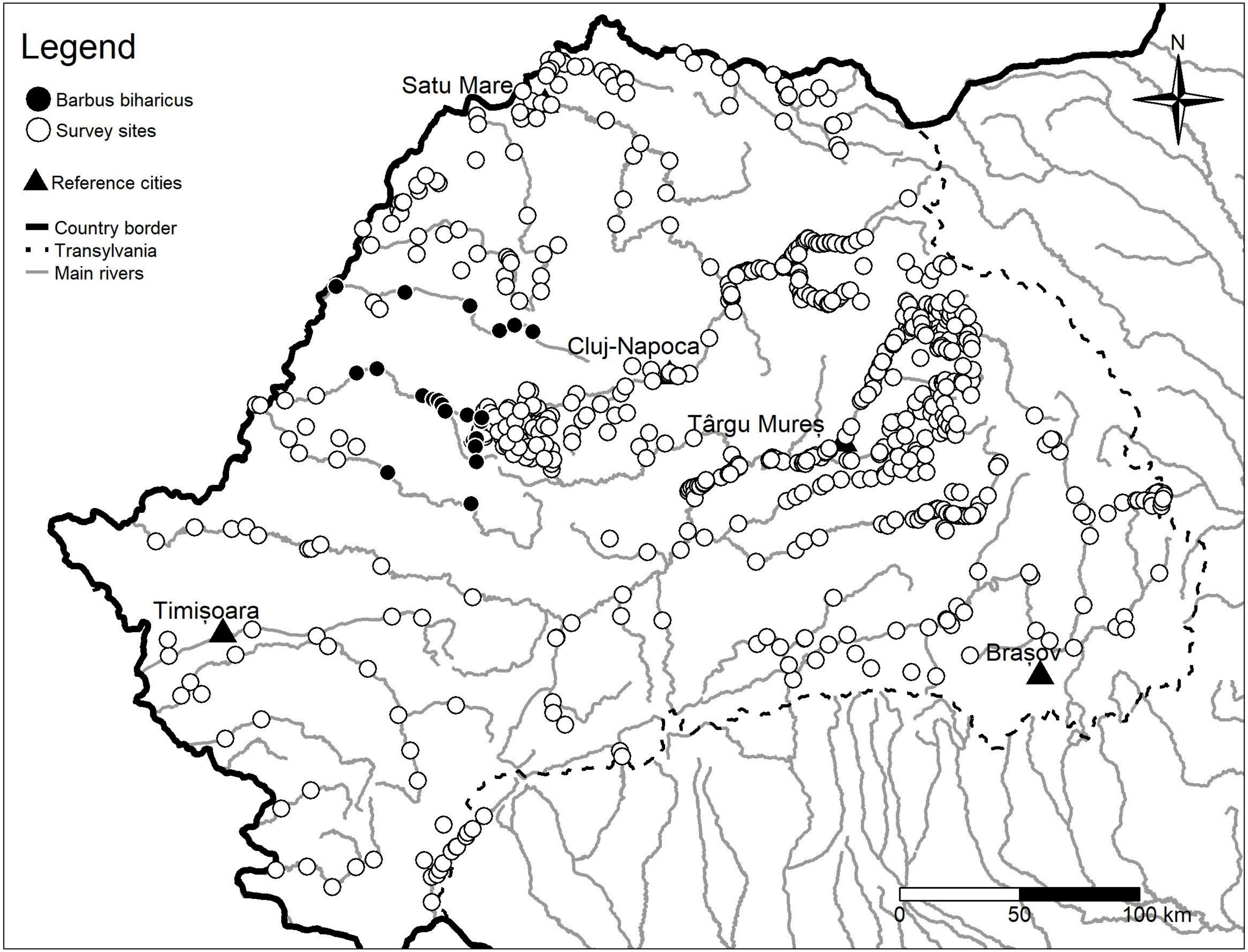

Map S13. Distribution of *Barbus biharicus*

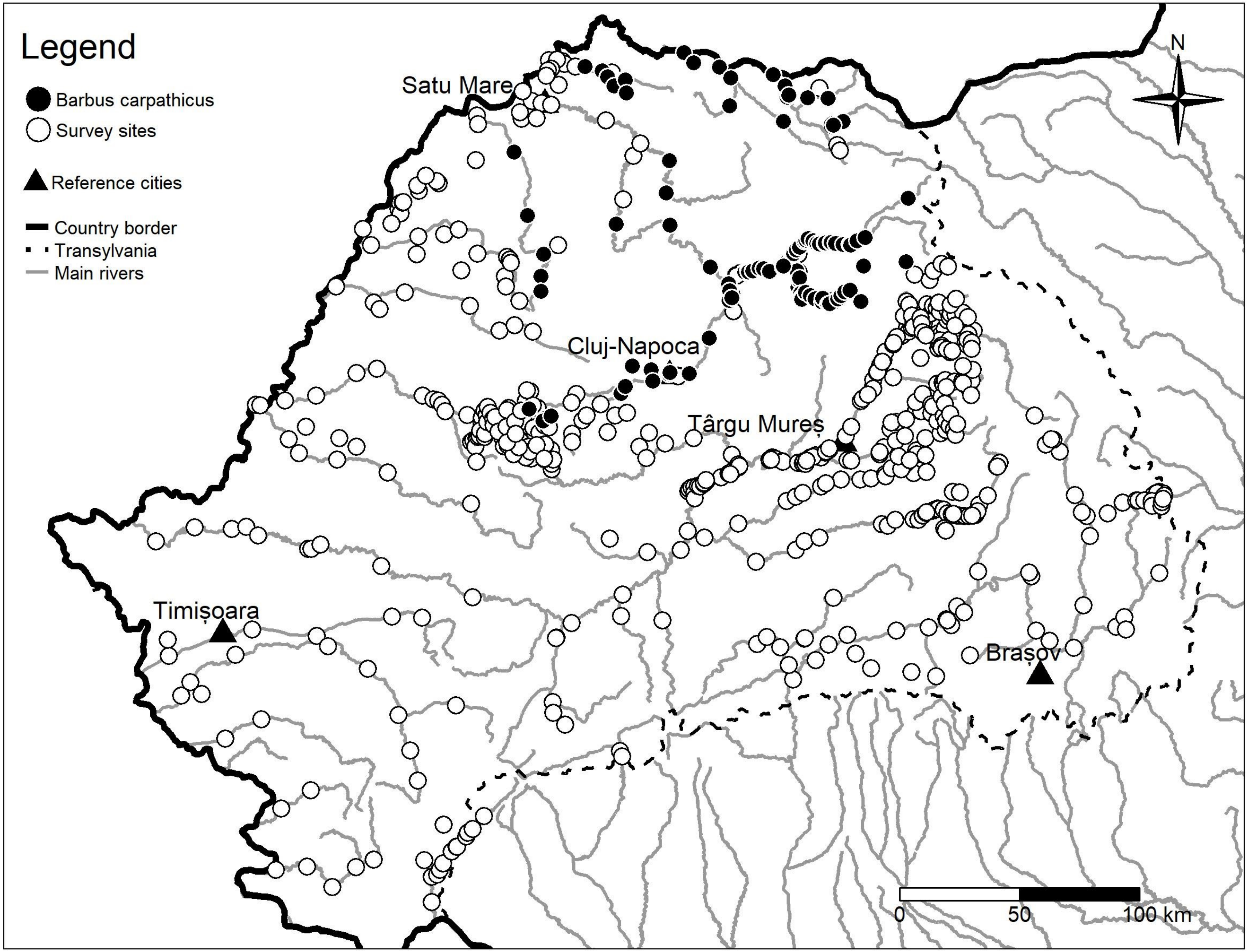

Map S14. Distribution of *Barbus carpathicus*

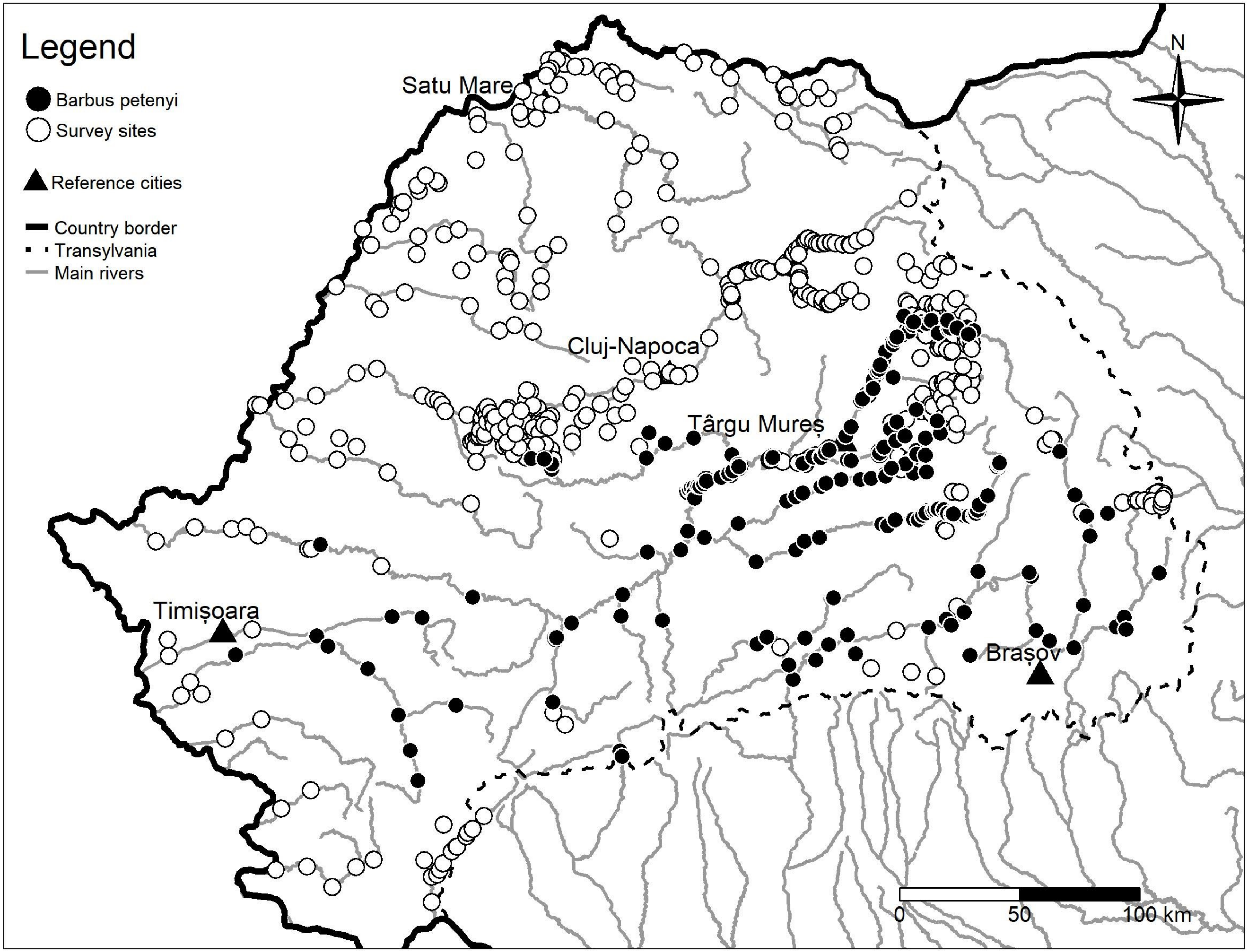

Map S15. Distribution of *Barbus petenyi*

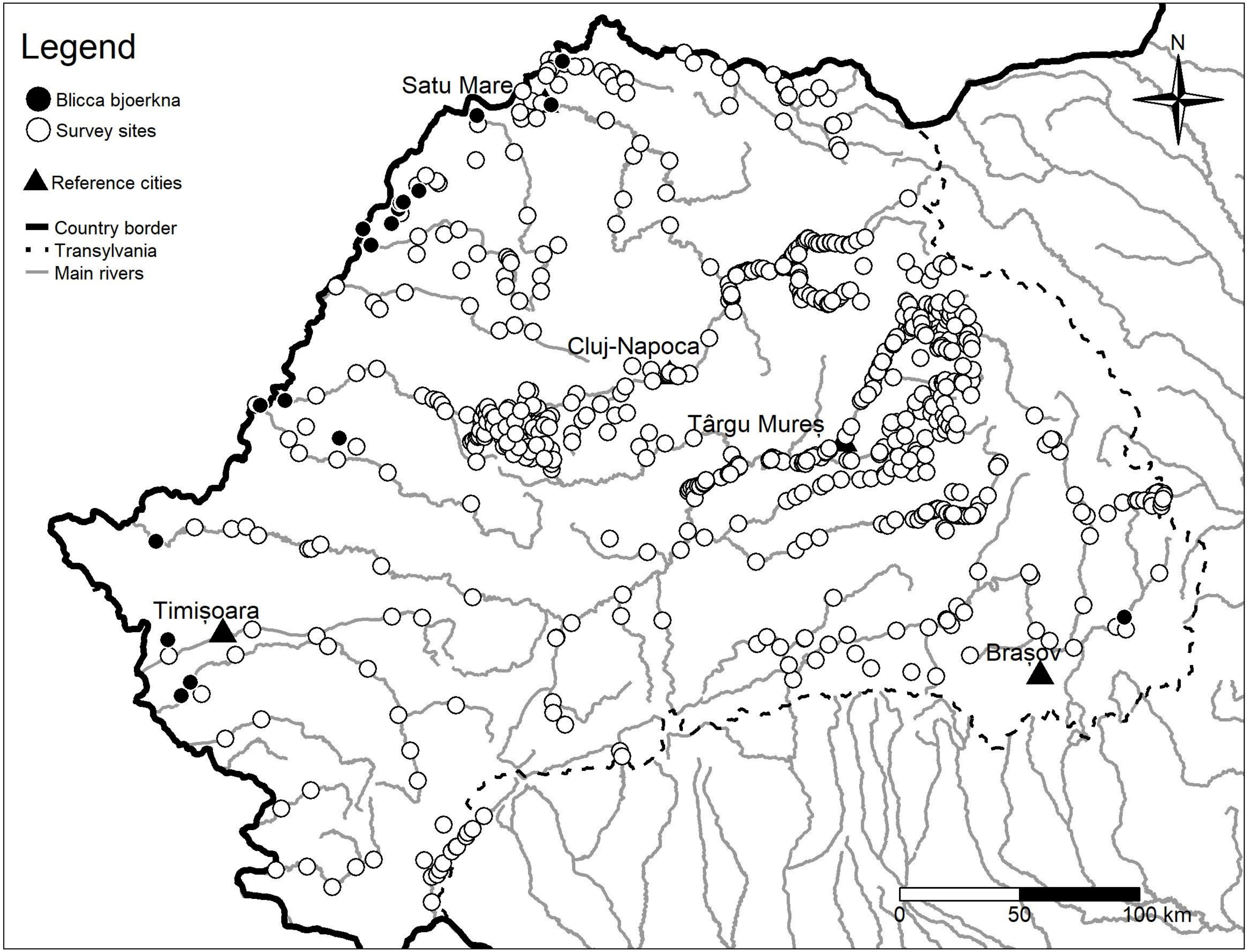

Map S16. Distribution of *Blicca bjoerkna*

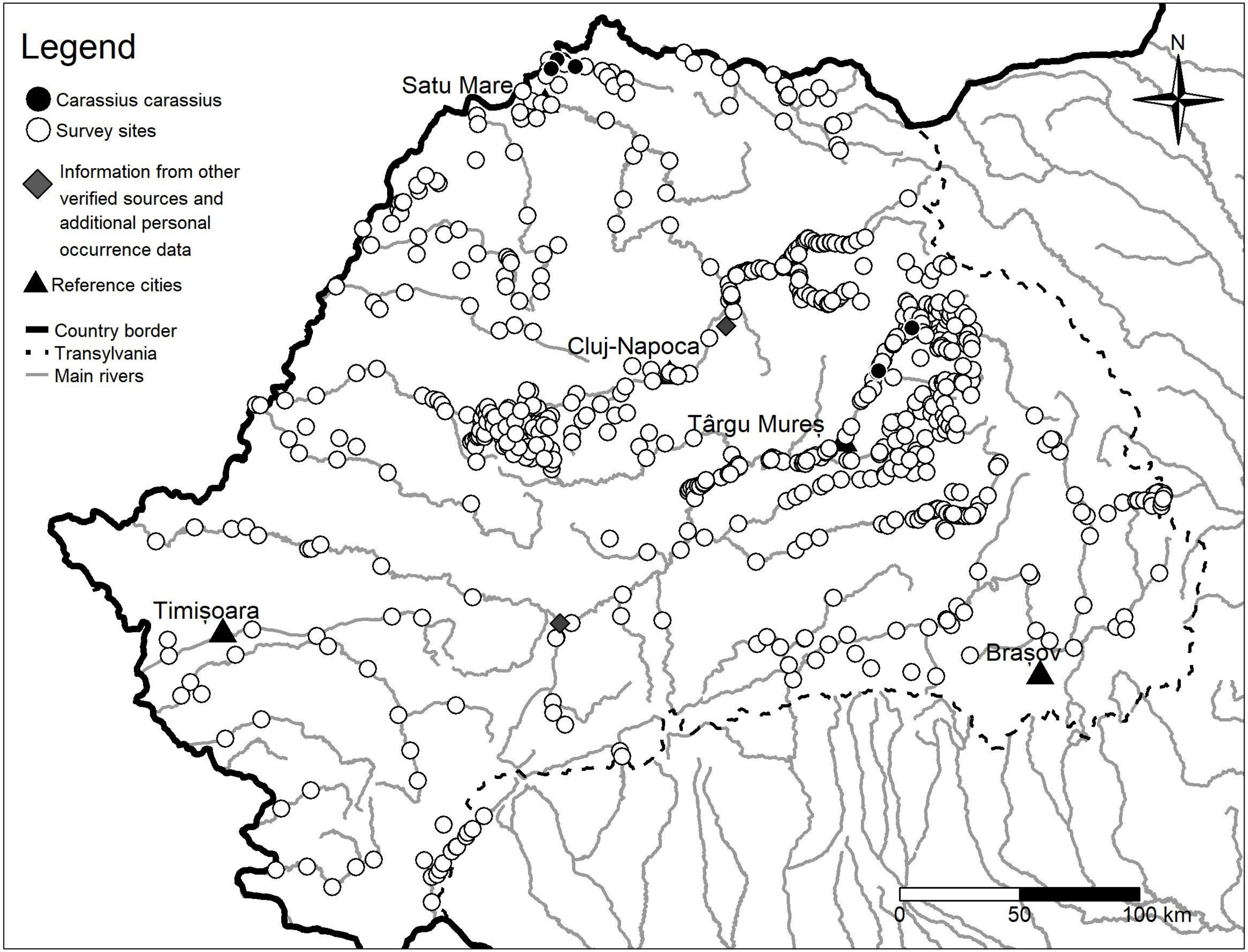

Map S17. Distribution of *Carassius carassius*

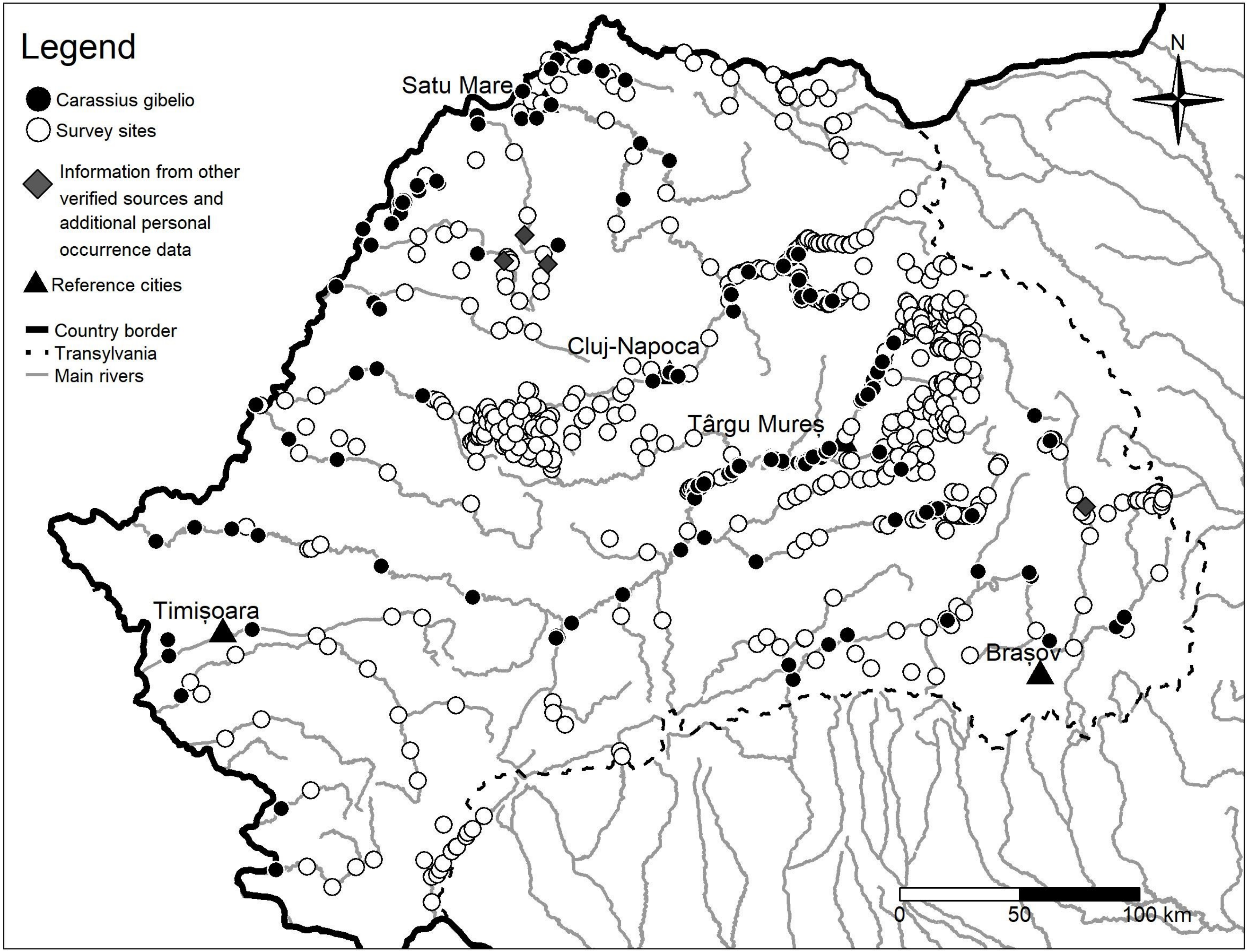

Map S18. Distribution of *Carassius gibelio*

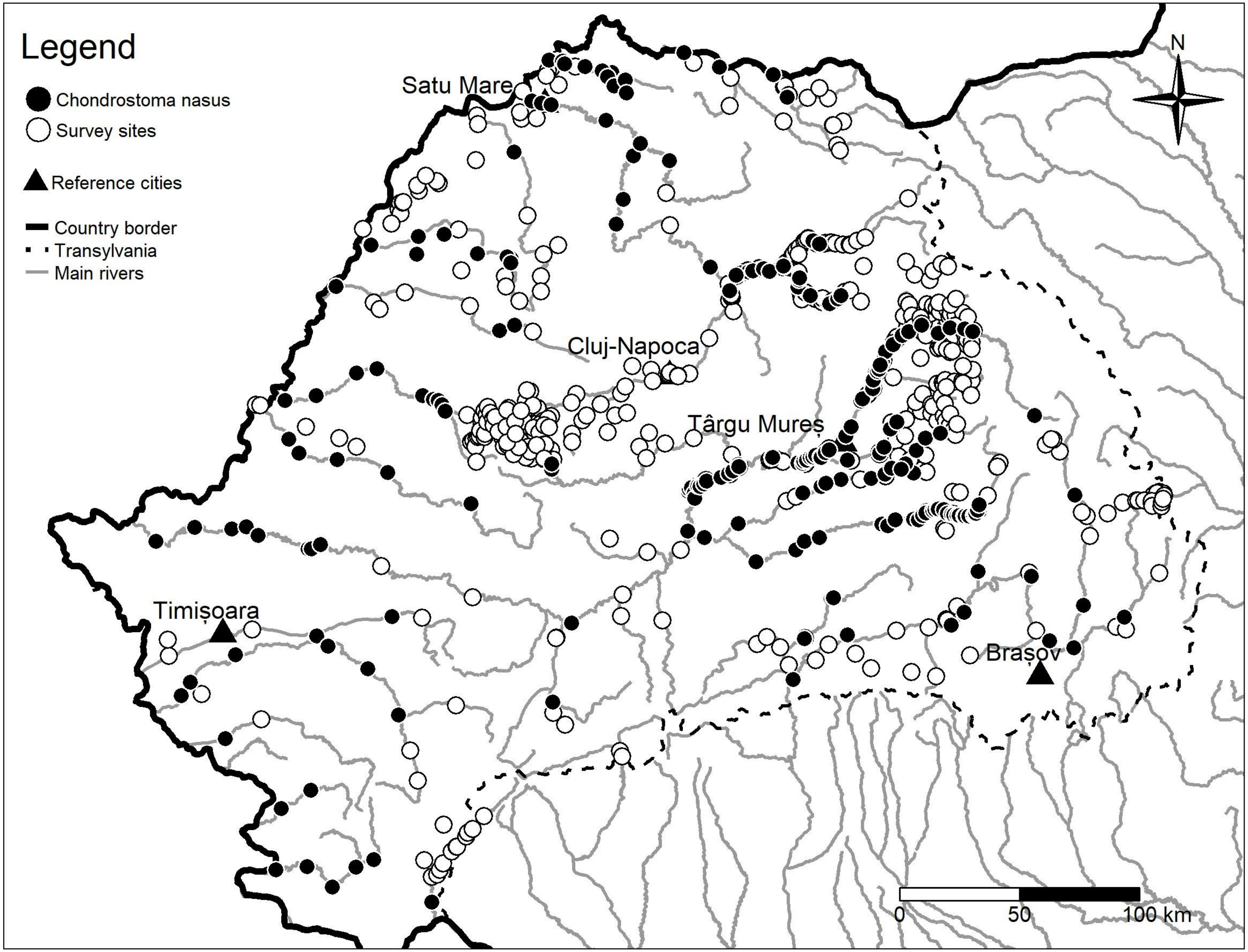

Map S19. Distribution of *Chondrostoma nasus*

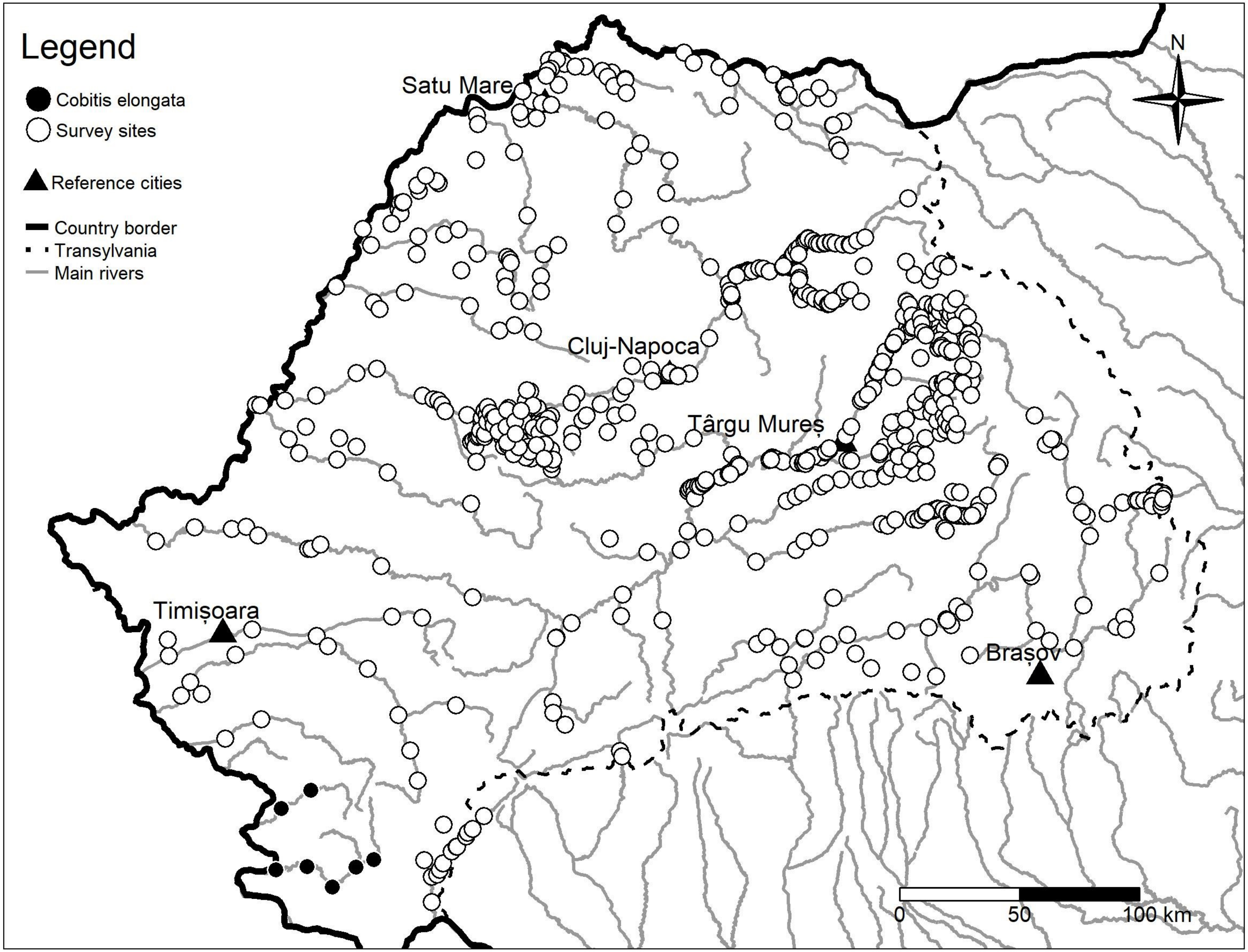

Map S20. Distribution of *Cobitis elongata*

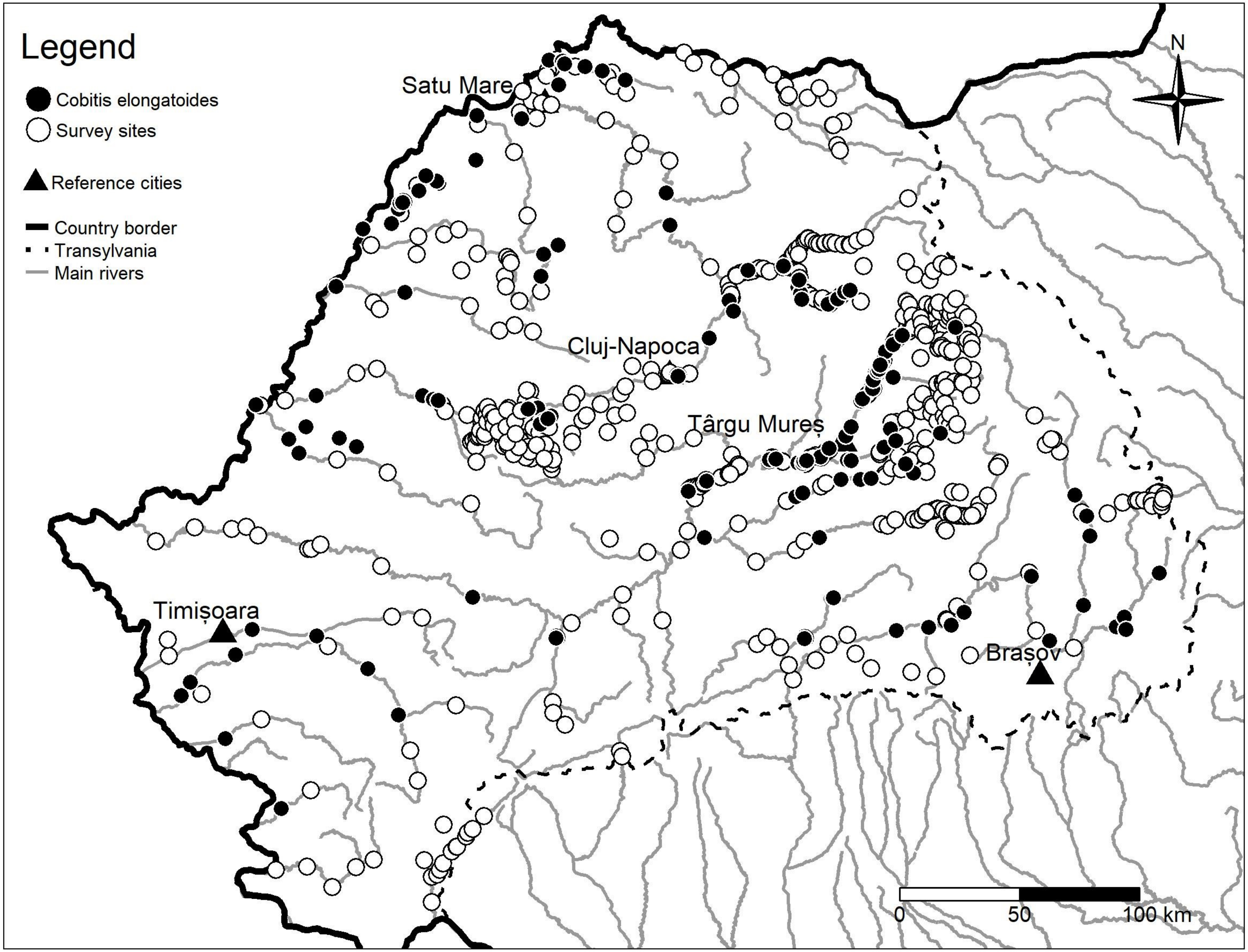

Map S21. Distribution of *Cobitis elongatoides*

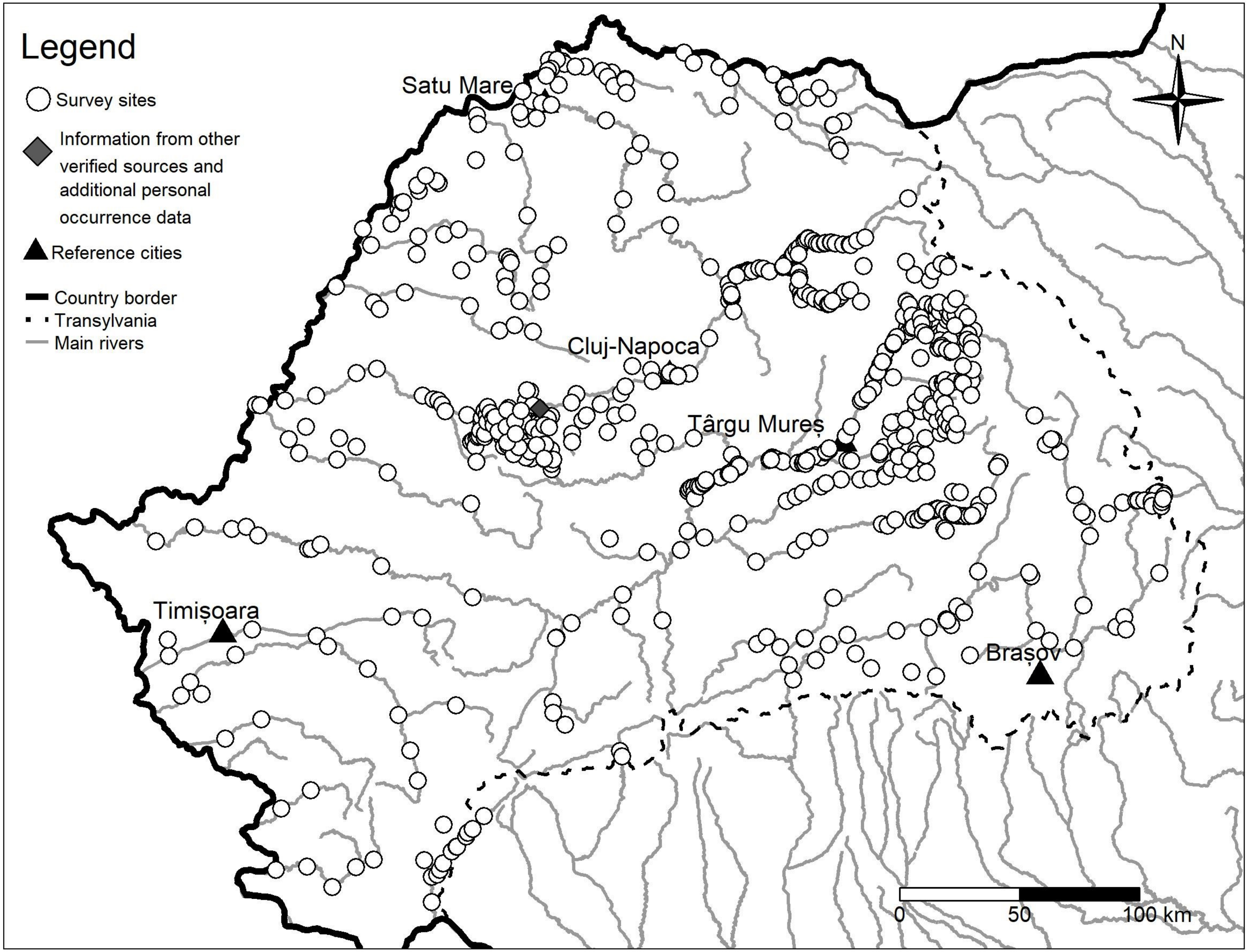

Map S22. Distribution of *Coregonus sp*

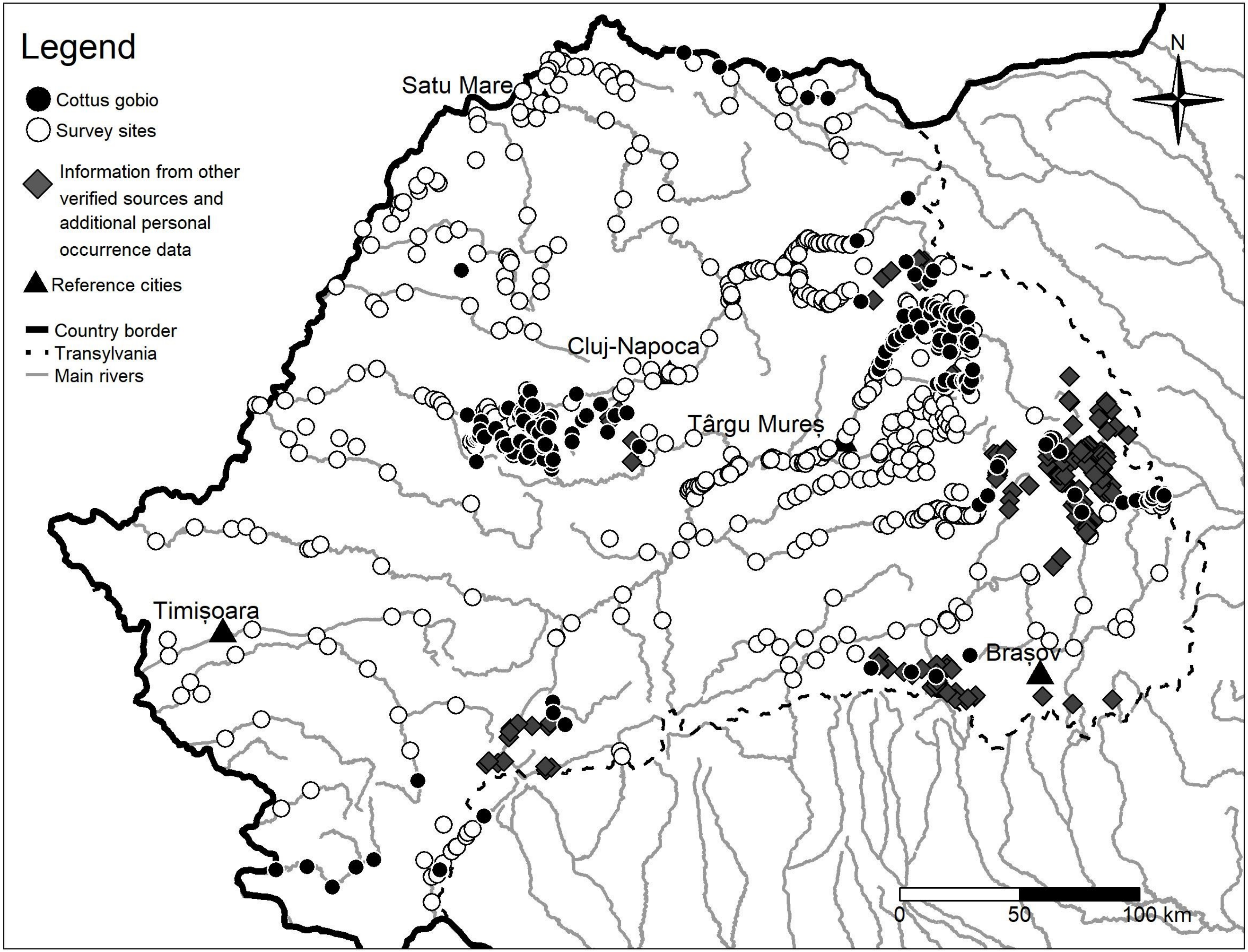

Map S23. Distribution of *Cottus gobio*

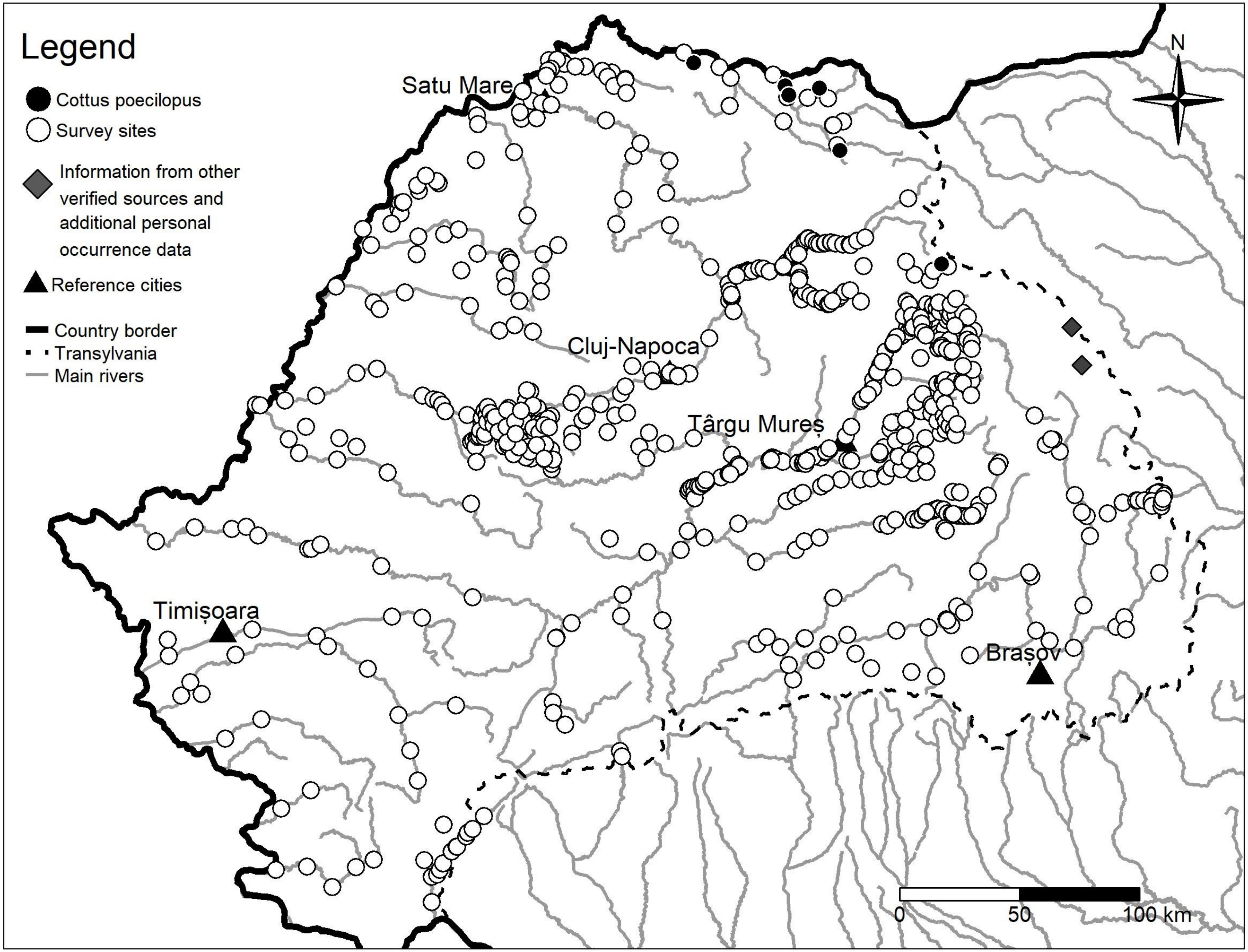

Map S24. Distribution of *Cottus poecilopus*

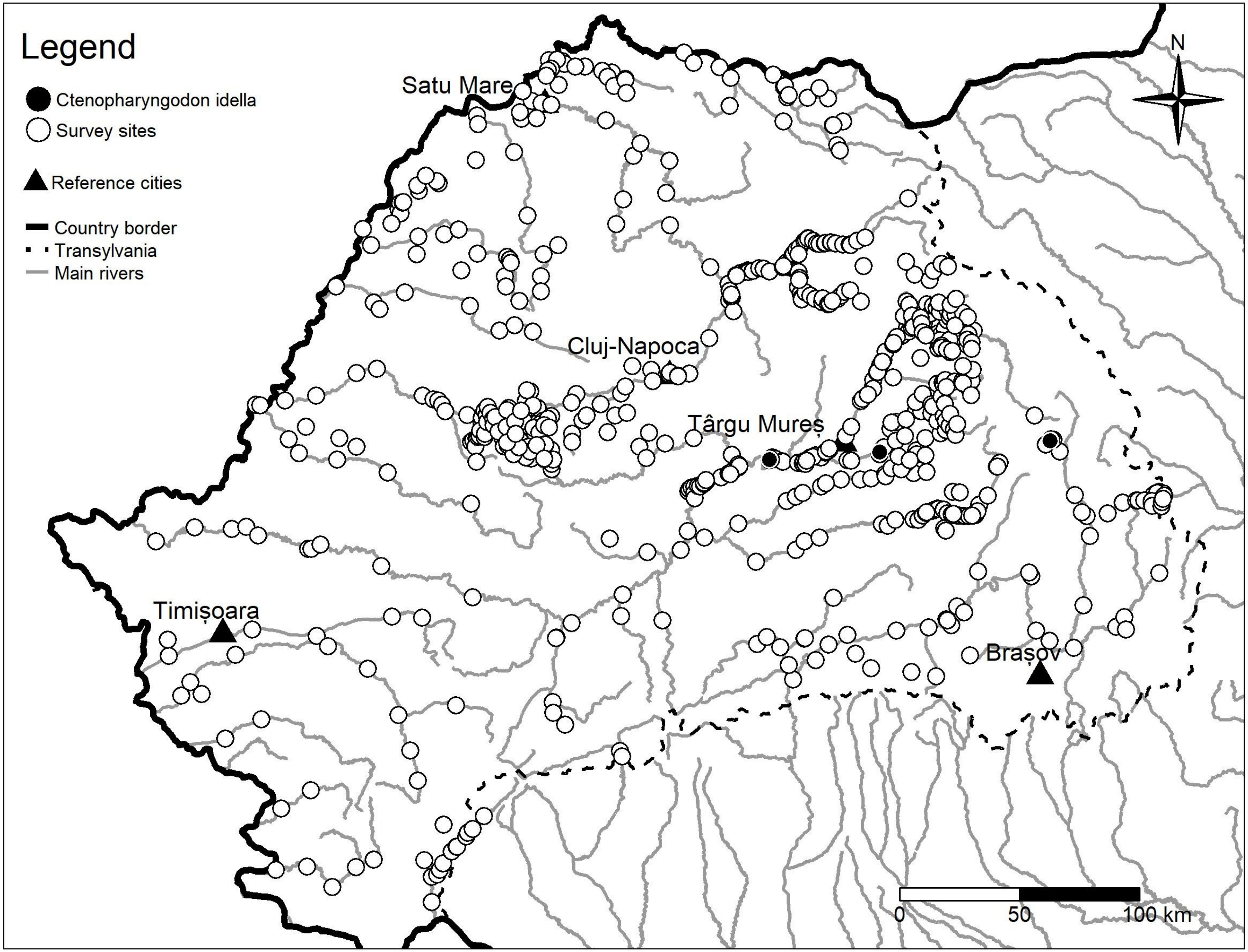

Map S25. Distribution of *Ctenopharyngodon idella*

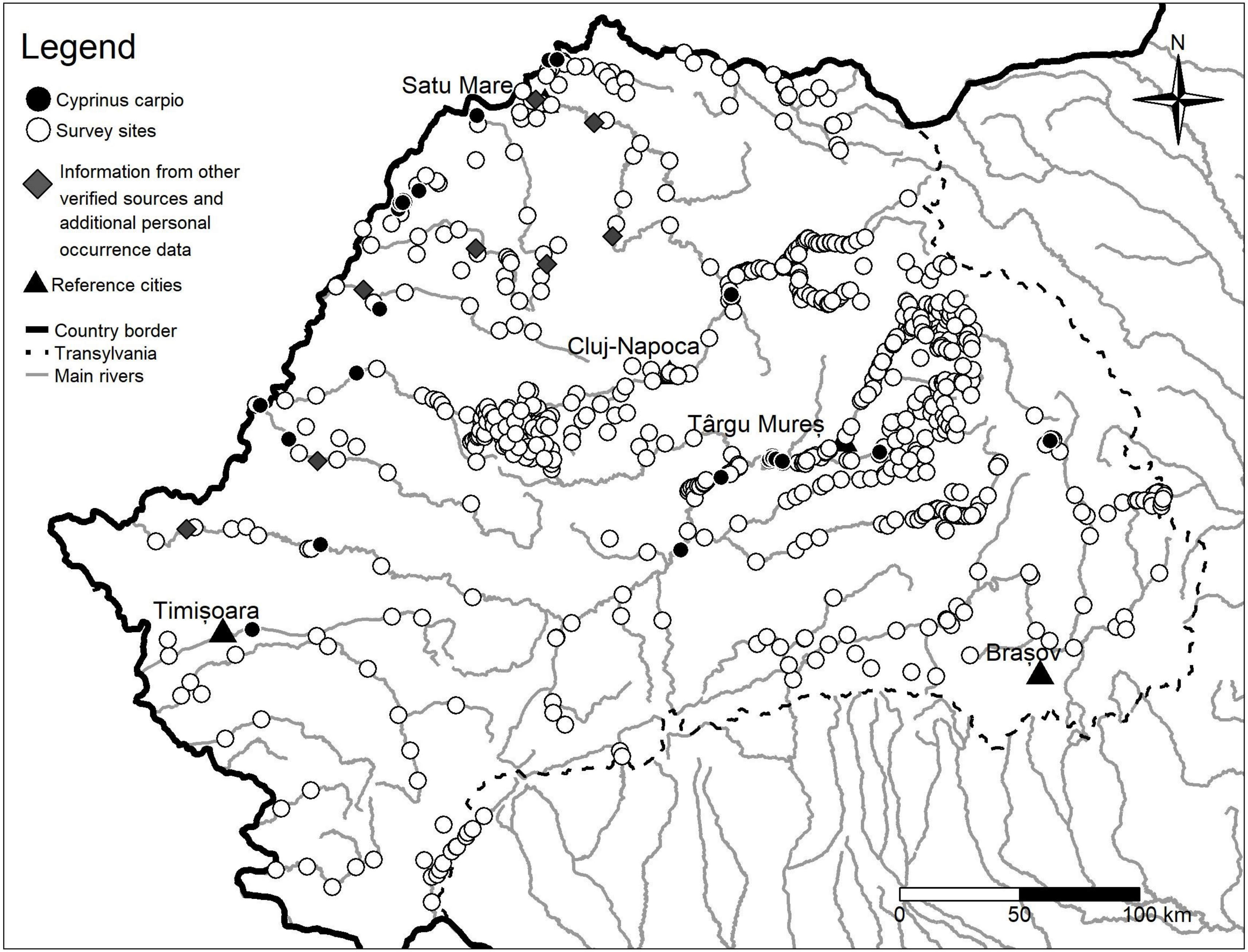

Map S26. Distribution of *Cyprinus carpio*

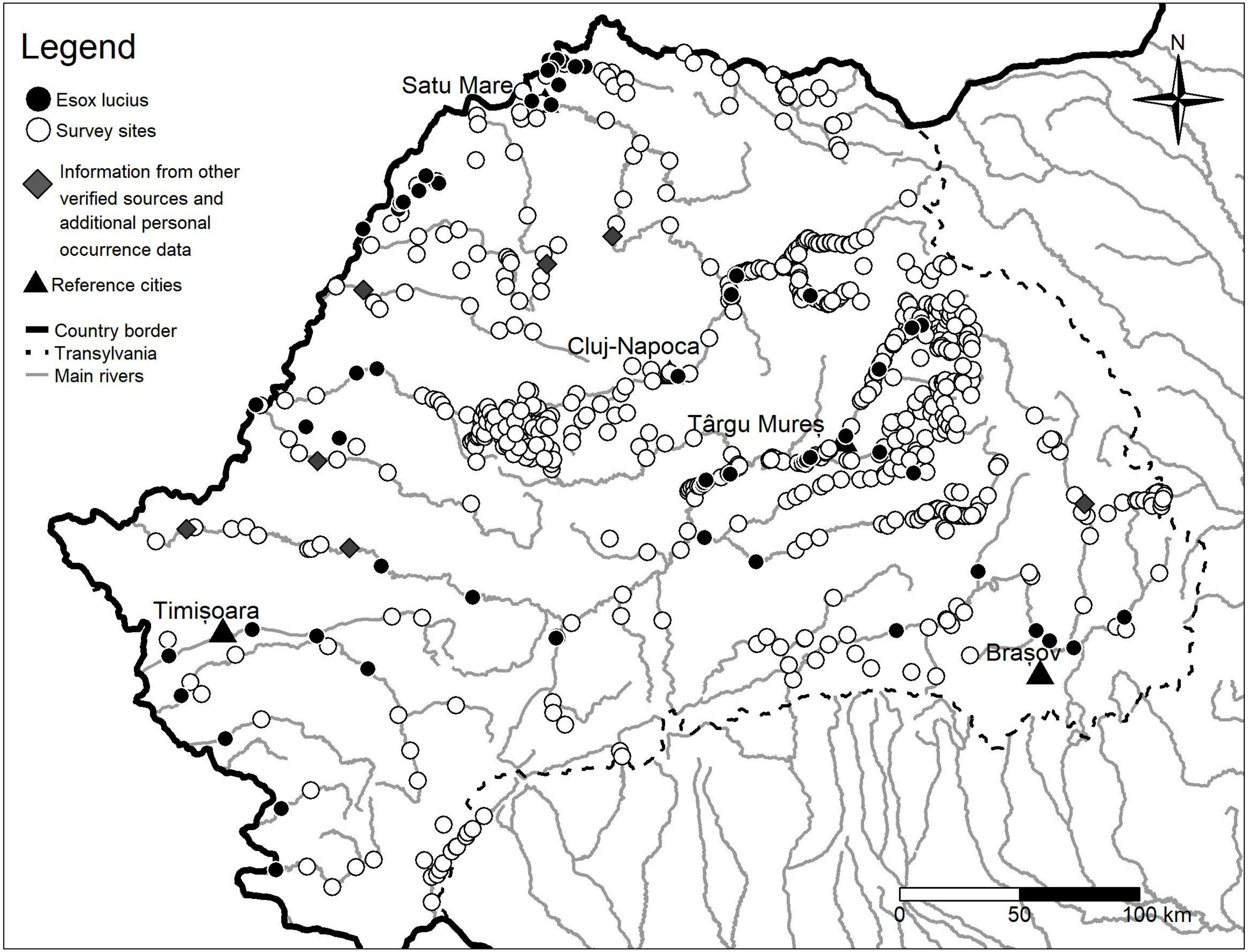

Map S27. Distribution of *Esox lucius*

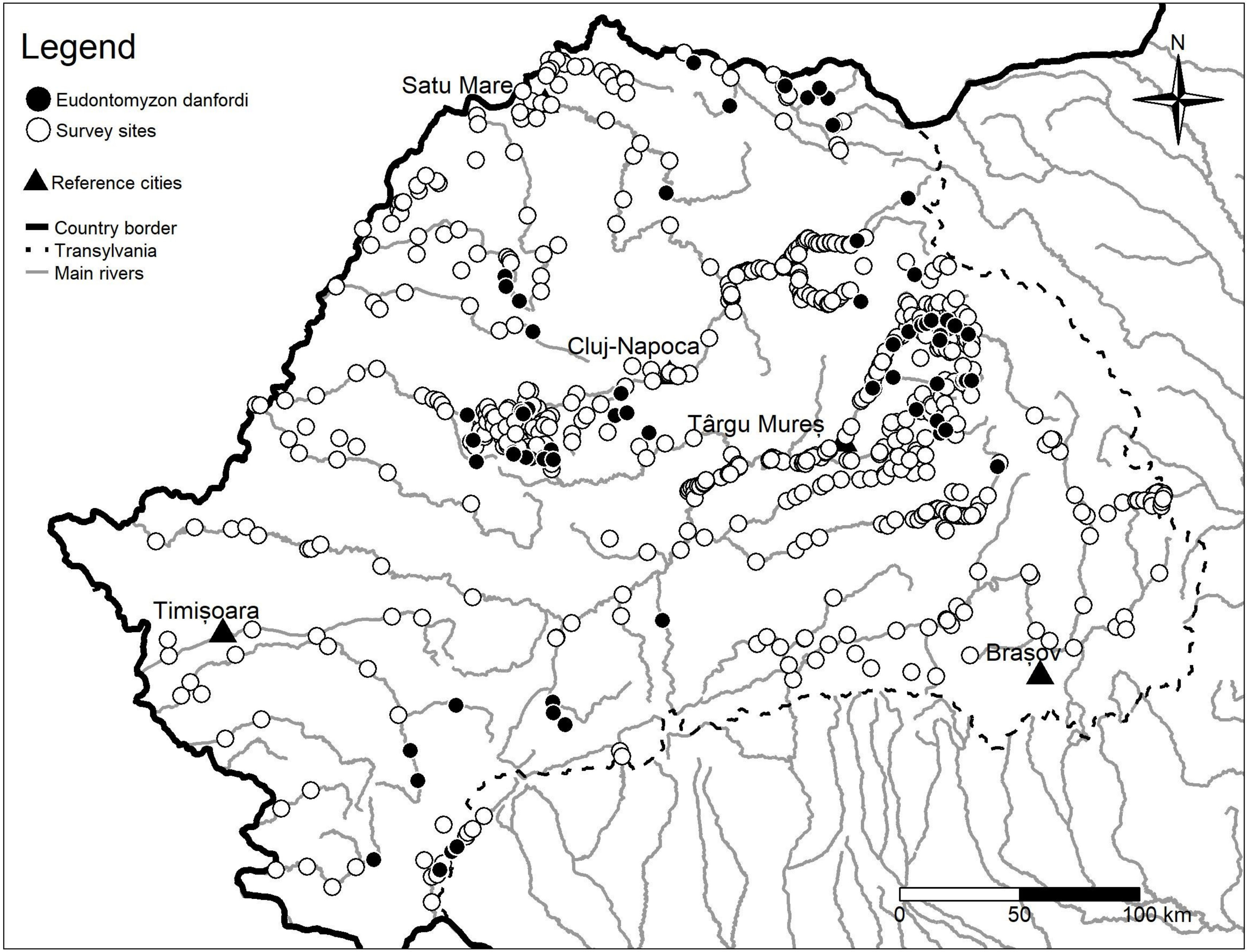

Map S28. Distribution of *Eudontomyzon danfordi*

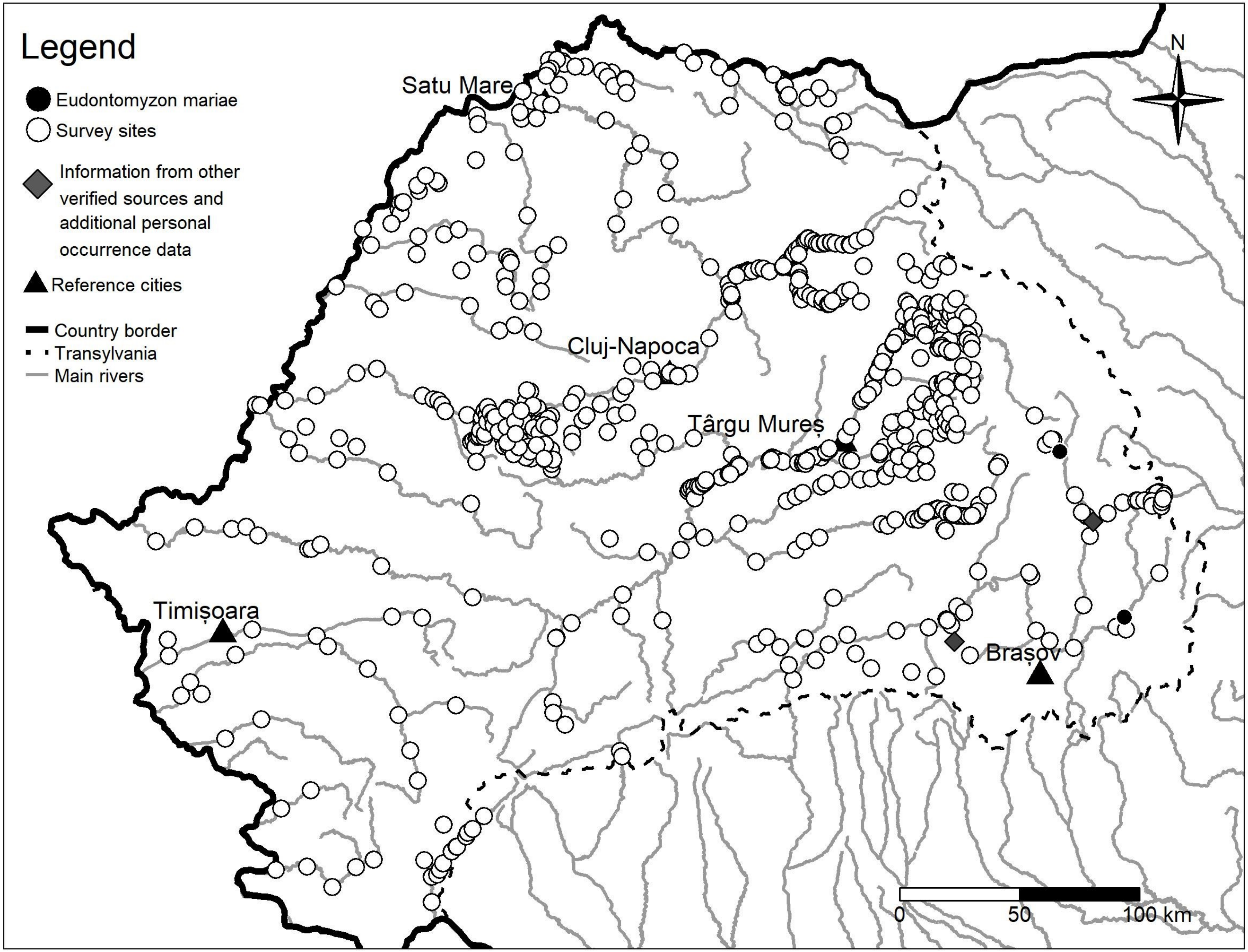

Map S29. Distribution of *Eudontomyzon mariae*

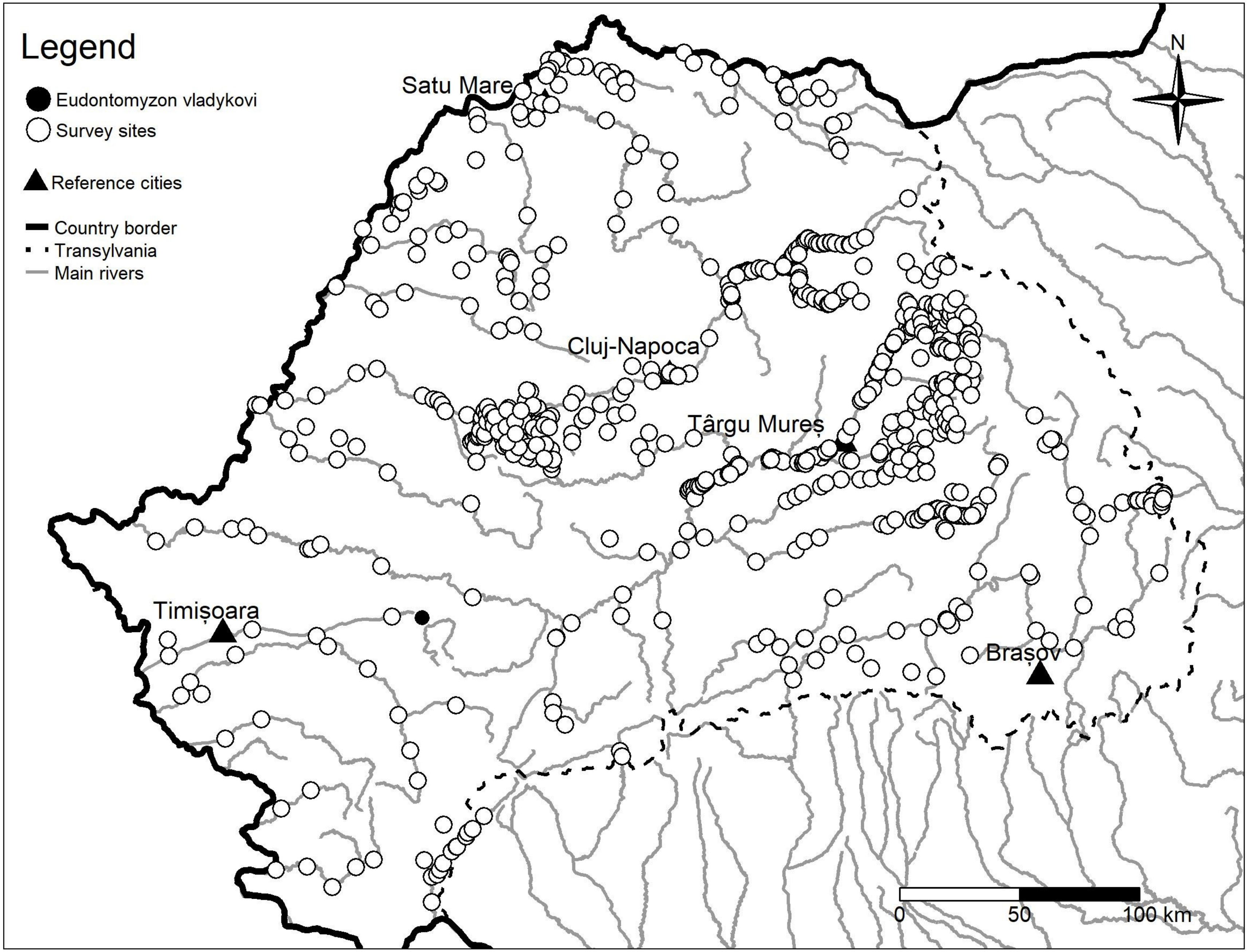

Map S30. Distribution of *Eudontomyzon vladykovi*

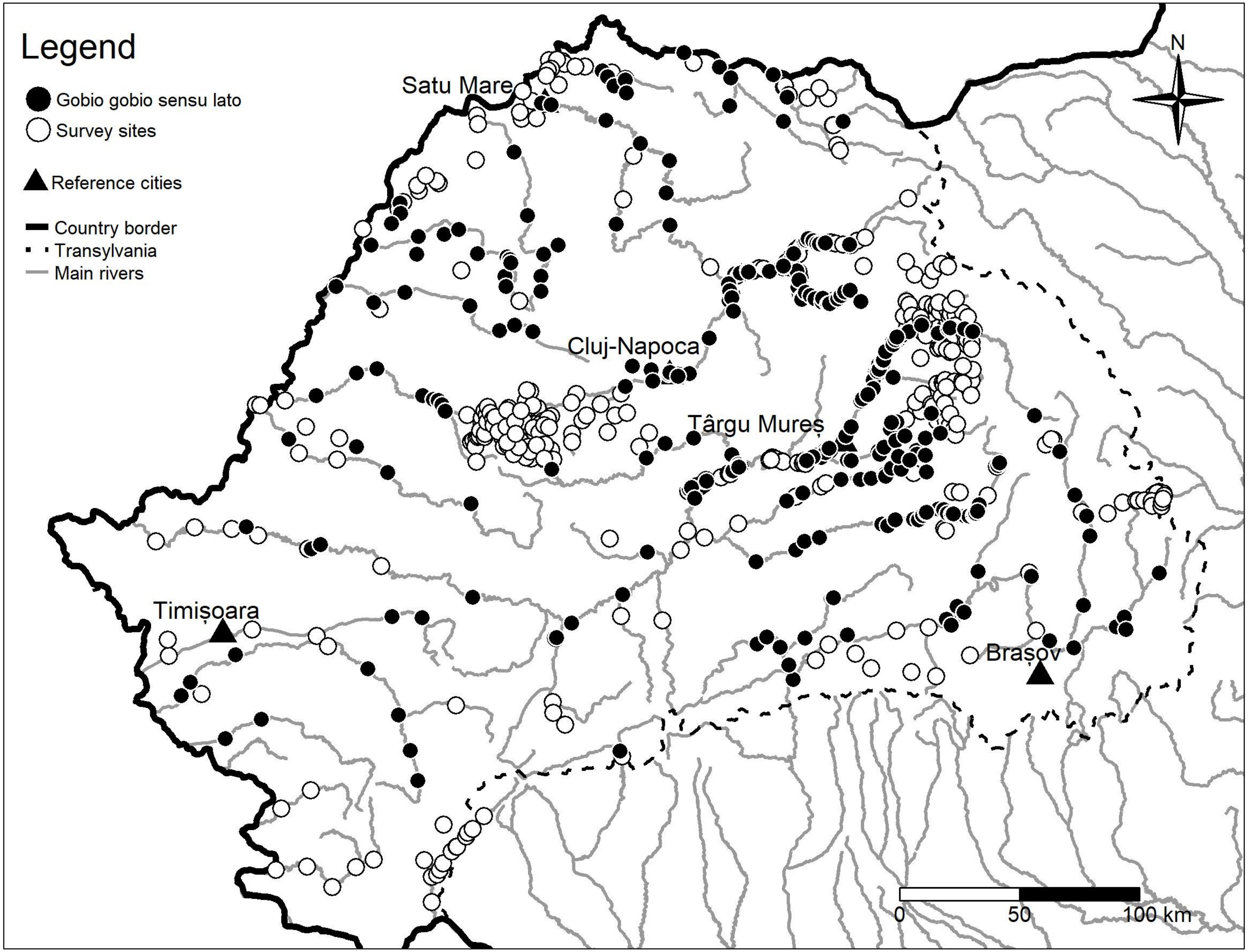

Map S31. Distribution of *Gobio gobio sensu lato*

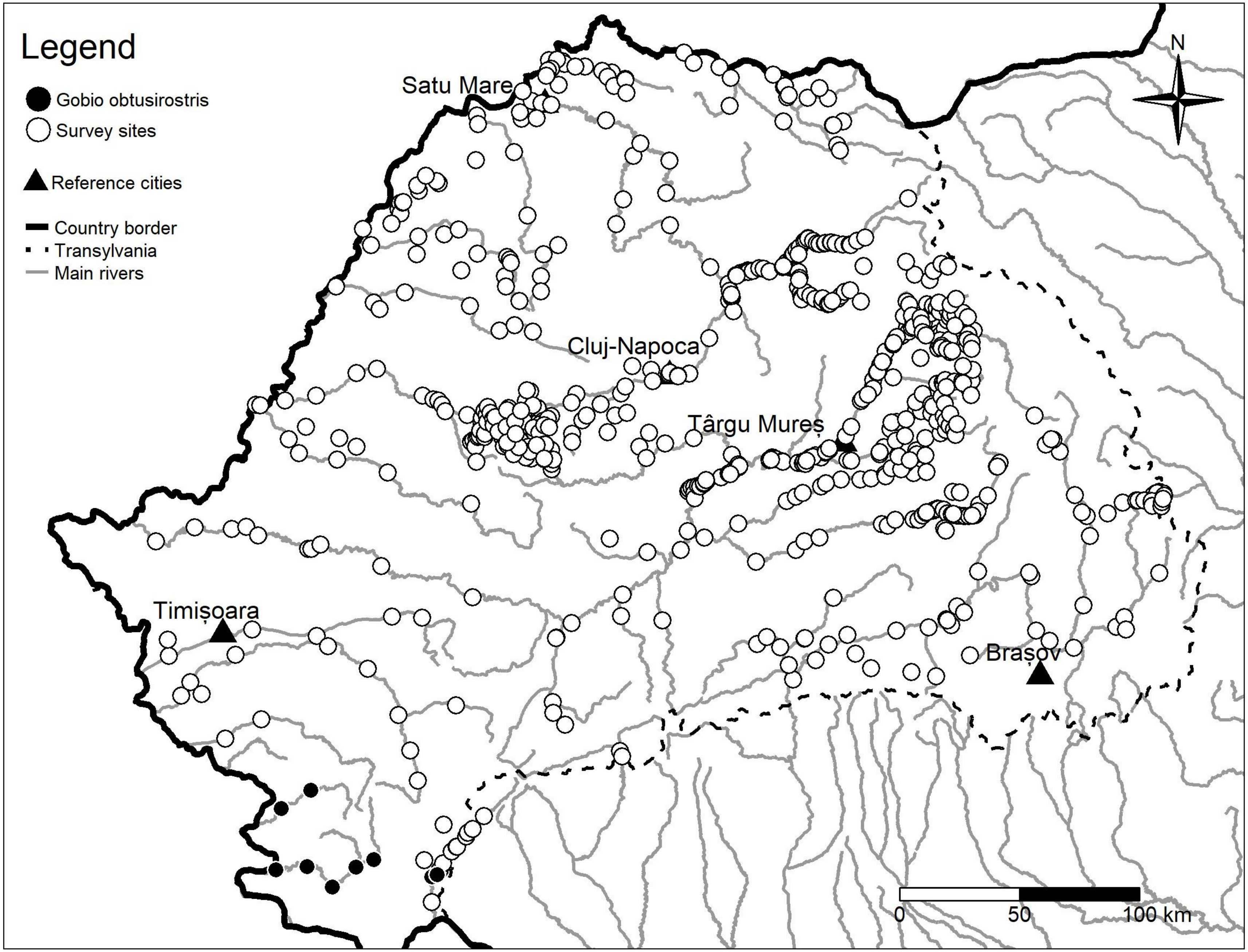

Map S32. Distribution of *Gobio obtusirostris*

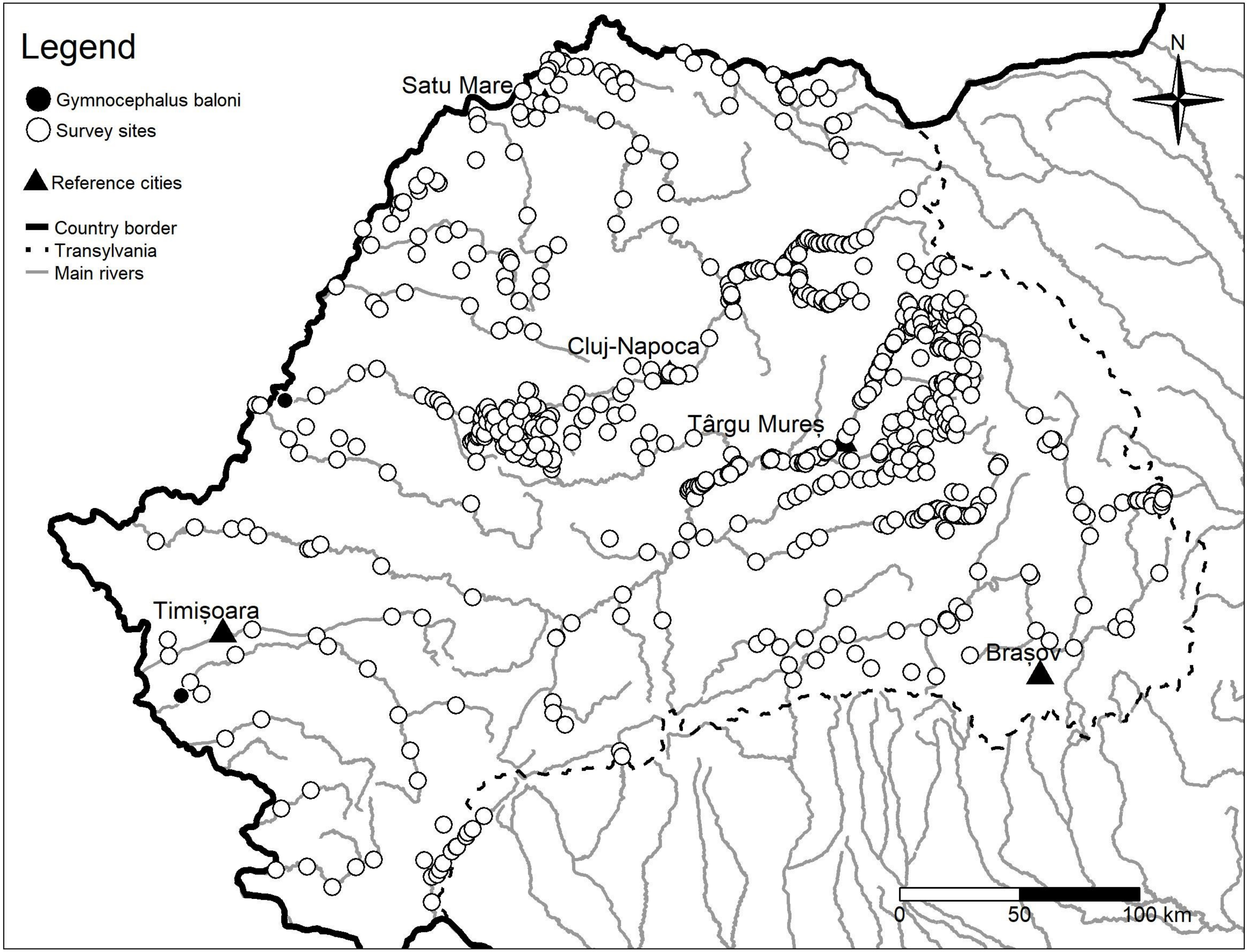

Map S33. Distribution of *Gymnocephalus baloni*

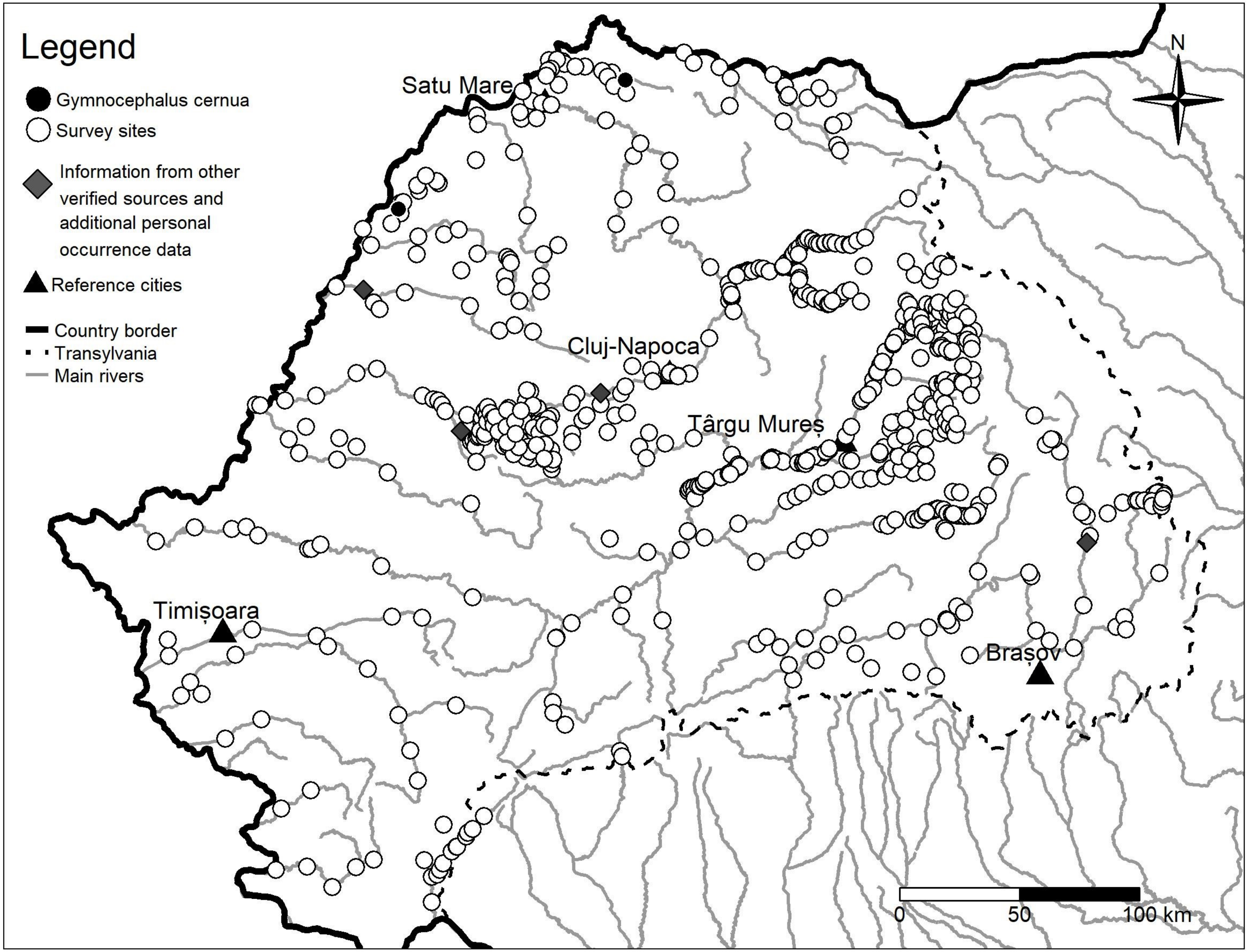

Map S34. Distribution of *Gymnocephalus cernua*

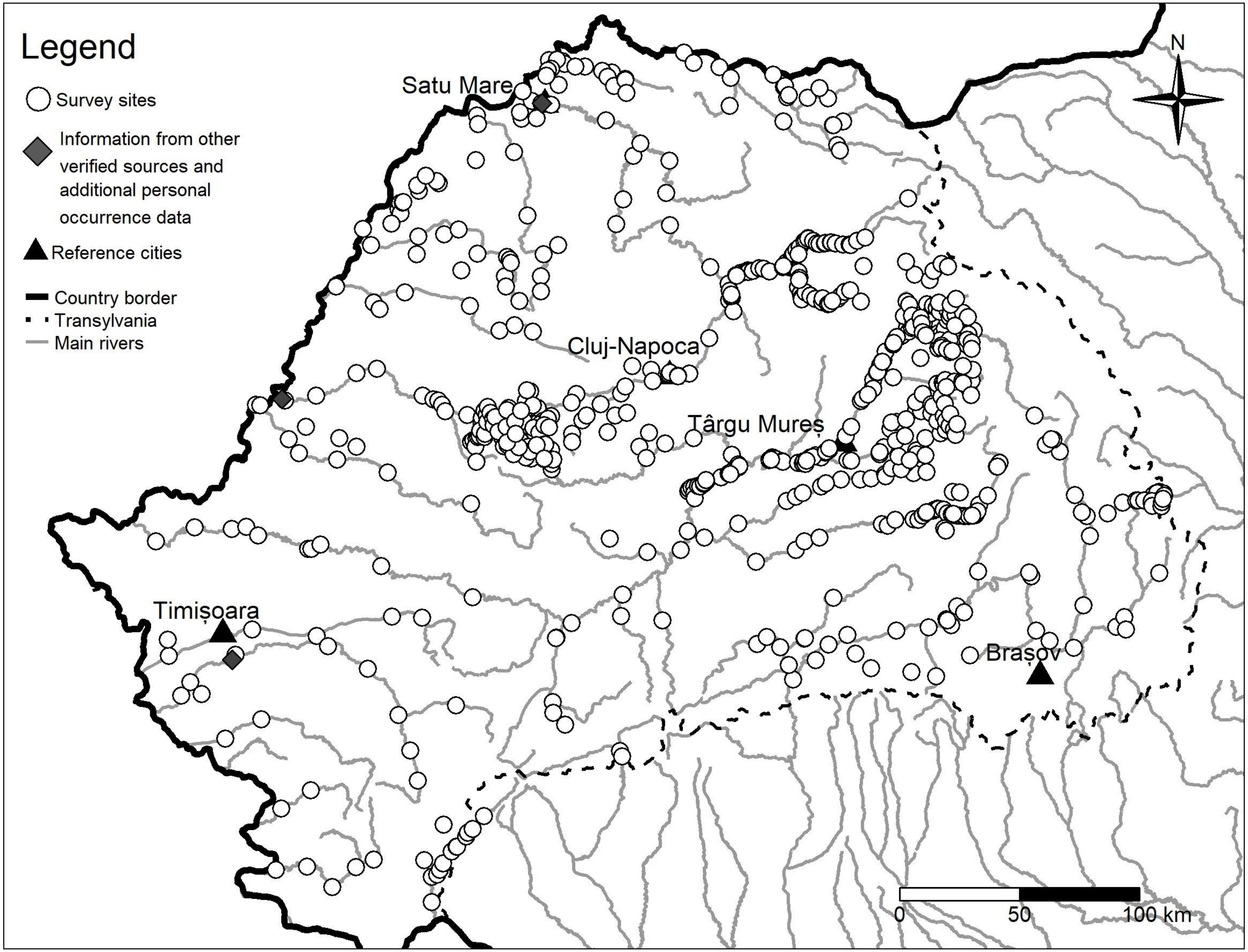

Map S35. Distribution of *Gymnocephalus schraetser*

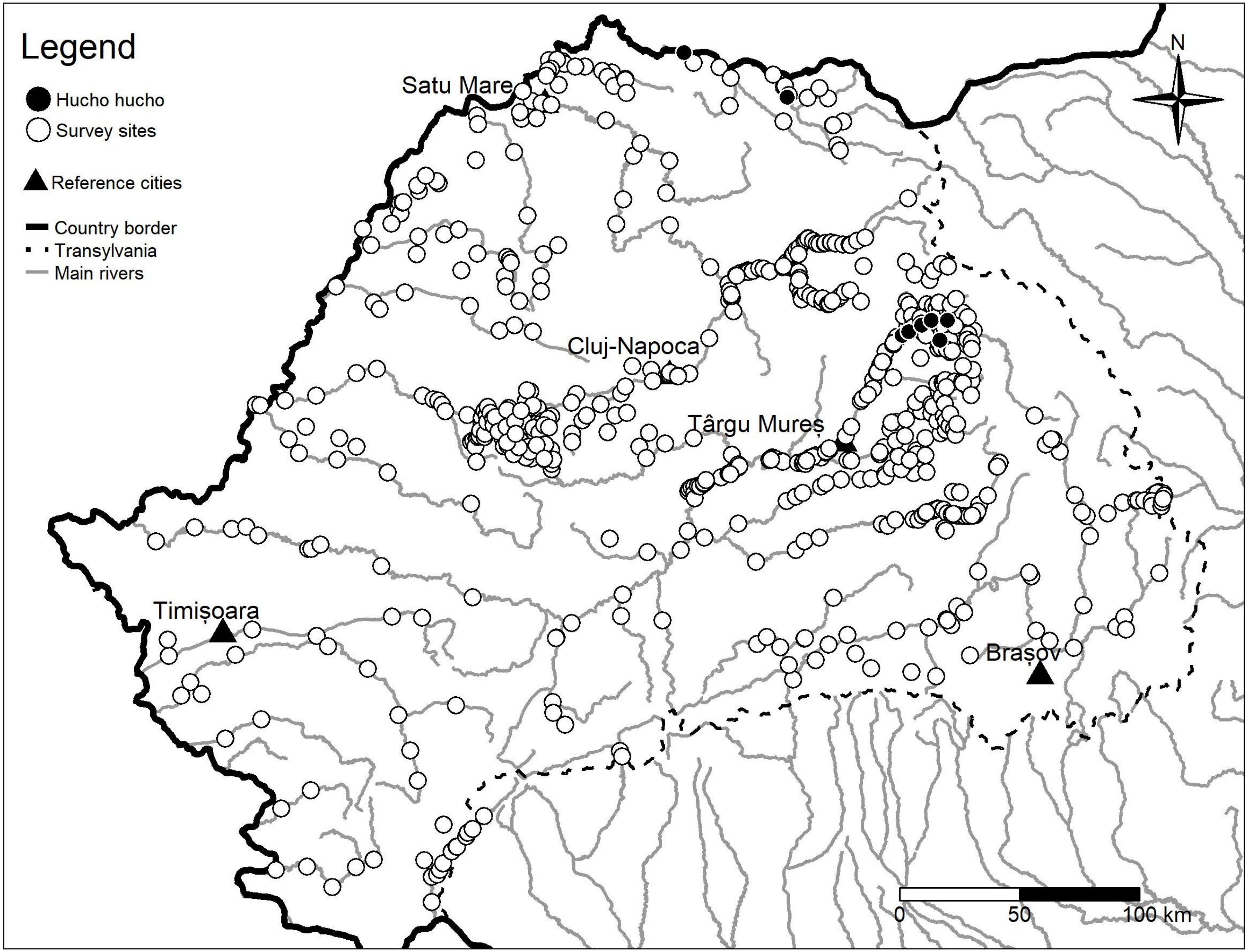

Map S36. Distribution of *Hucho hucho*

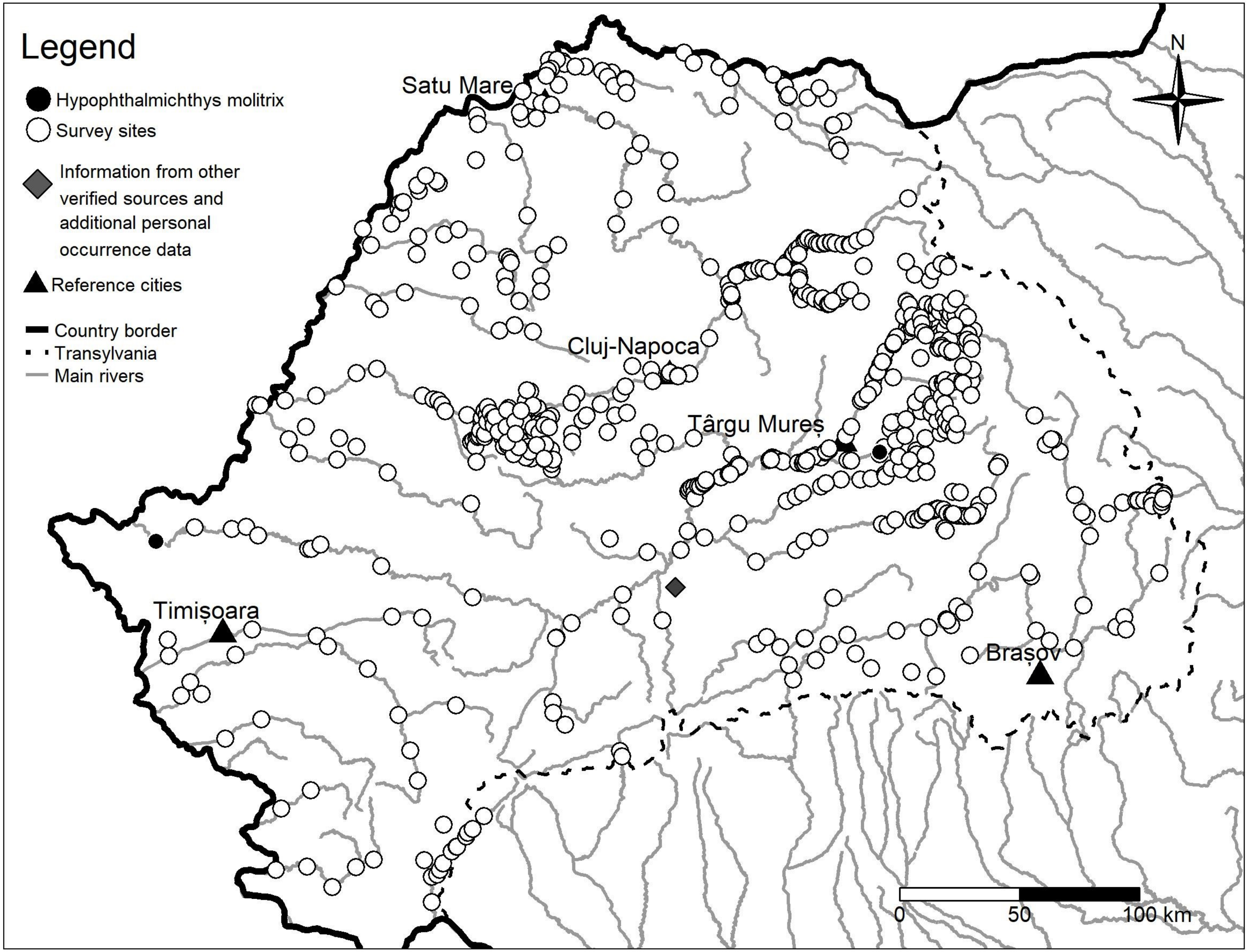

Map S37. Distribution of *Hypophthalmichthys molitrix*

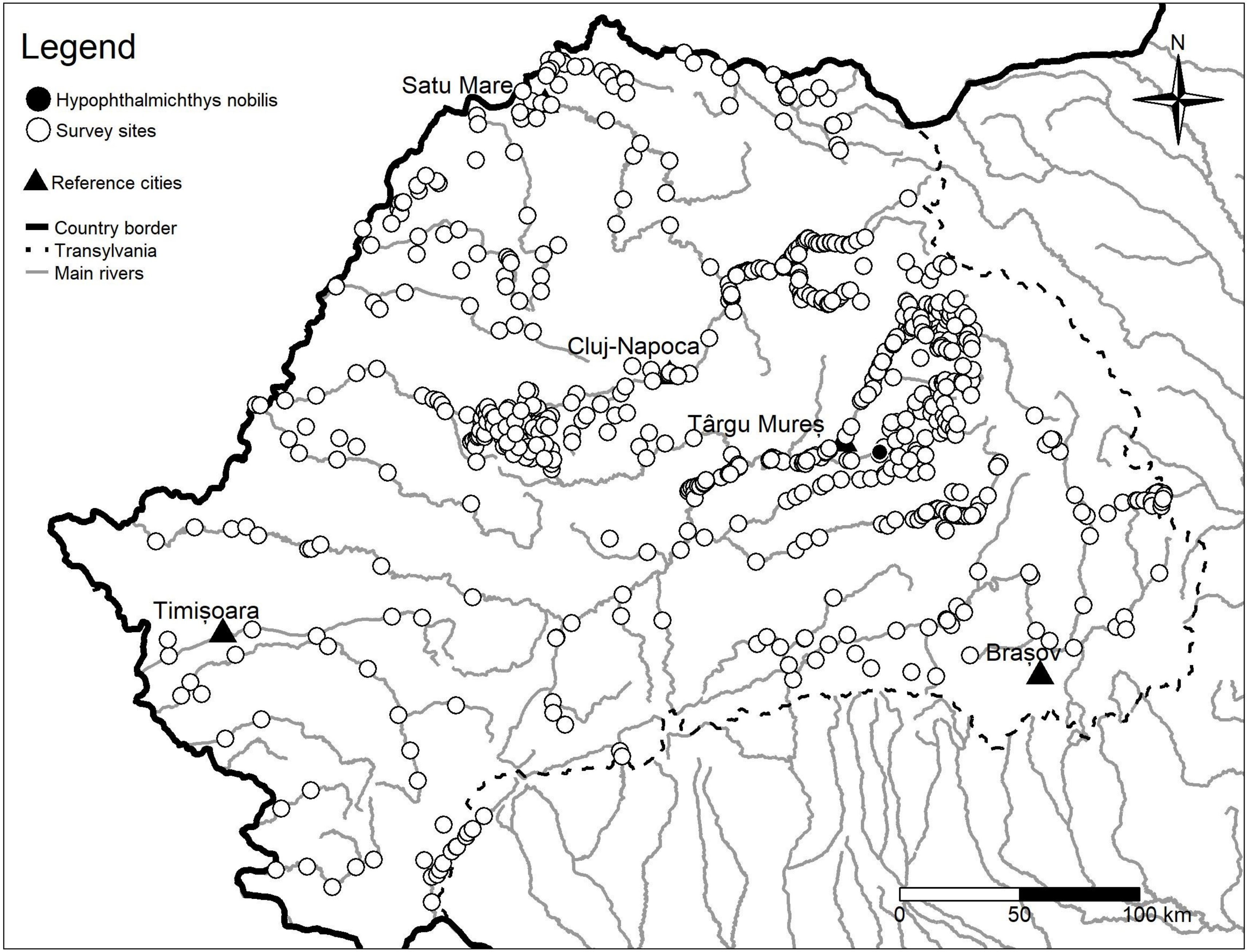

Map S38. Distribution of *Hypophthalmichthys nobilis*

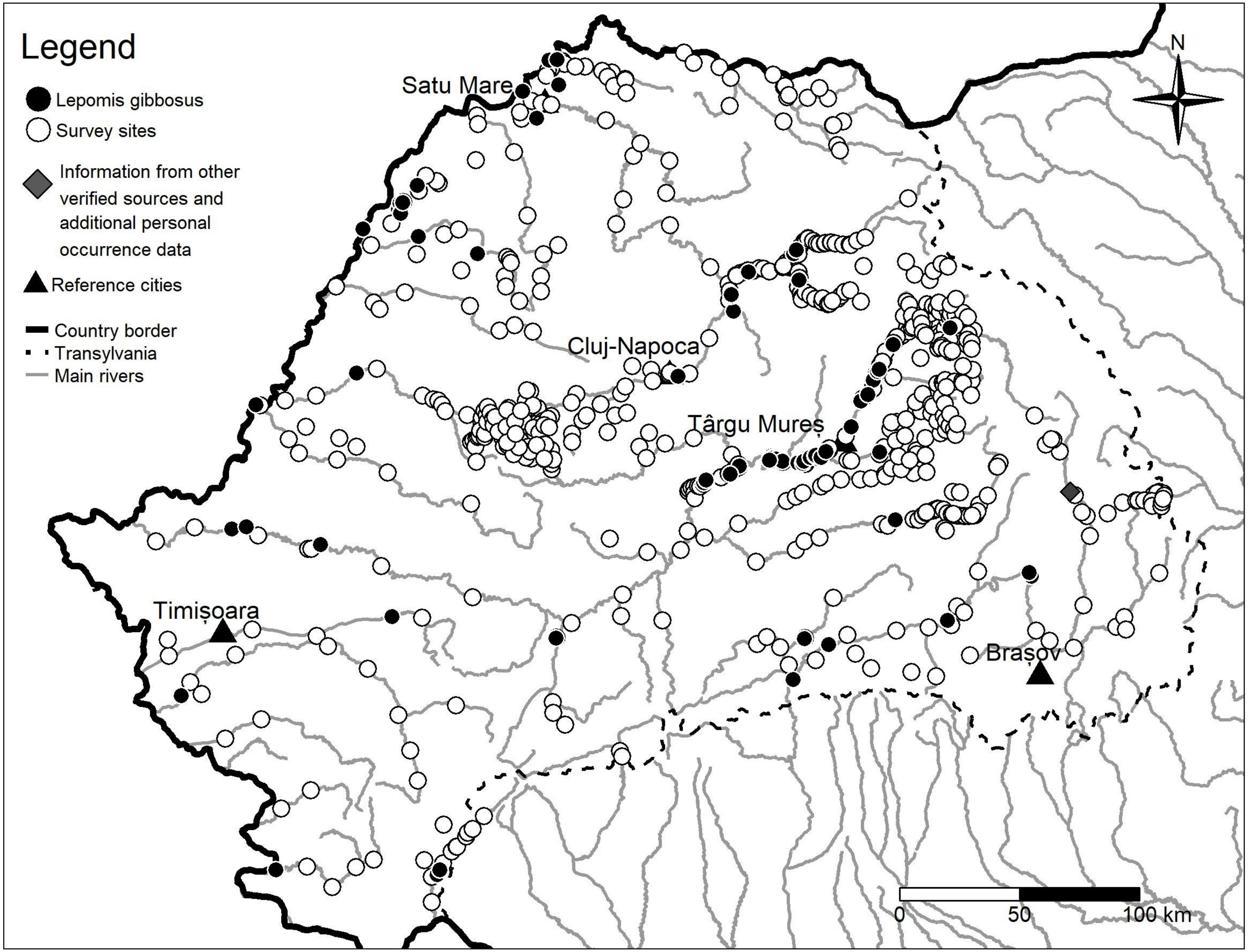

Map S39. Distribution of *Lepomis gibbosus*

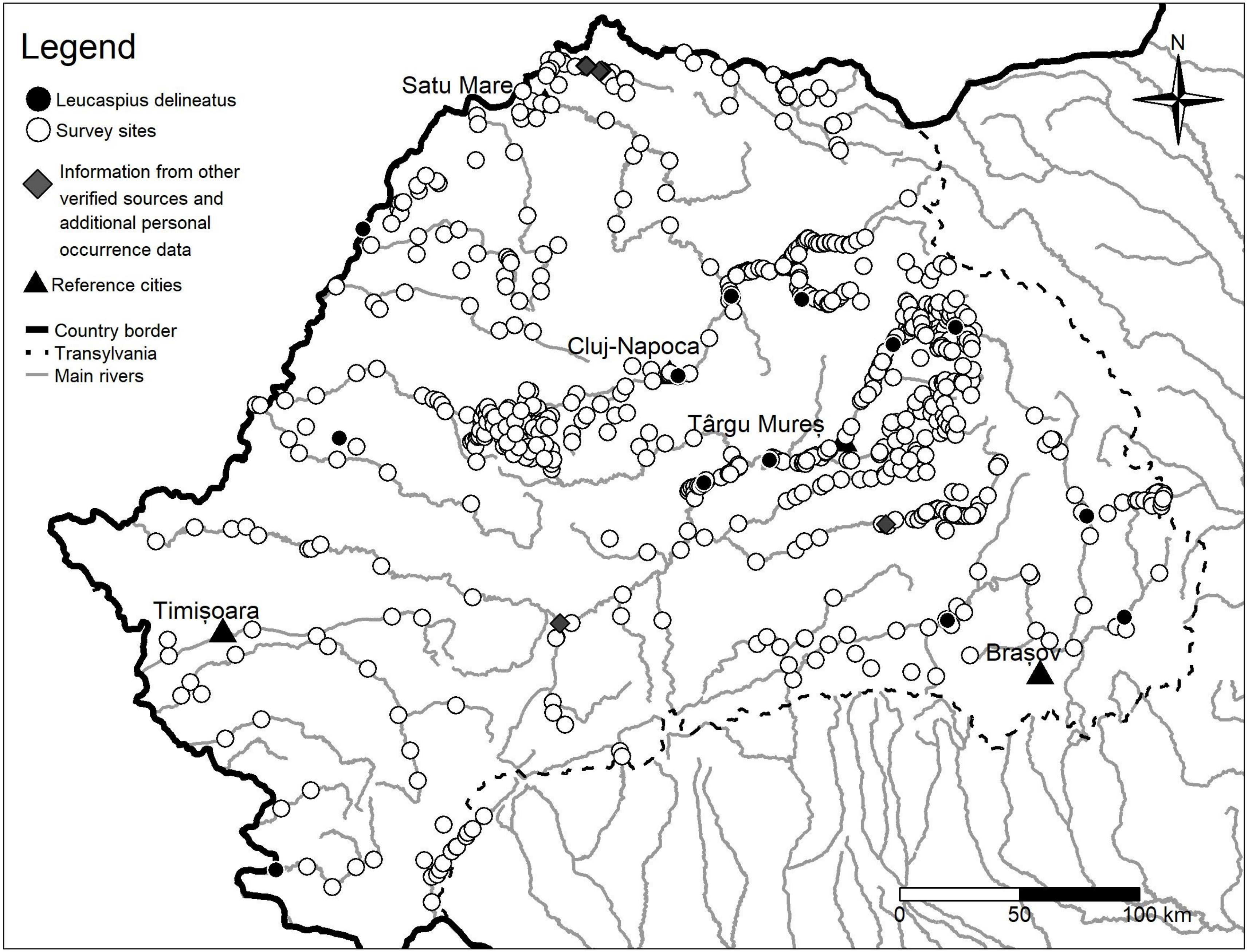

Map S40. Distribution of *Leucaspius delineatus*

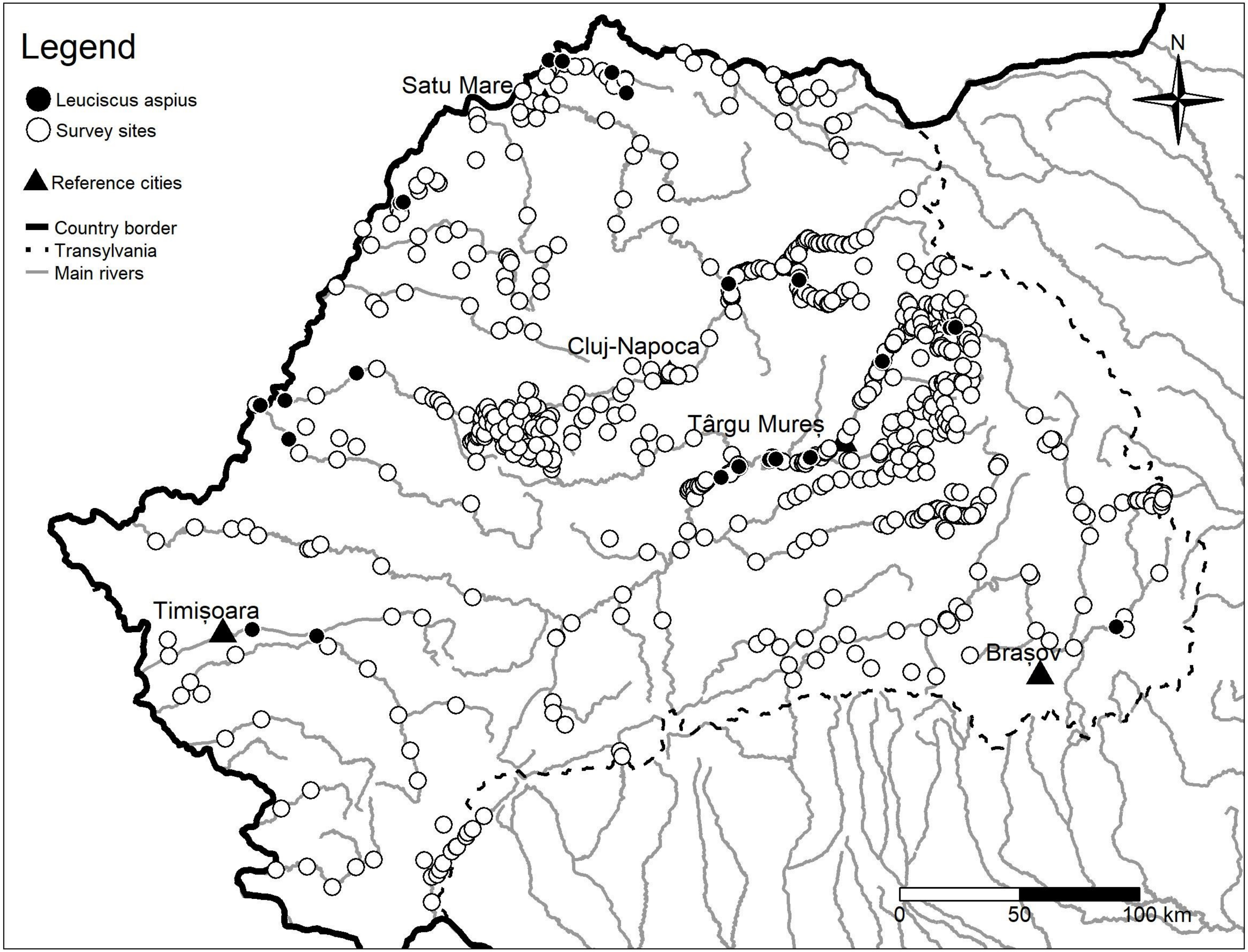

Map S41. Distribution of *Leuciscus aspiusra*

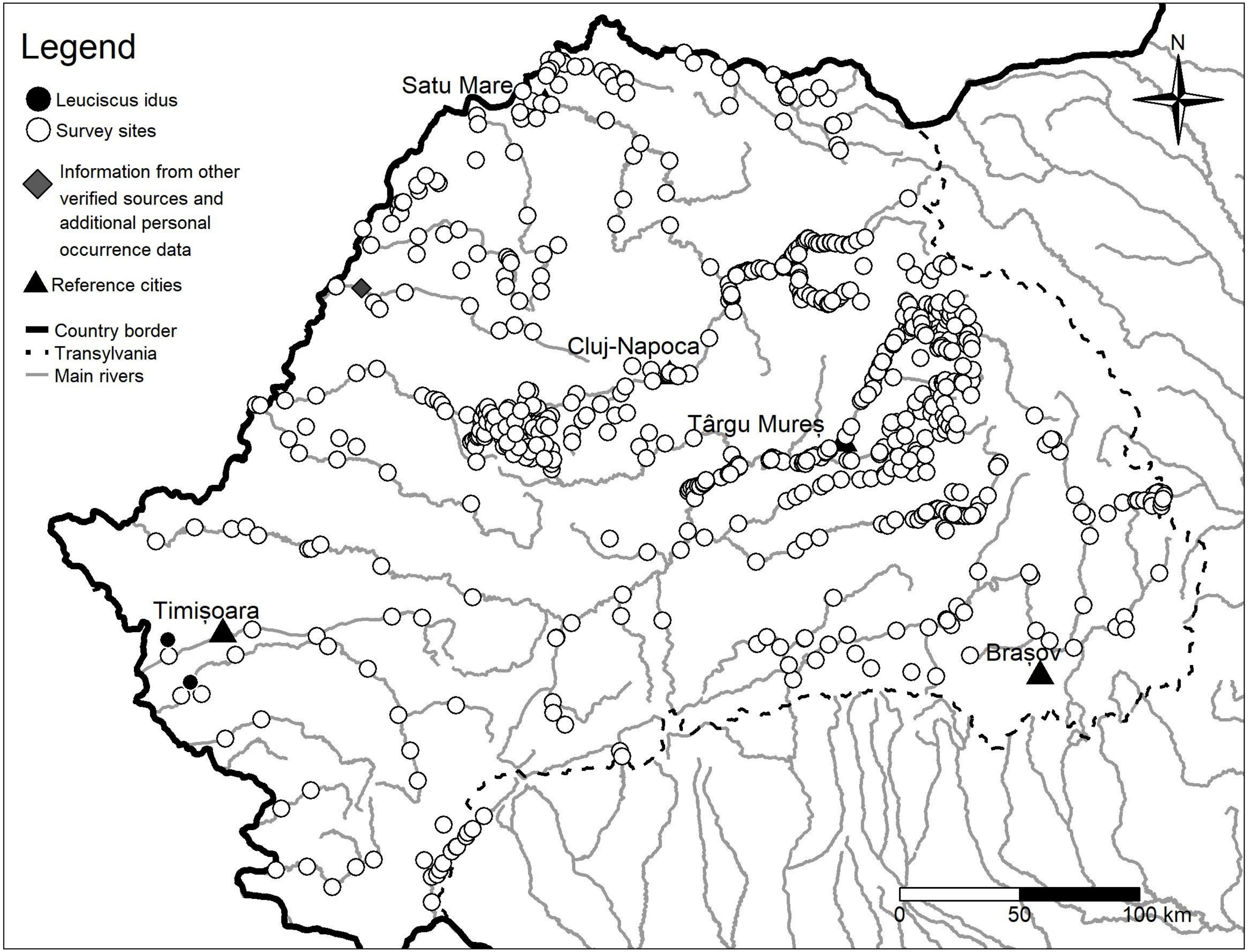

Map S42. Distribution of *Leuciscus idus*

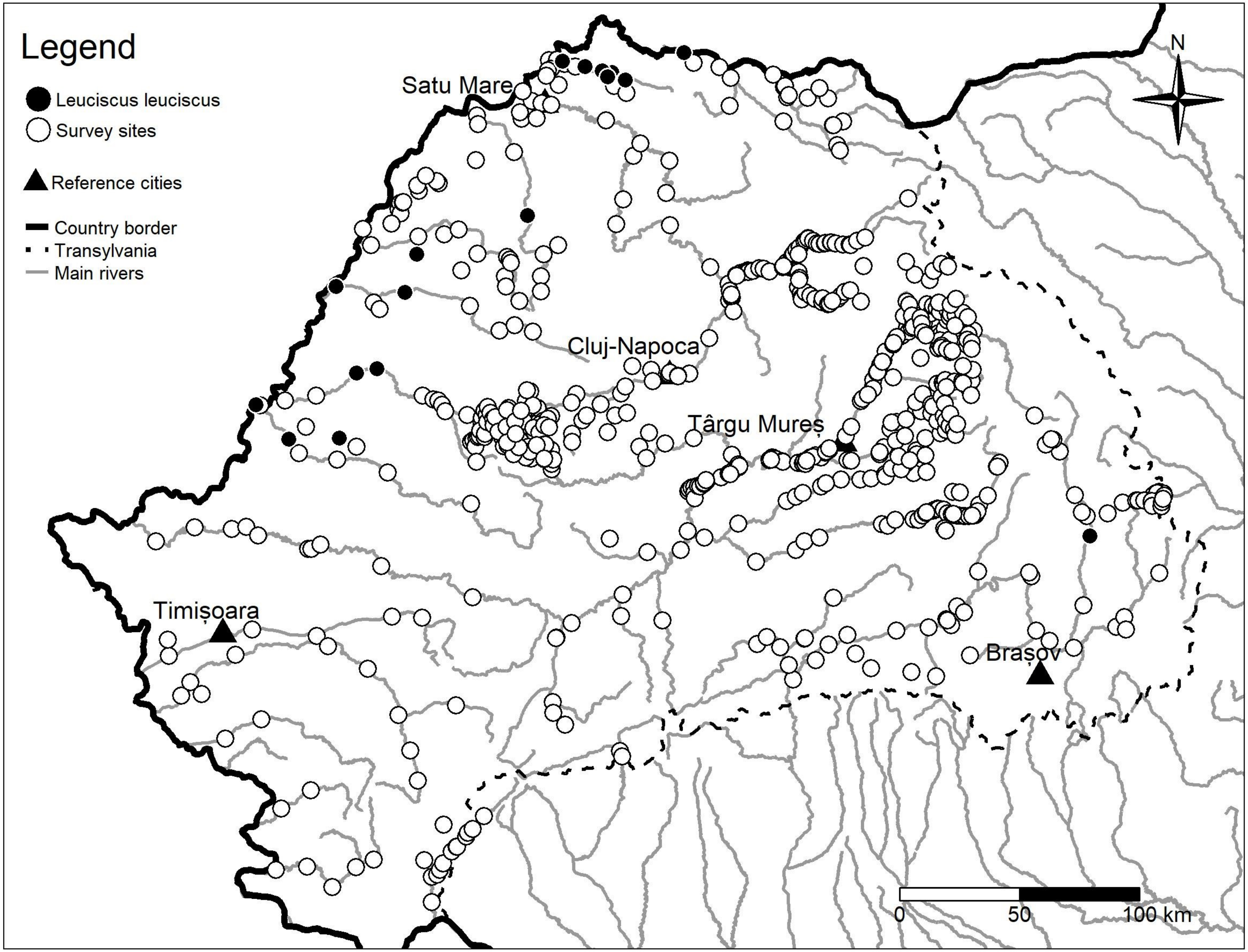

Map S43. Distribution of *Leuciscus leuciscus*

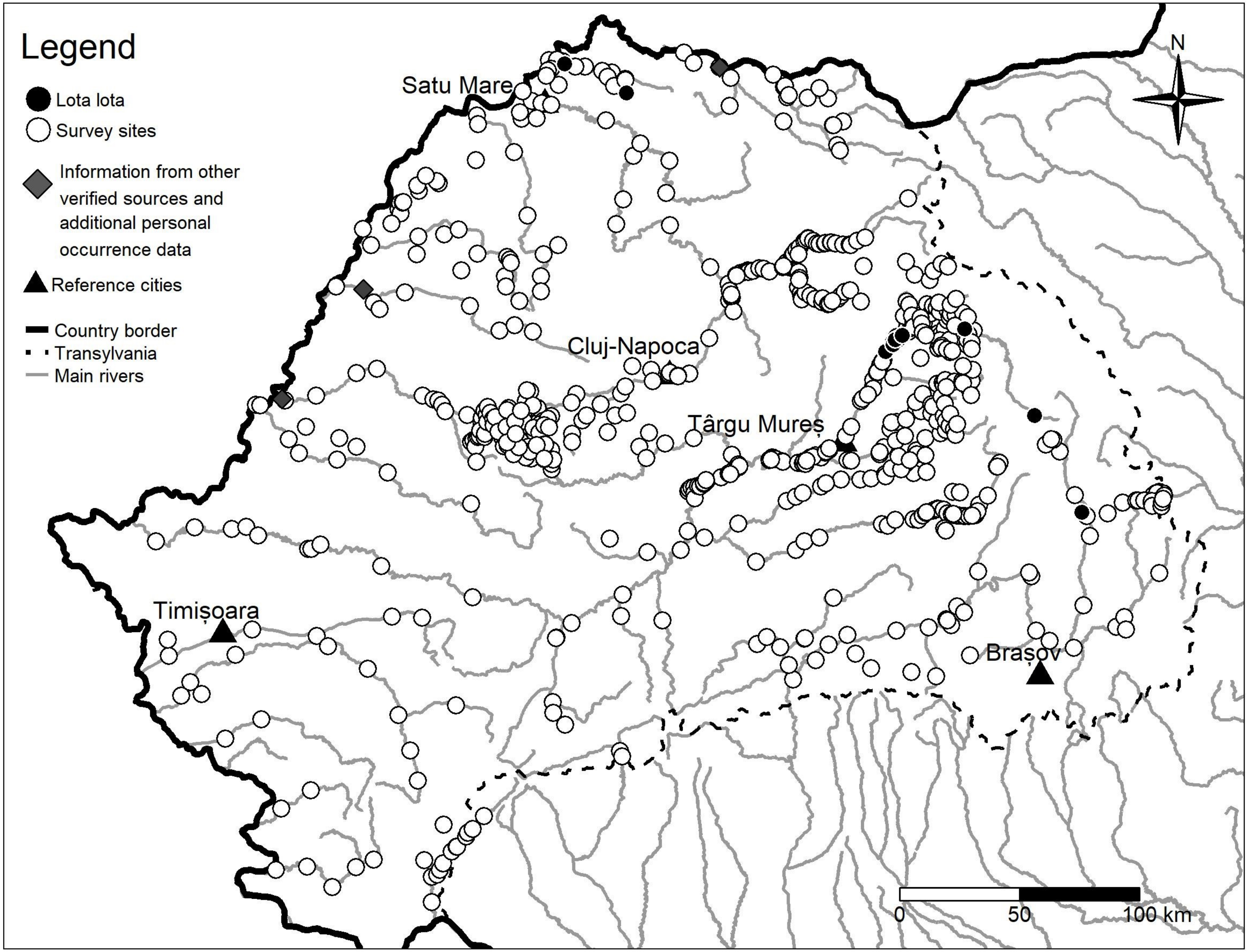

Map S44. Distribution of *Lota lota*

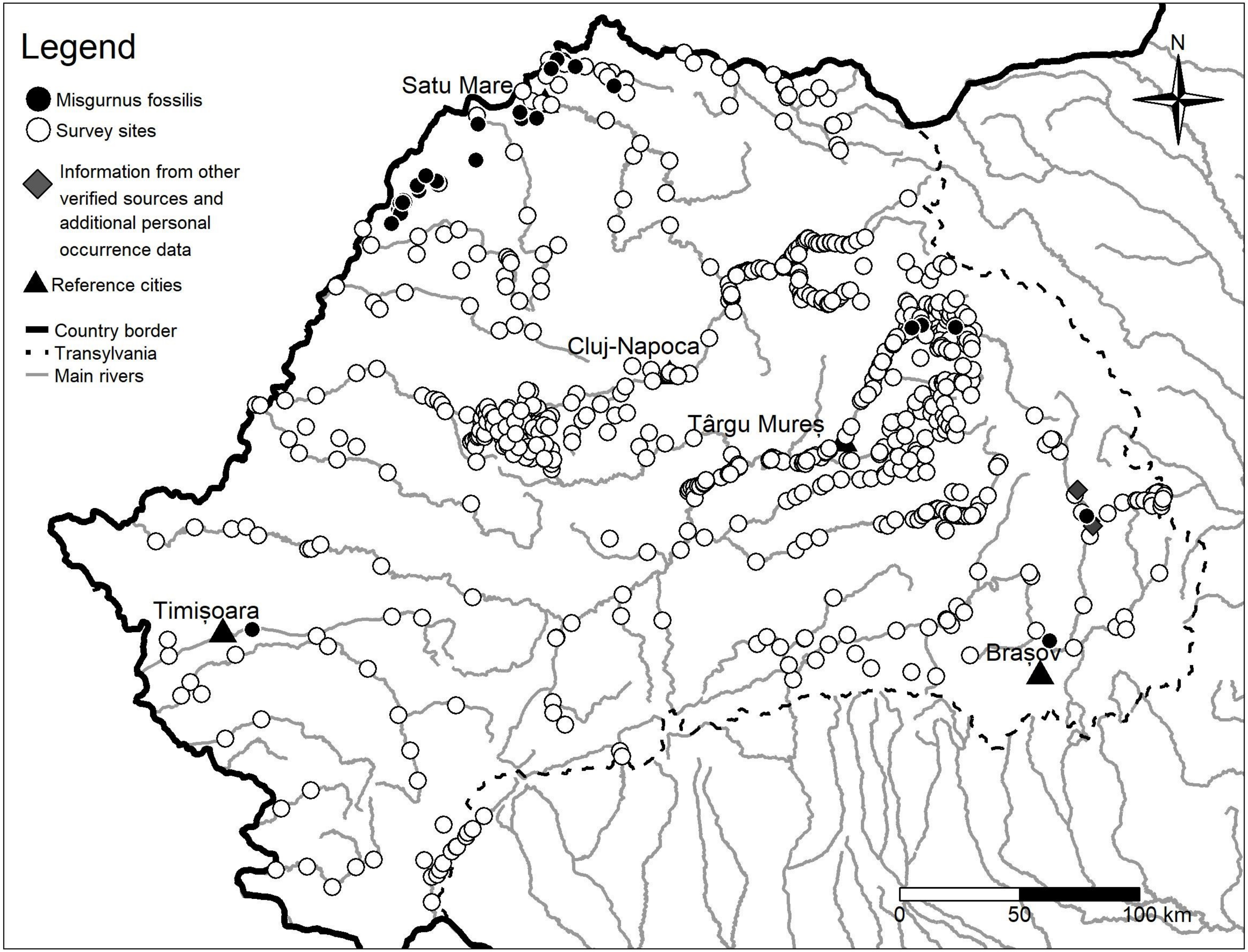

Map S45. Distribution of *Misgurnus fossilis*

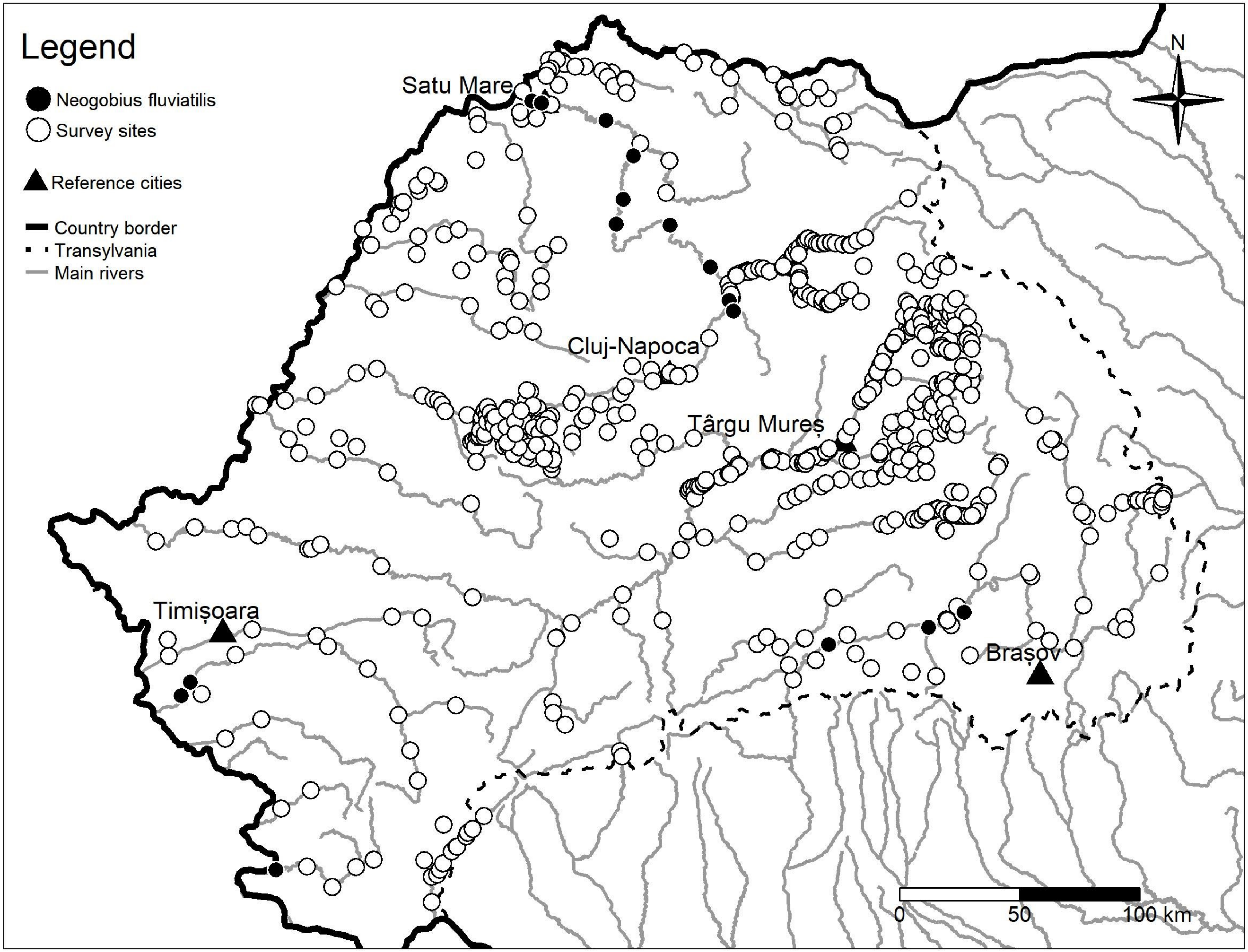

Map S46. Distribution of *Neogobius fluviatilis*

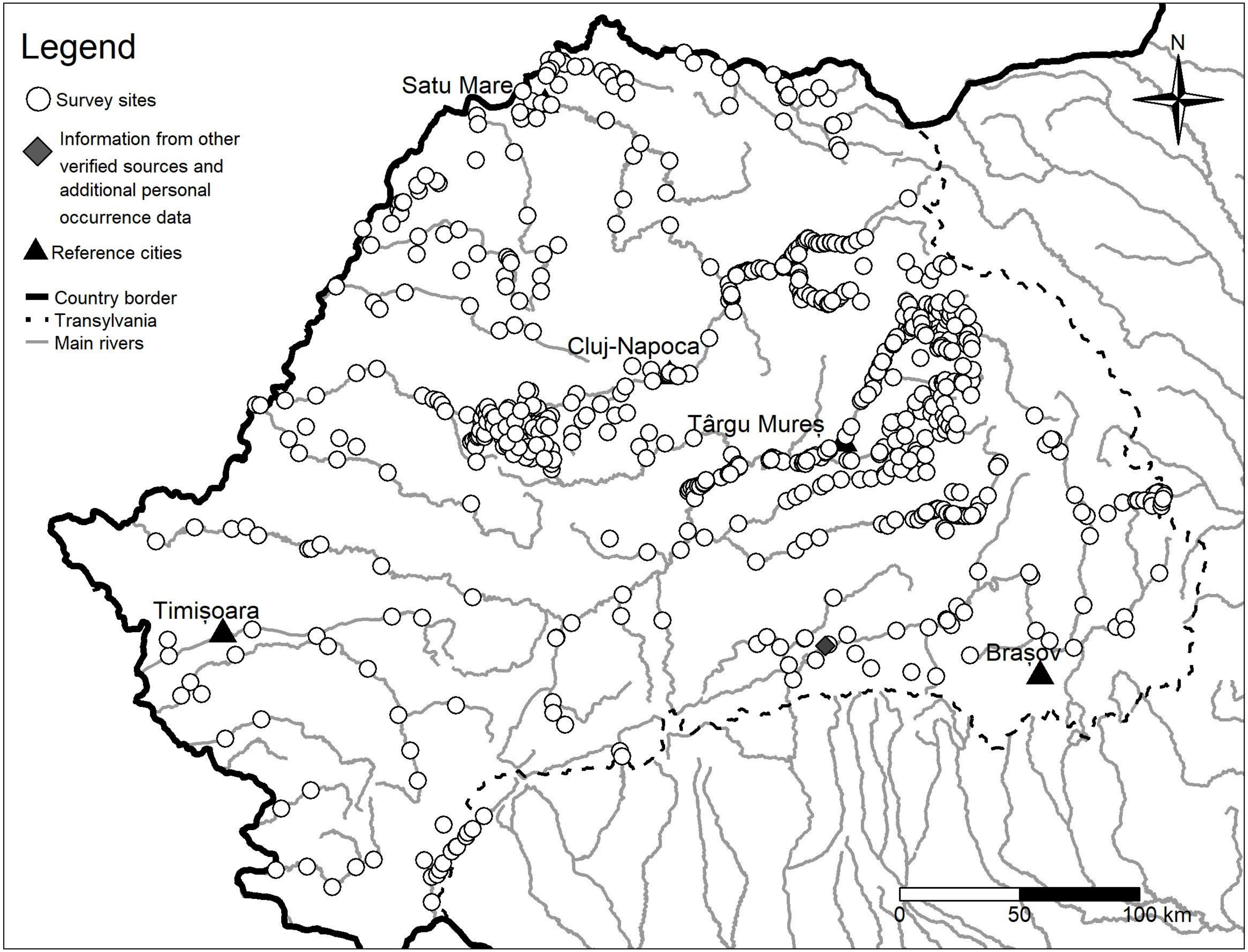

Map S47. Distribution of *Neogobius melanostomus*

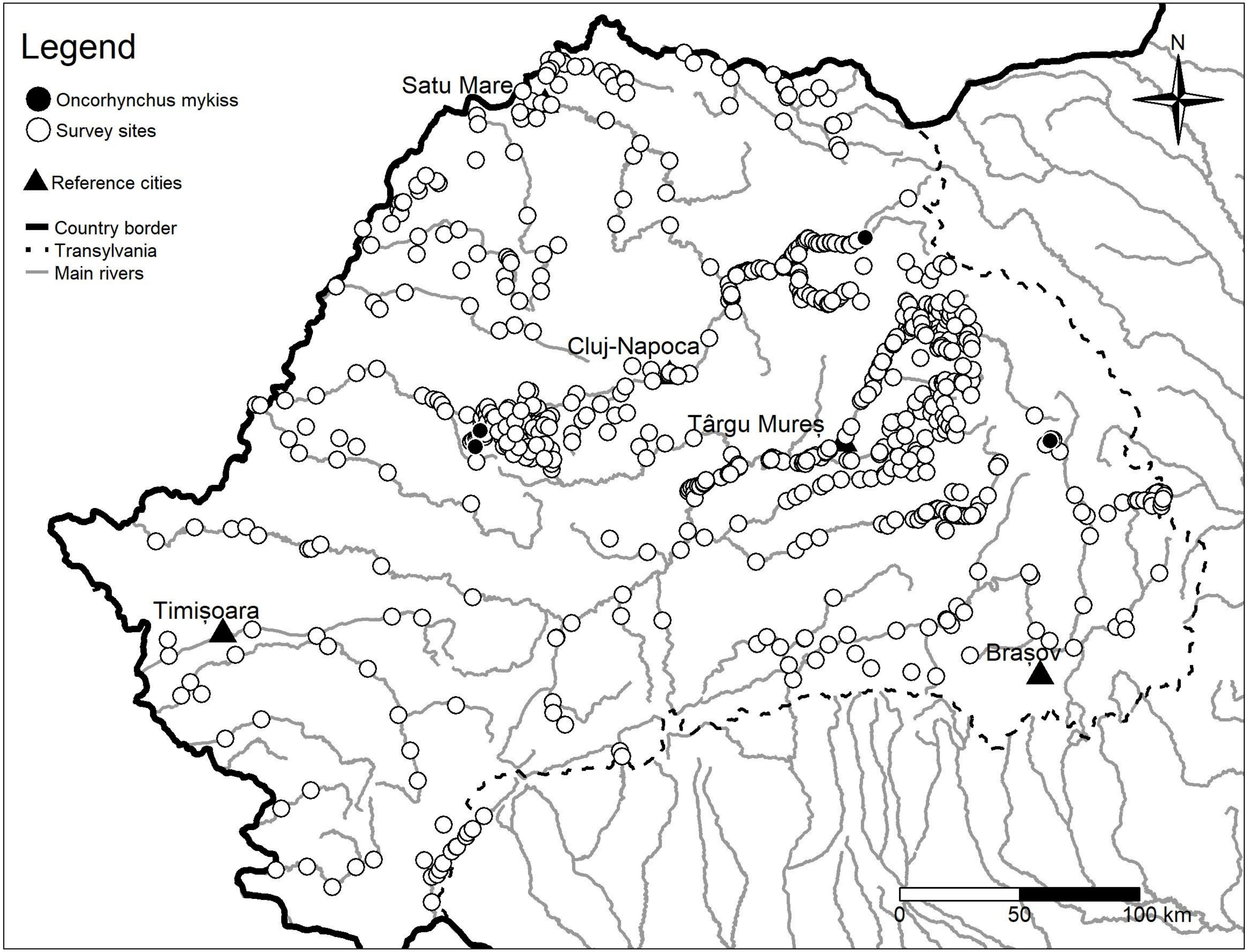

Map S48. Distribution of *Oncorhynchus mykiss*

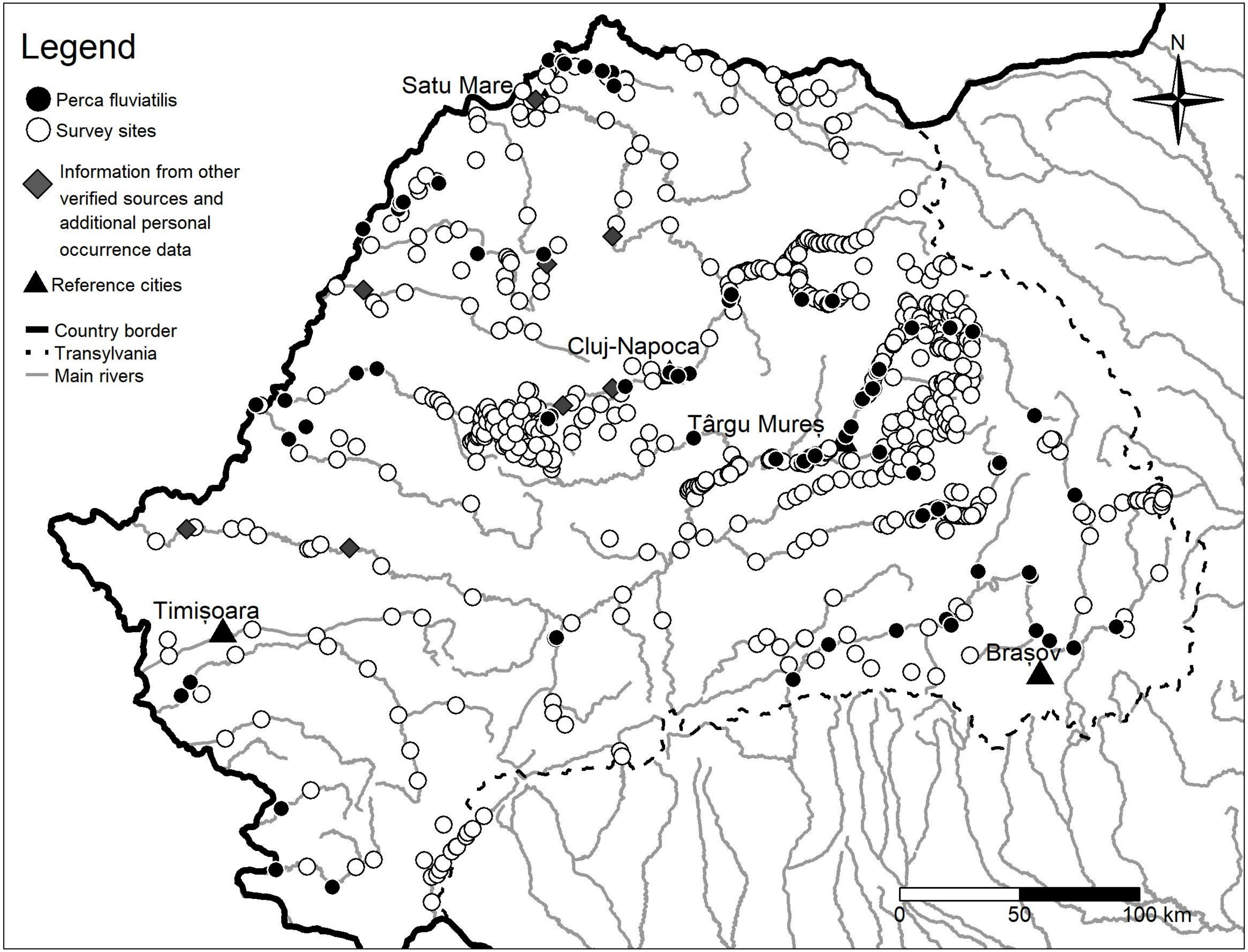

Map S49. Distribution of *Perca fluviatilis*

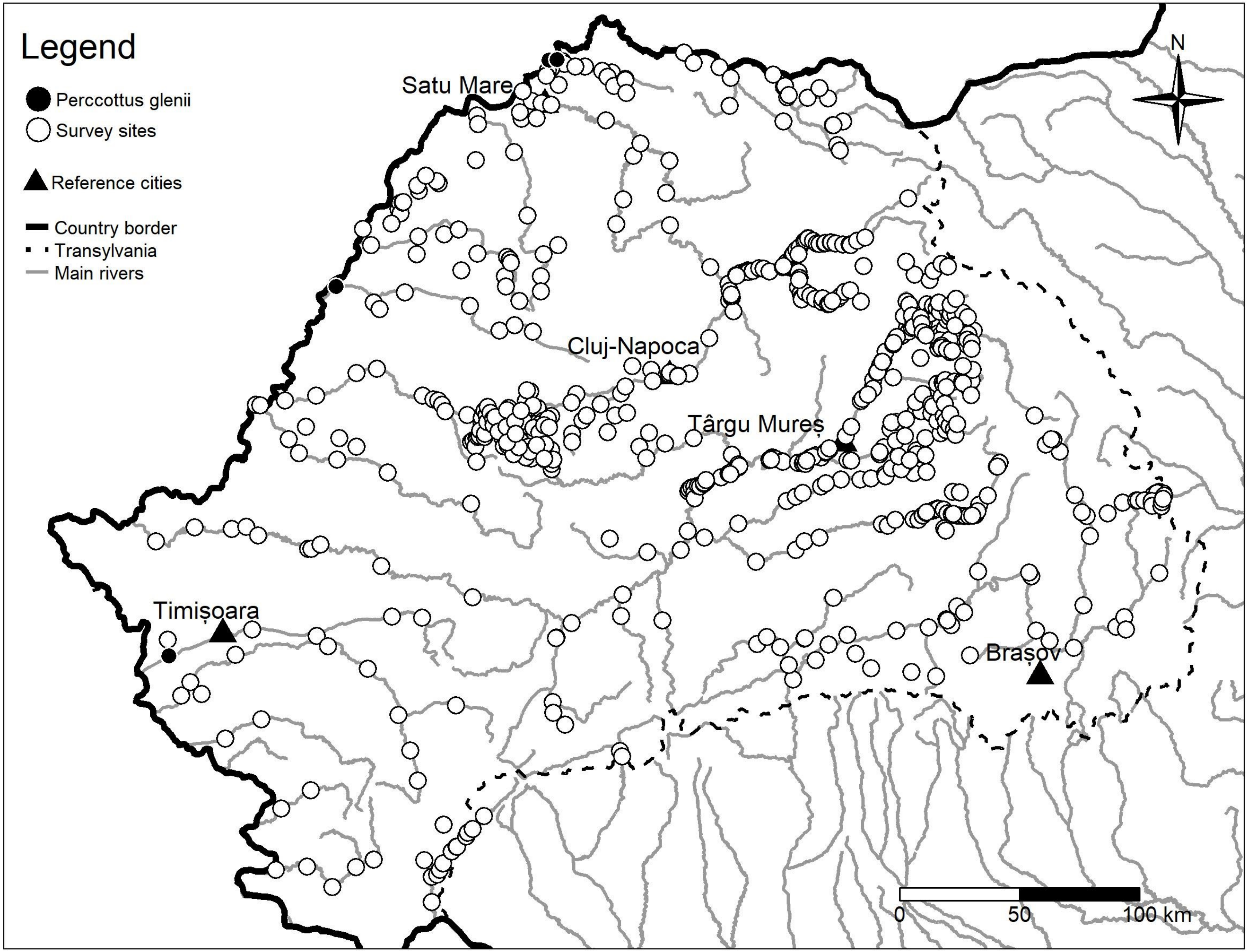

Map S50. Distribution of *Perccottus glenii*

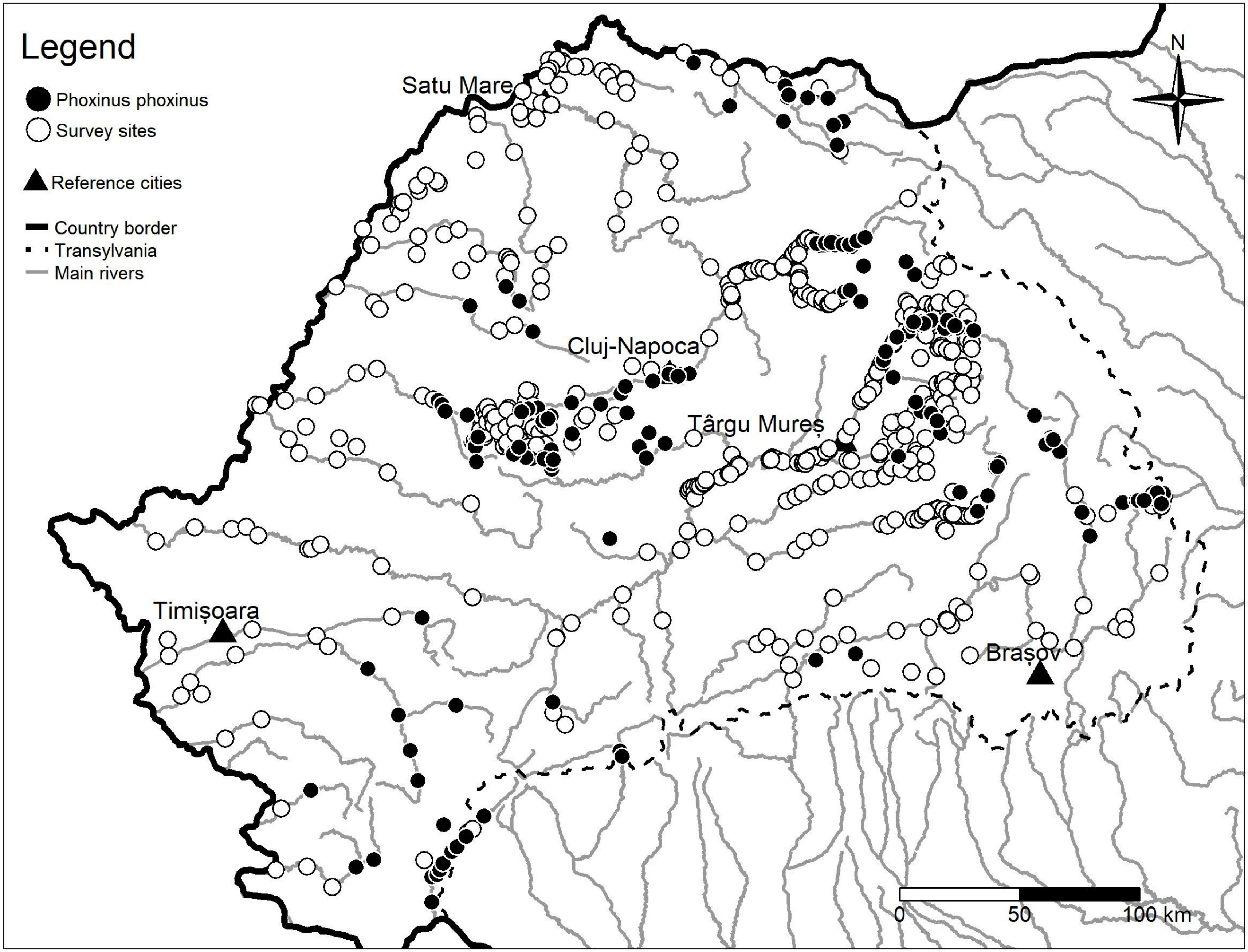

Map S51. Distribution of *Phoxinus phoxinus*

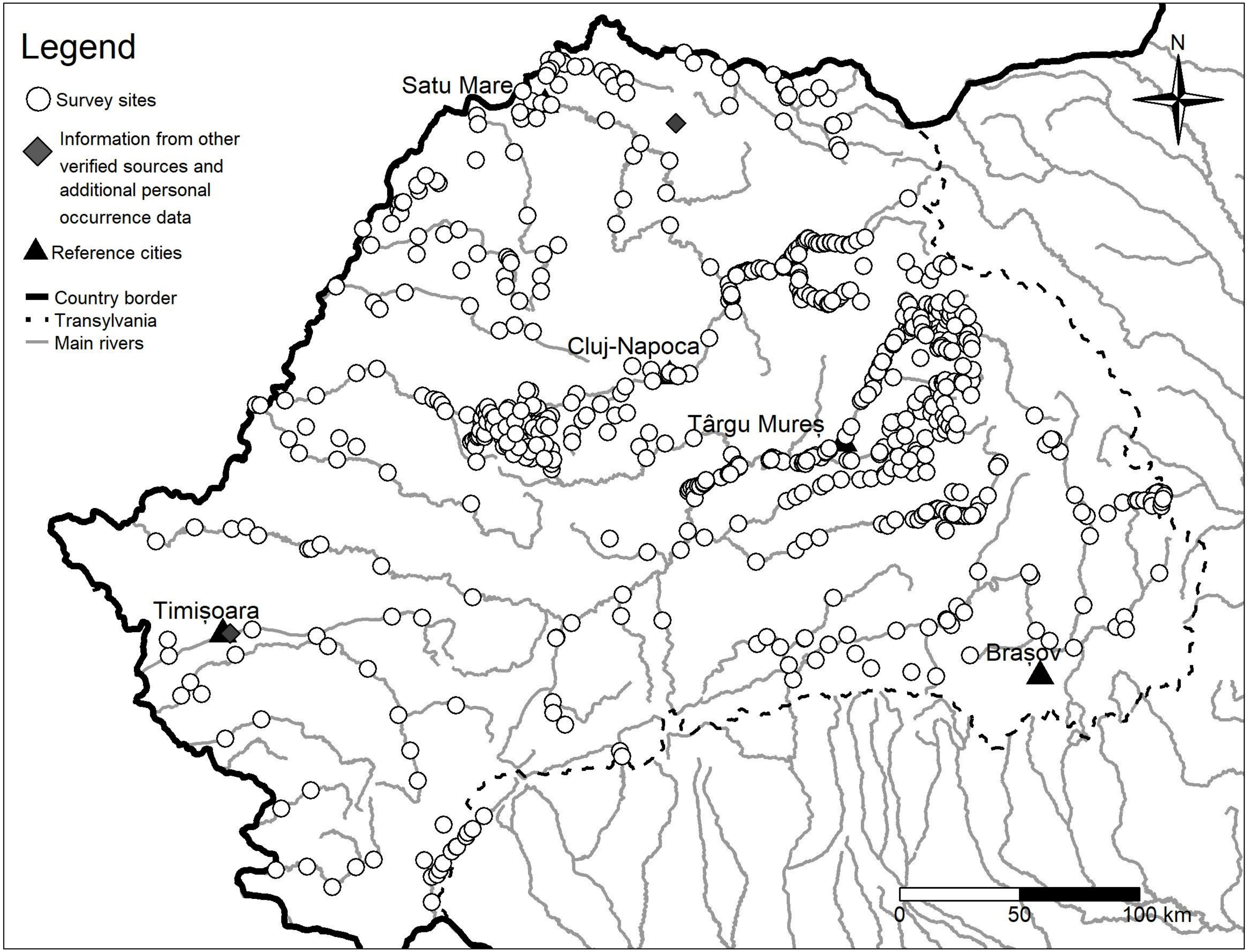

Map S52. Distribution of *Piaractus brachypomus*

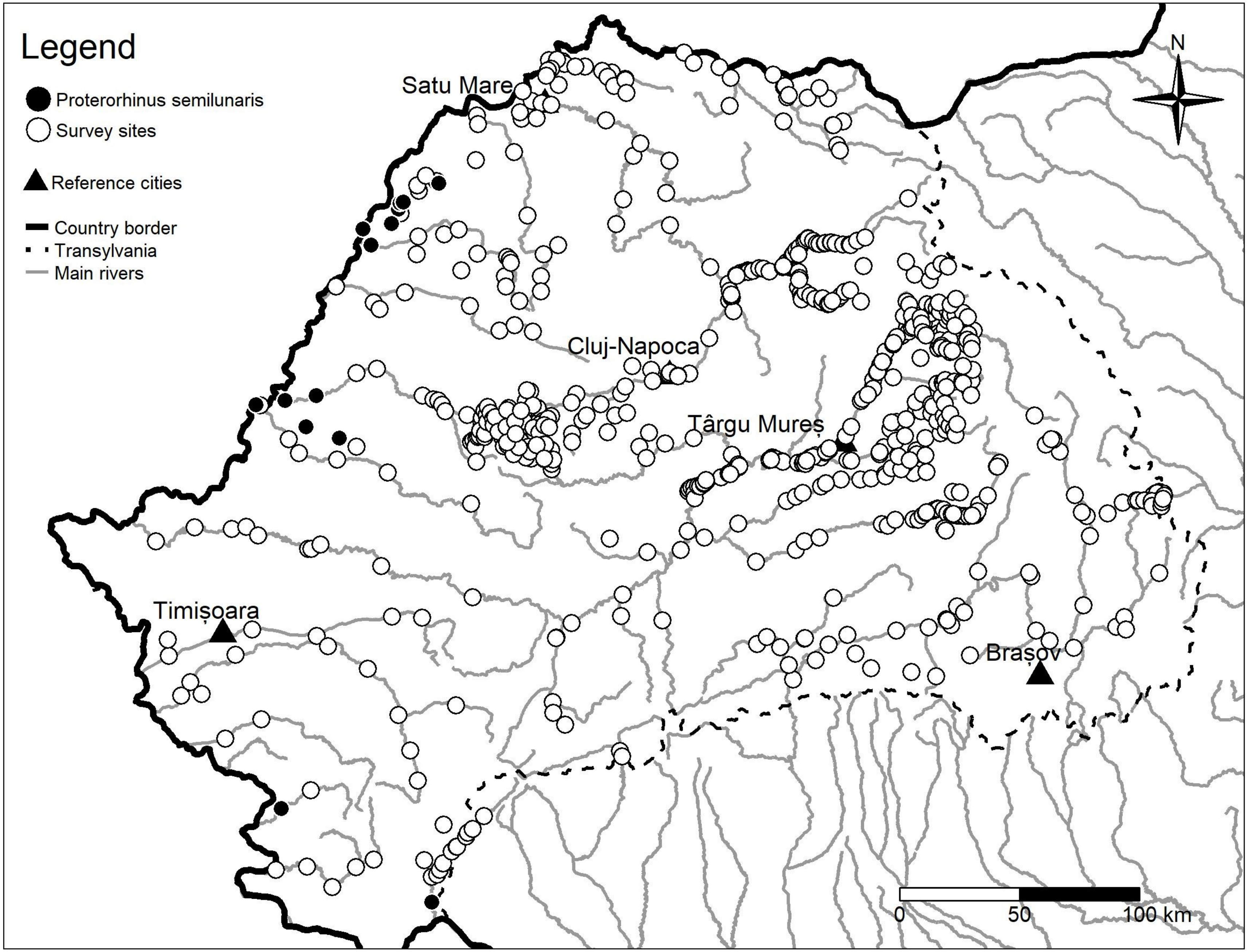

Map S53. Distribution of *Proterorhinus semilunaris*

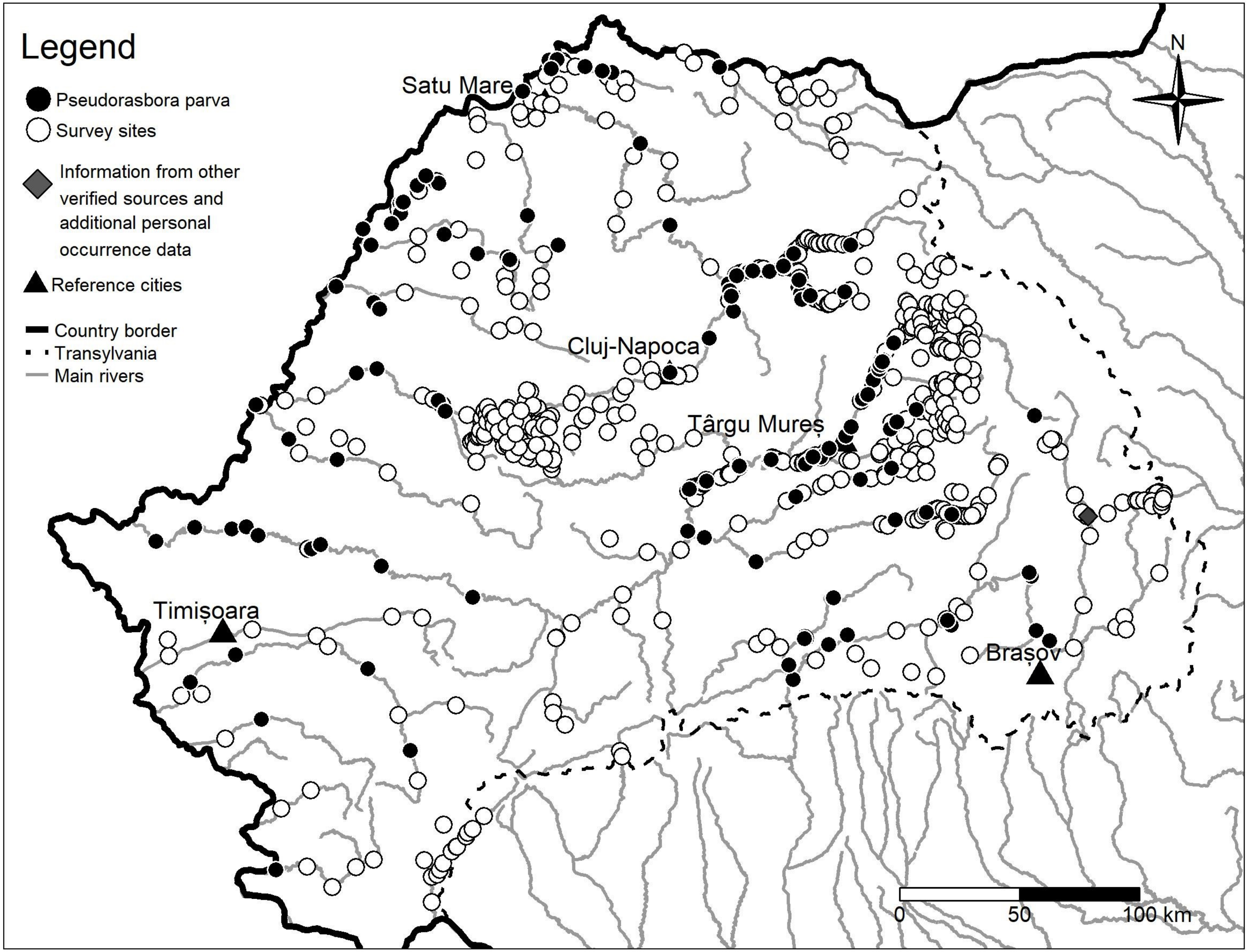

Map S54. Distribution of *Pseudorasbora parva*

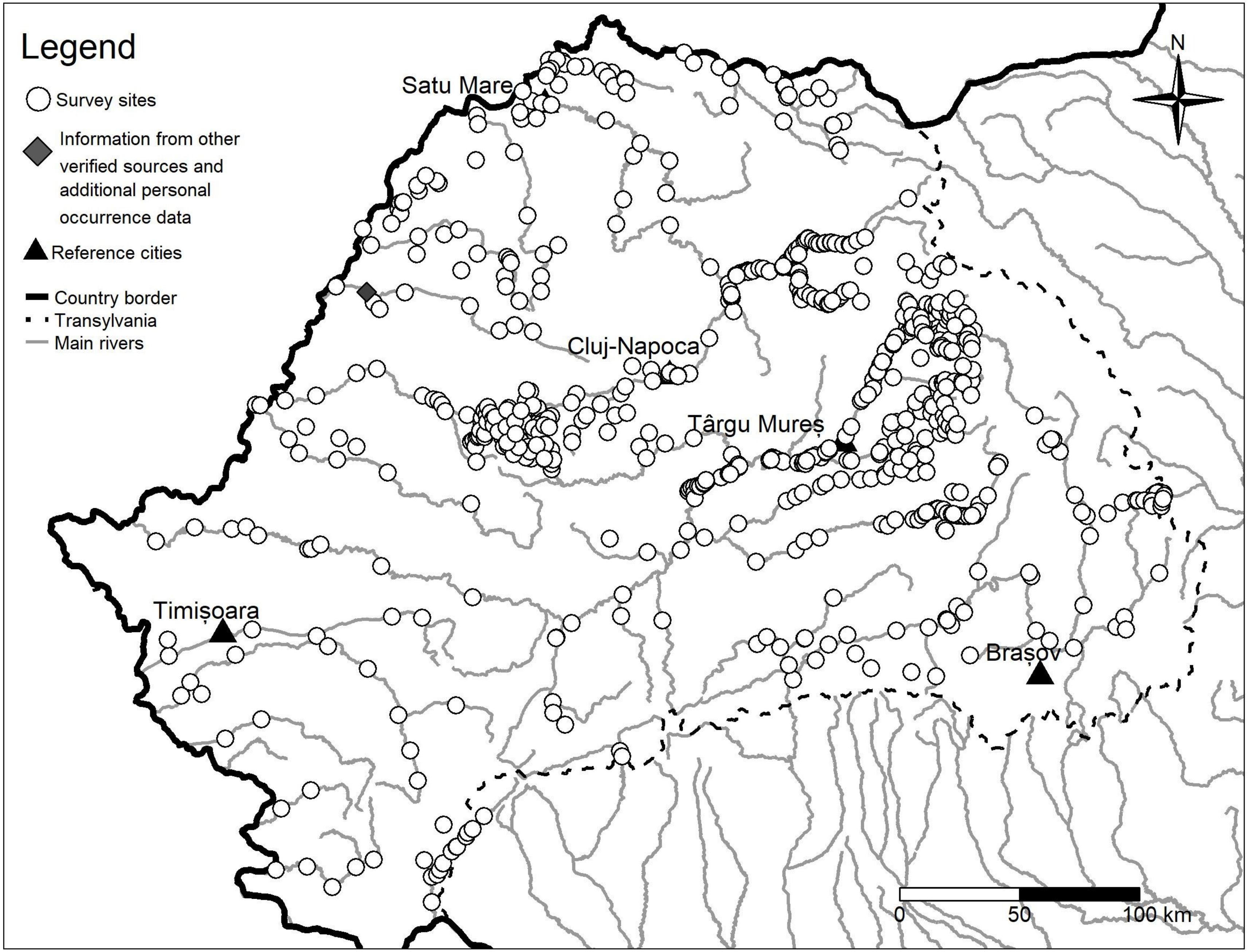

Map S55. Distribution of *Pygocentrus nattereri*

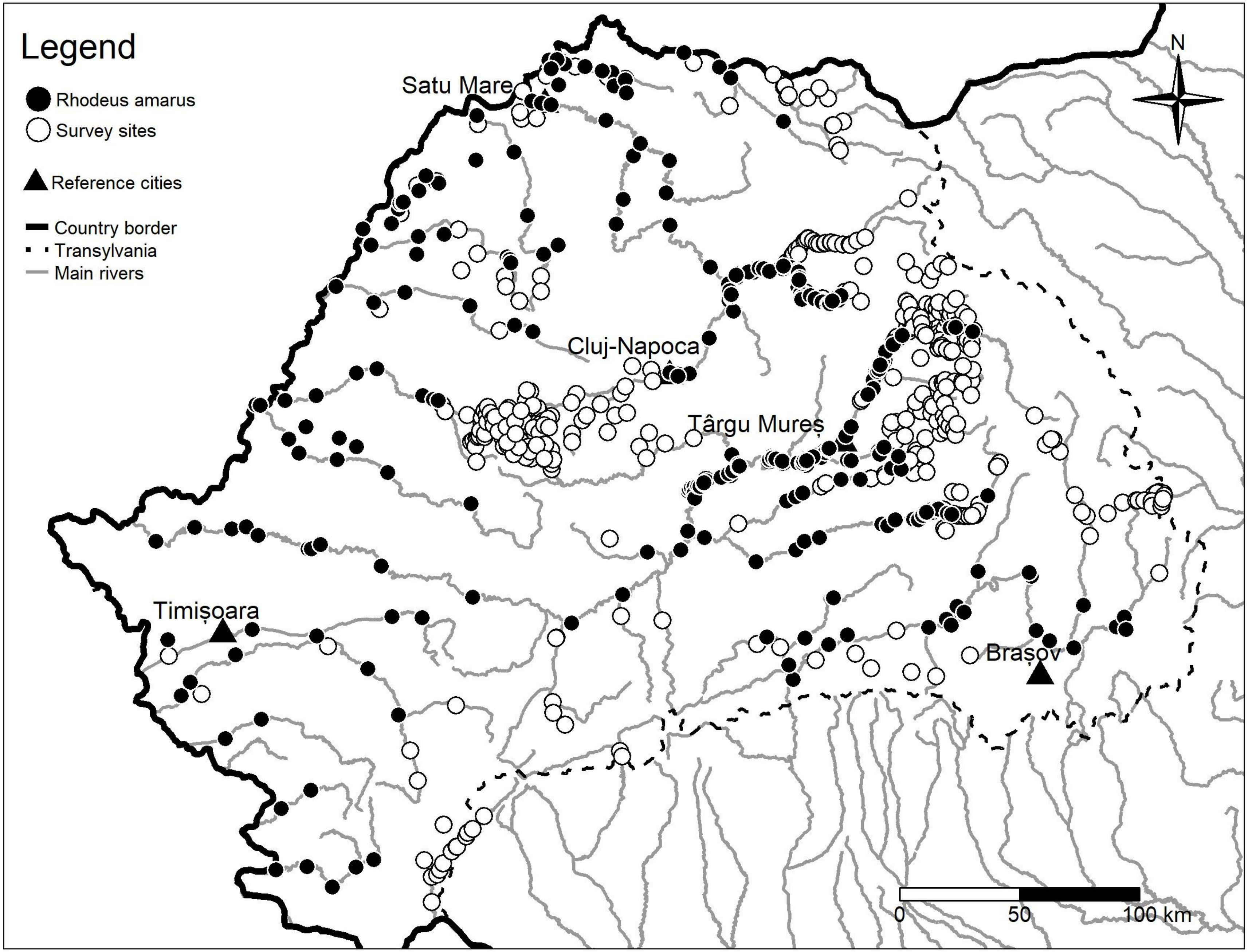

Map S56. Distribution of *Rhodeus amarus*

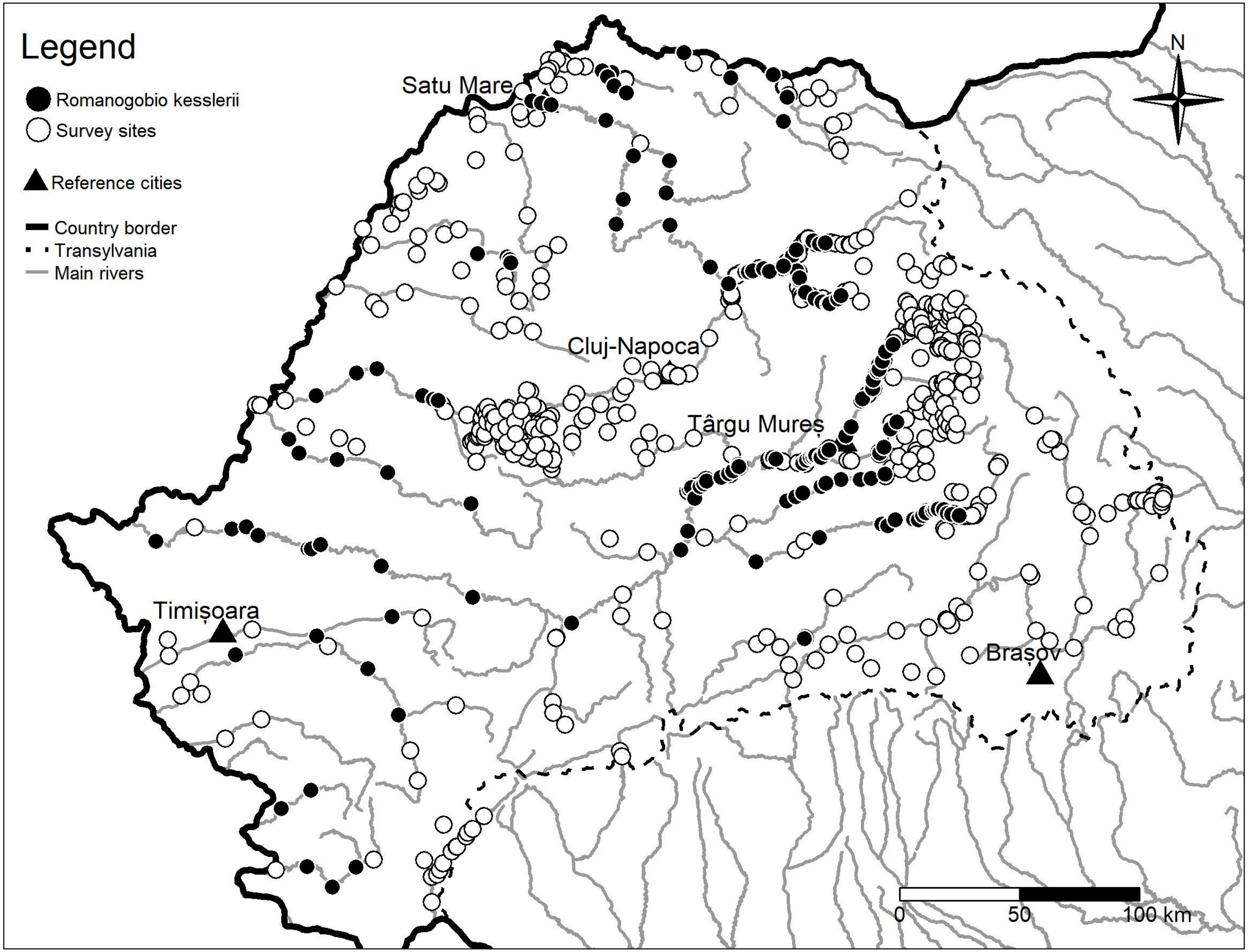

Map S57. Distribution of *Romanogobio kesslerii*

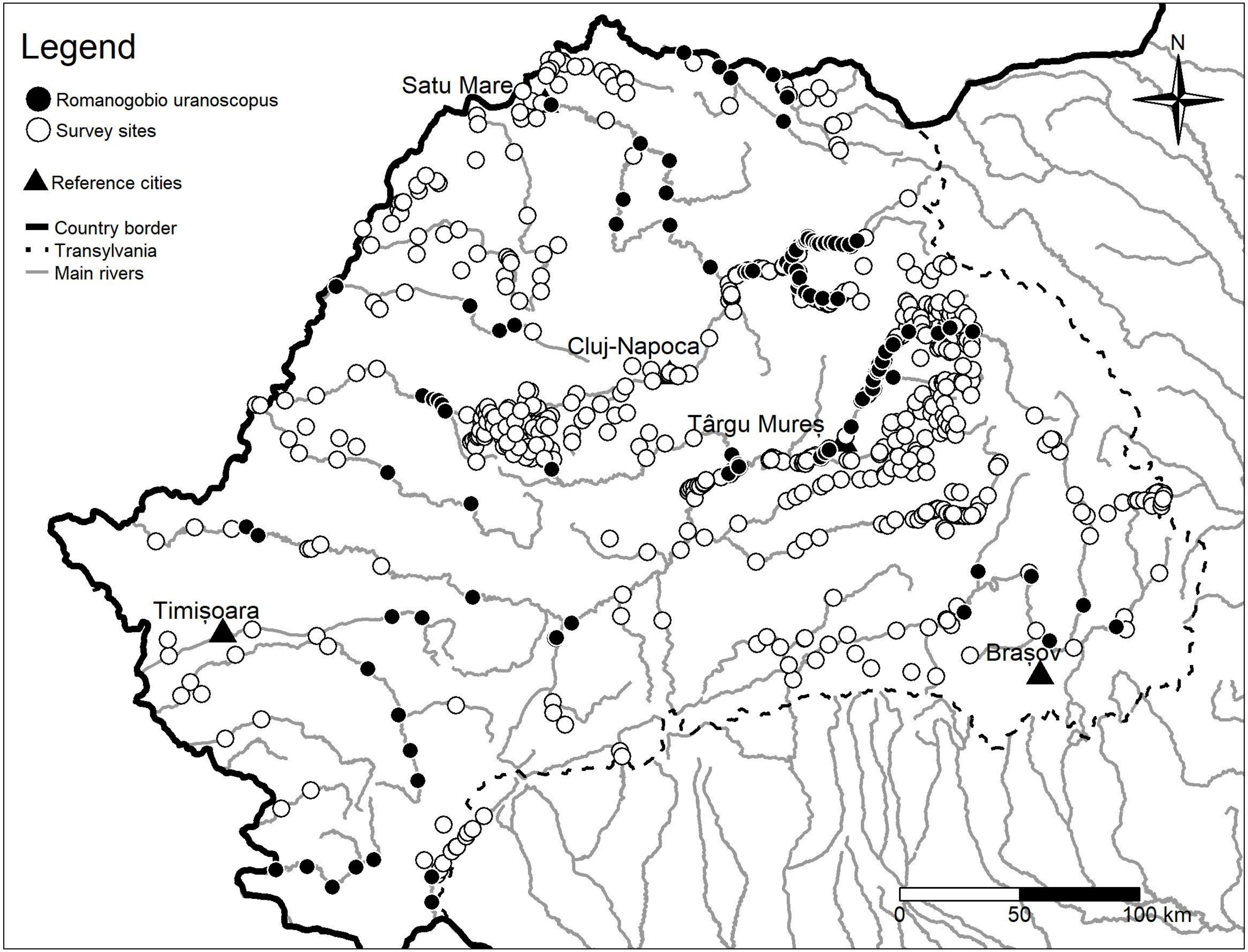

Map S58. Distribution of *Romanogobio uranoscopus*

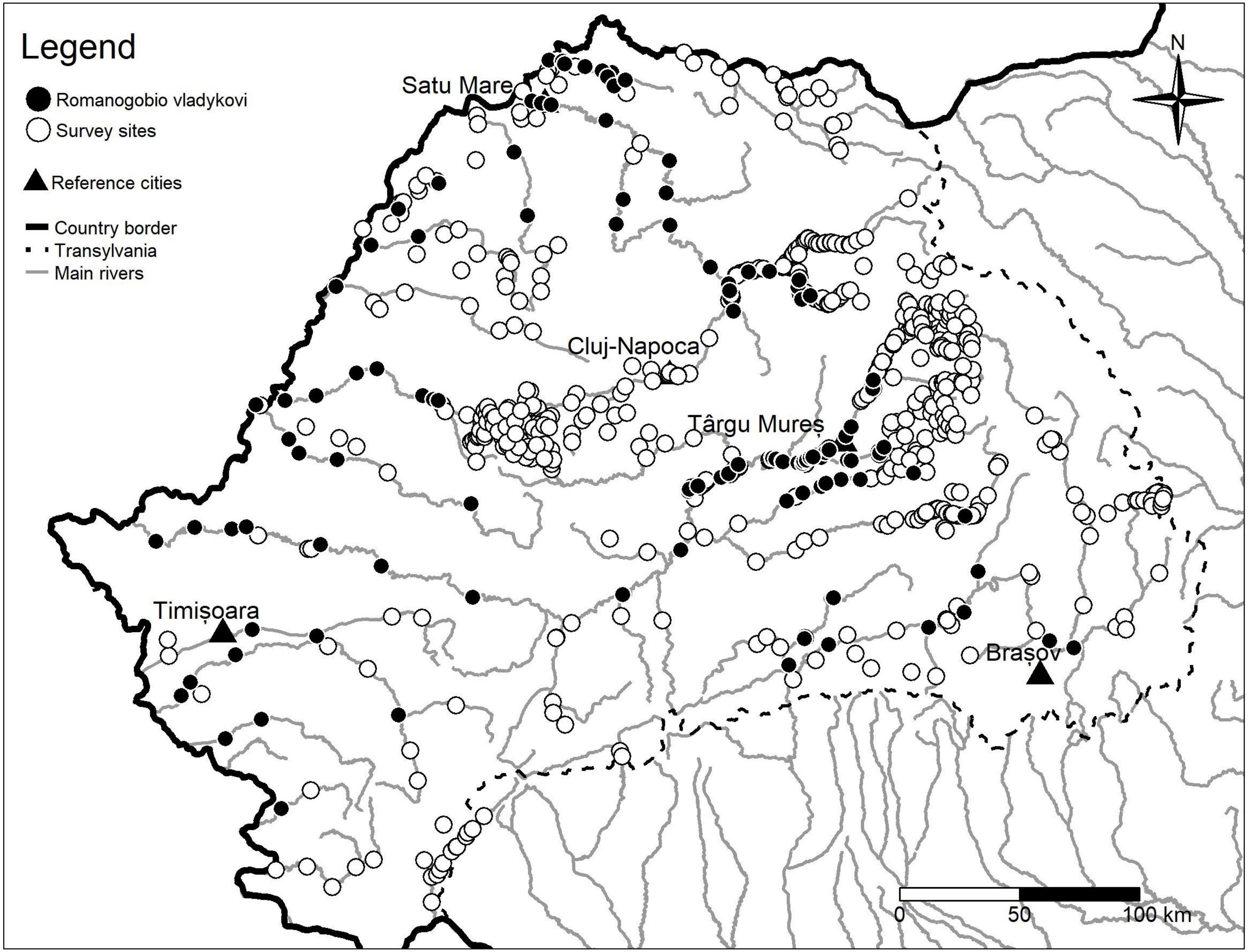

Map S59. Distribution of *Romanogobio vladykovi*

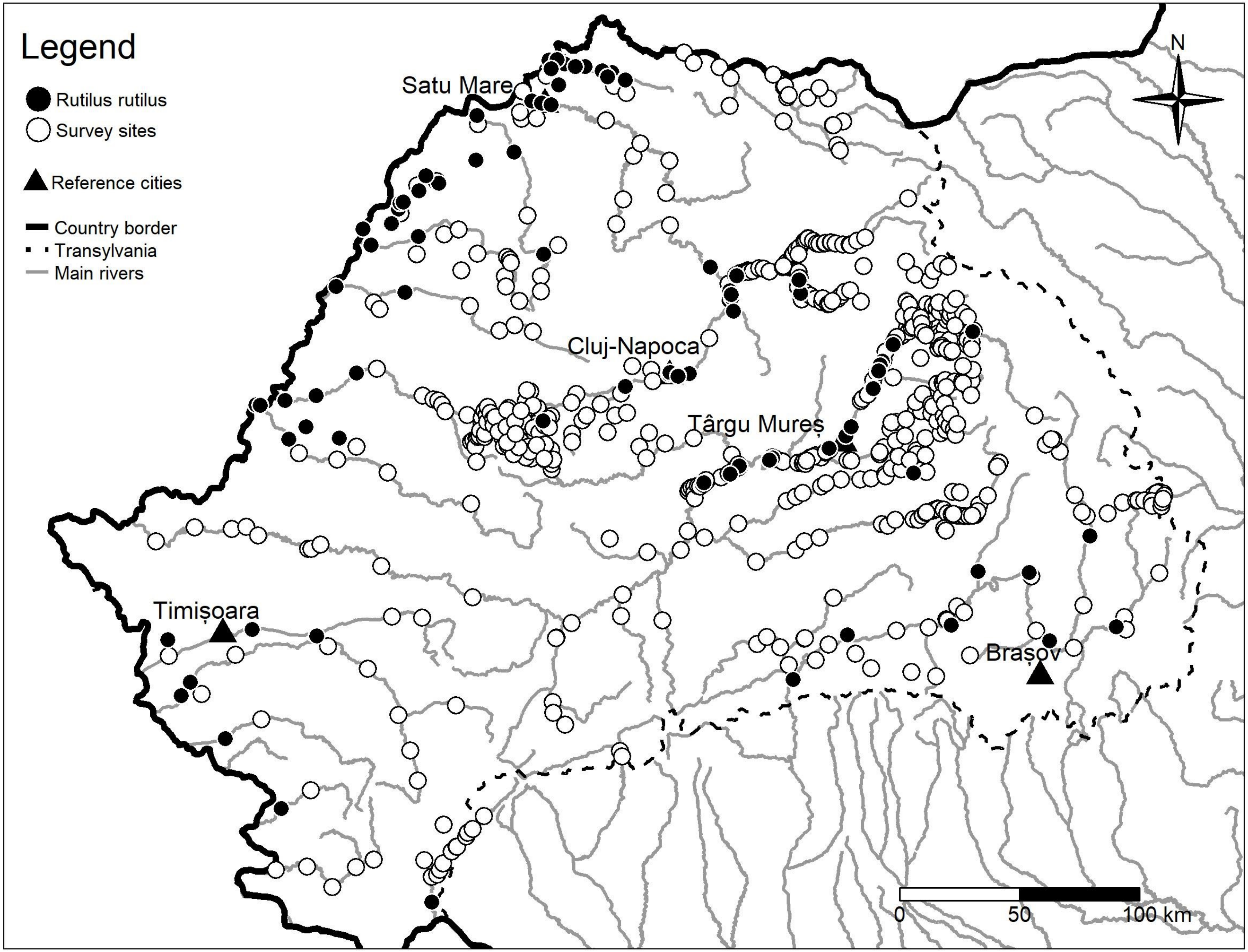

Map S60. Distribution of *Rutilus rutilus*

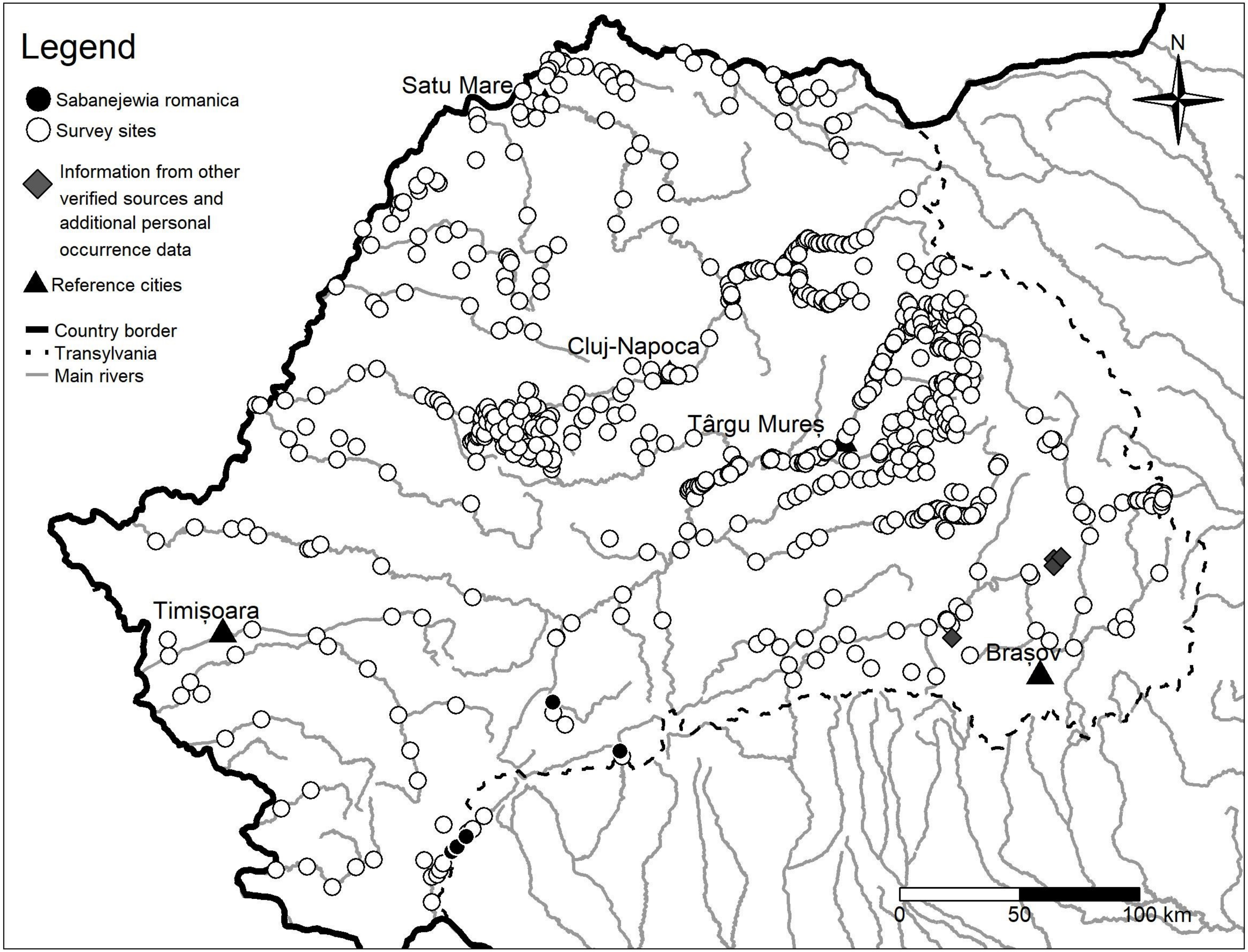

Map S61. Distribution of *Sabanejewia romanica*

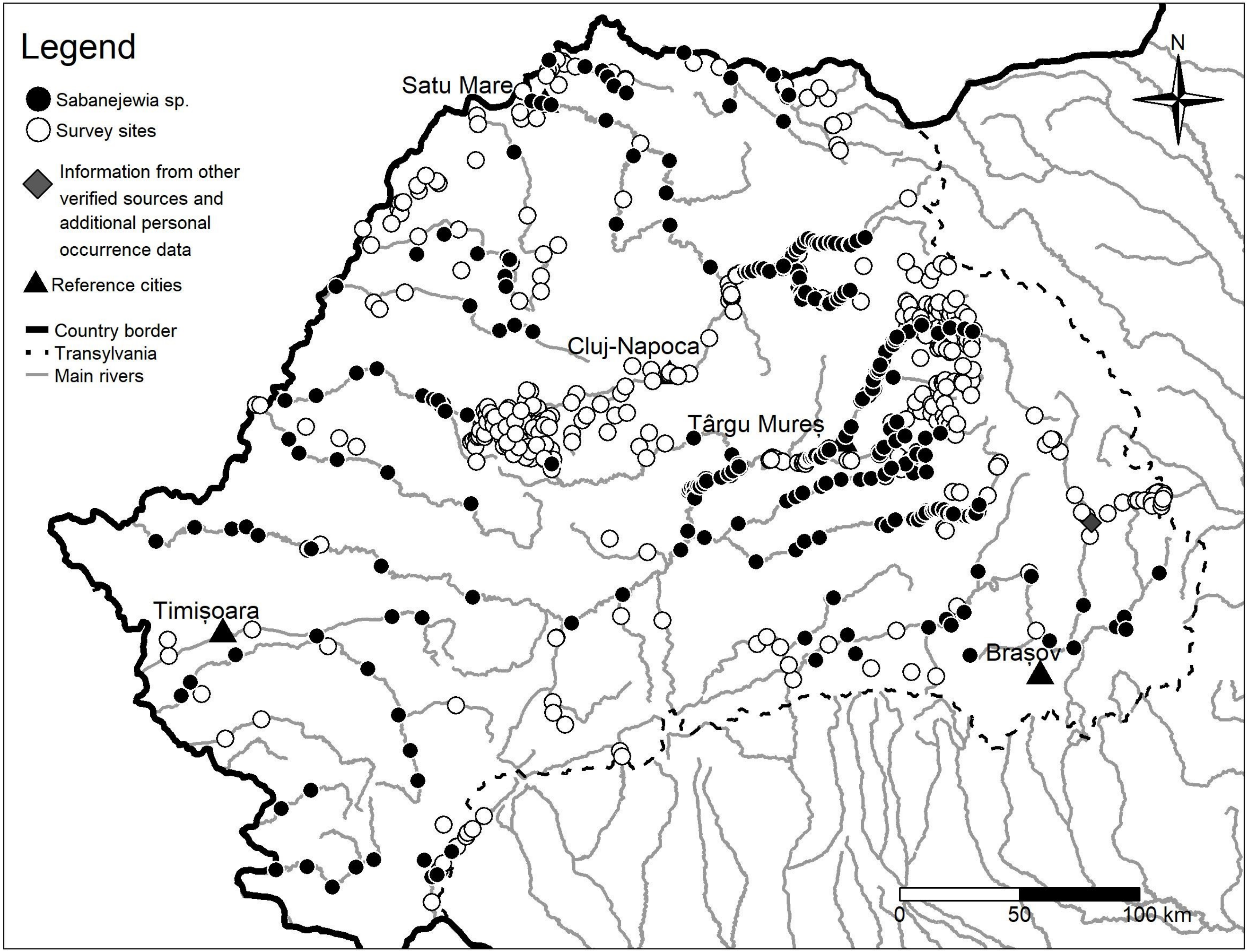

Map S62. Distribution of *Sabanejewia sp.*

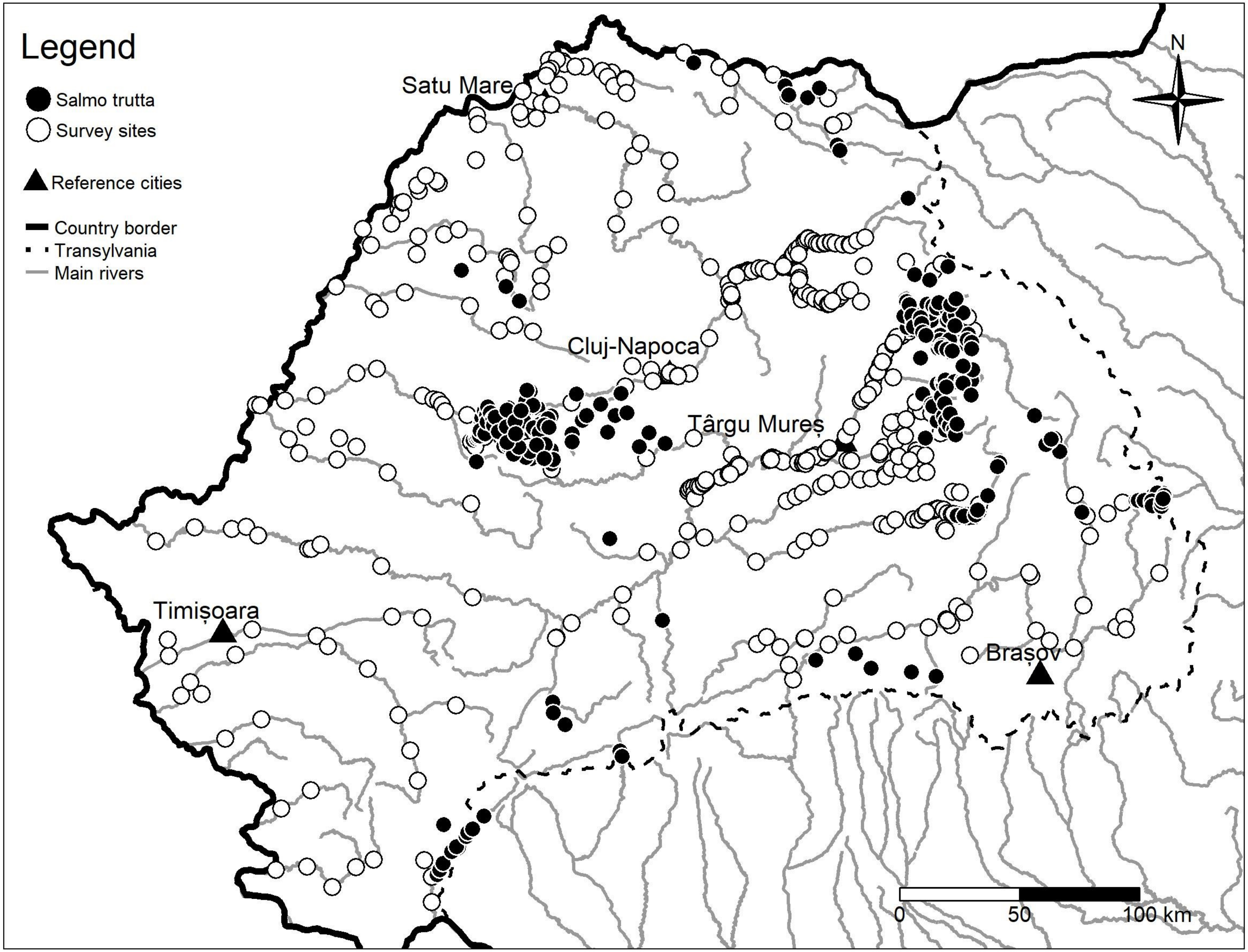

Map S63. Distribution of *Salmo trutta*

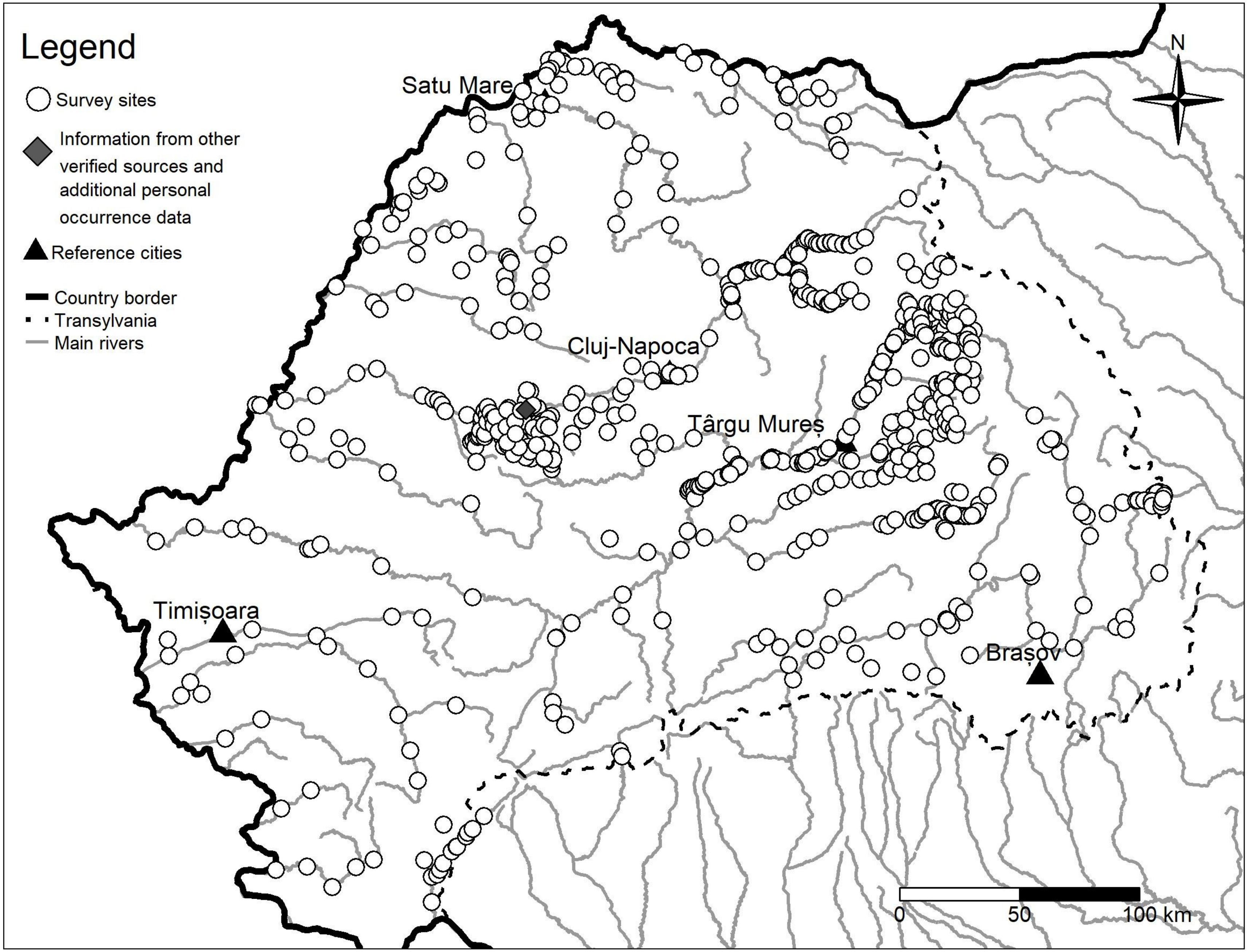

Map S64. Distribution of *Salvelinus alpinus*

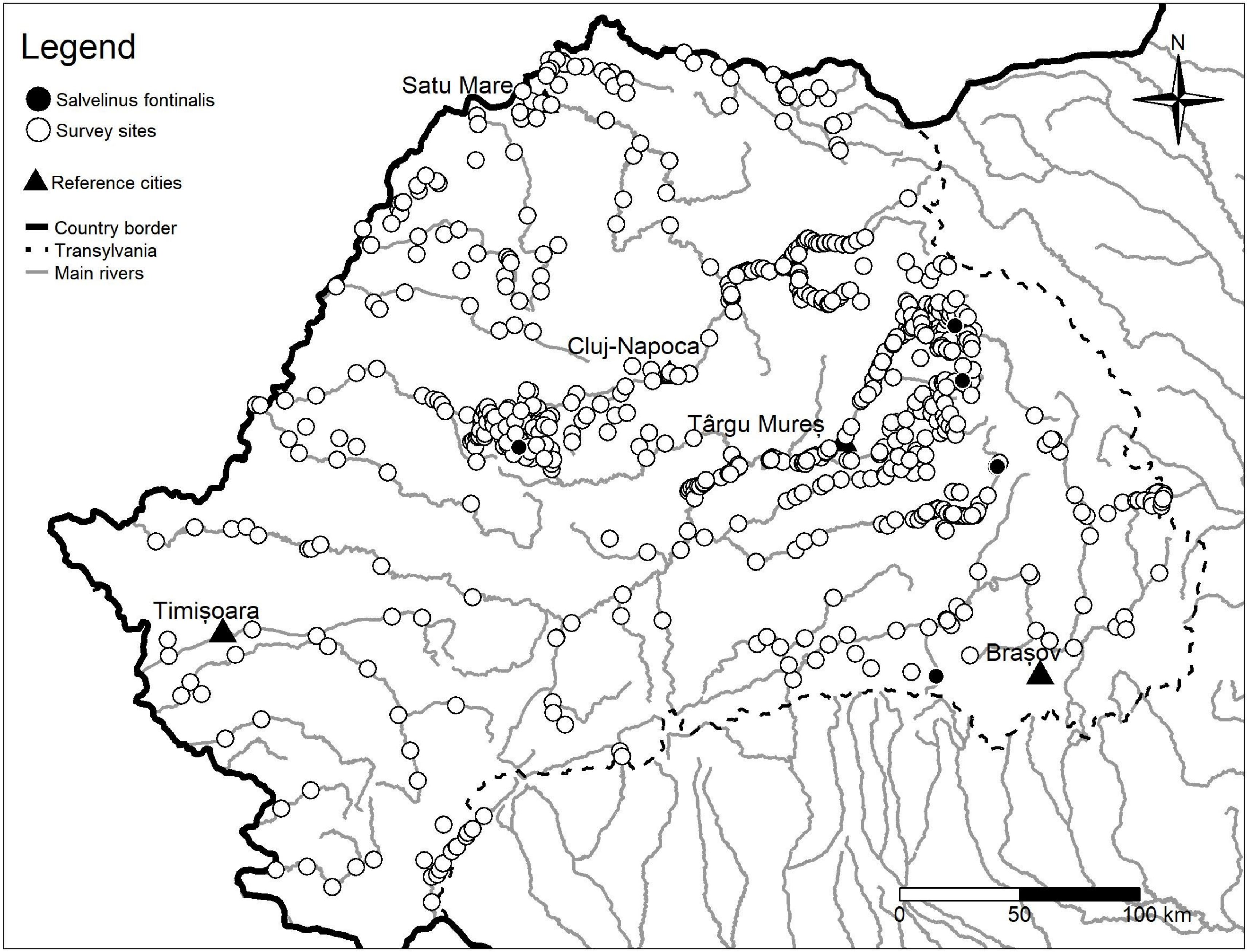

Map S65. Distribution of *Salvelinus fontinalis*

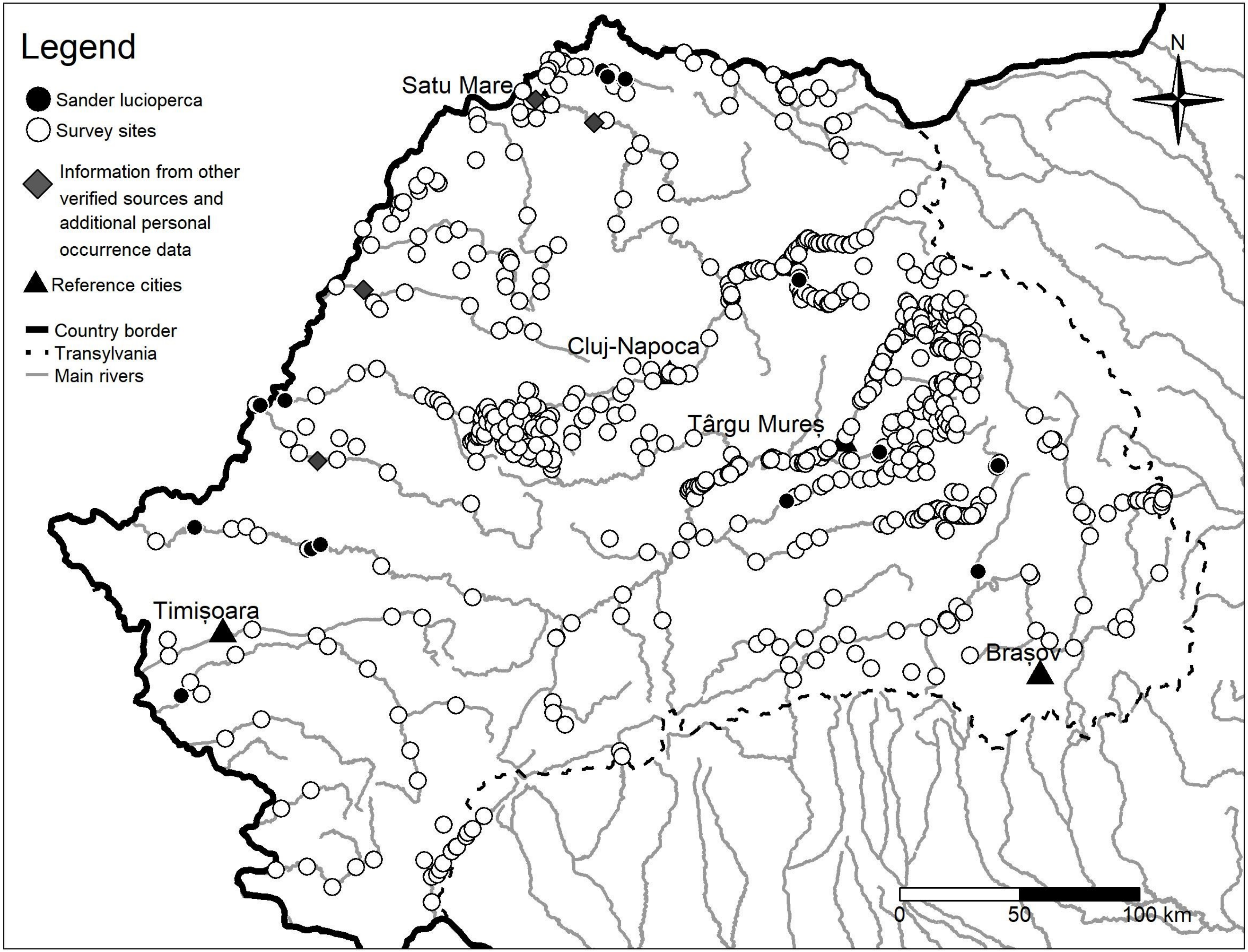

Map S66. Distribution of *Sander lucioperca*

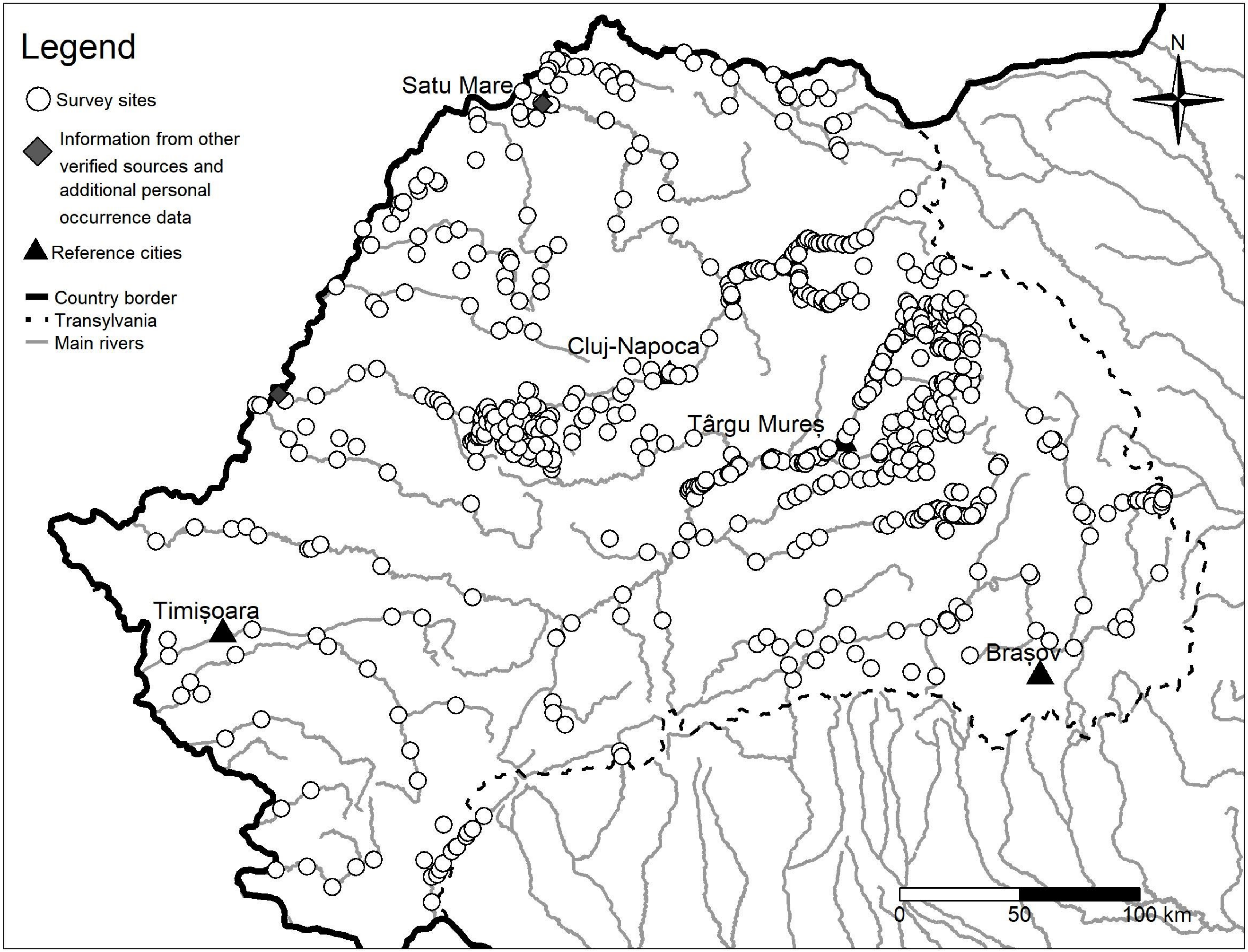

Map S67. Distribution of *Sander volgensis*

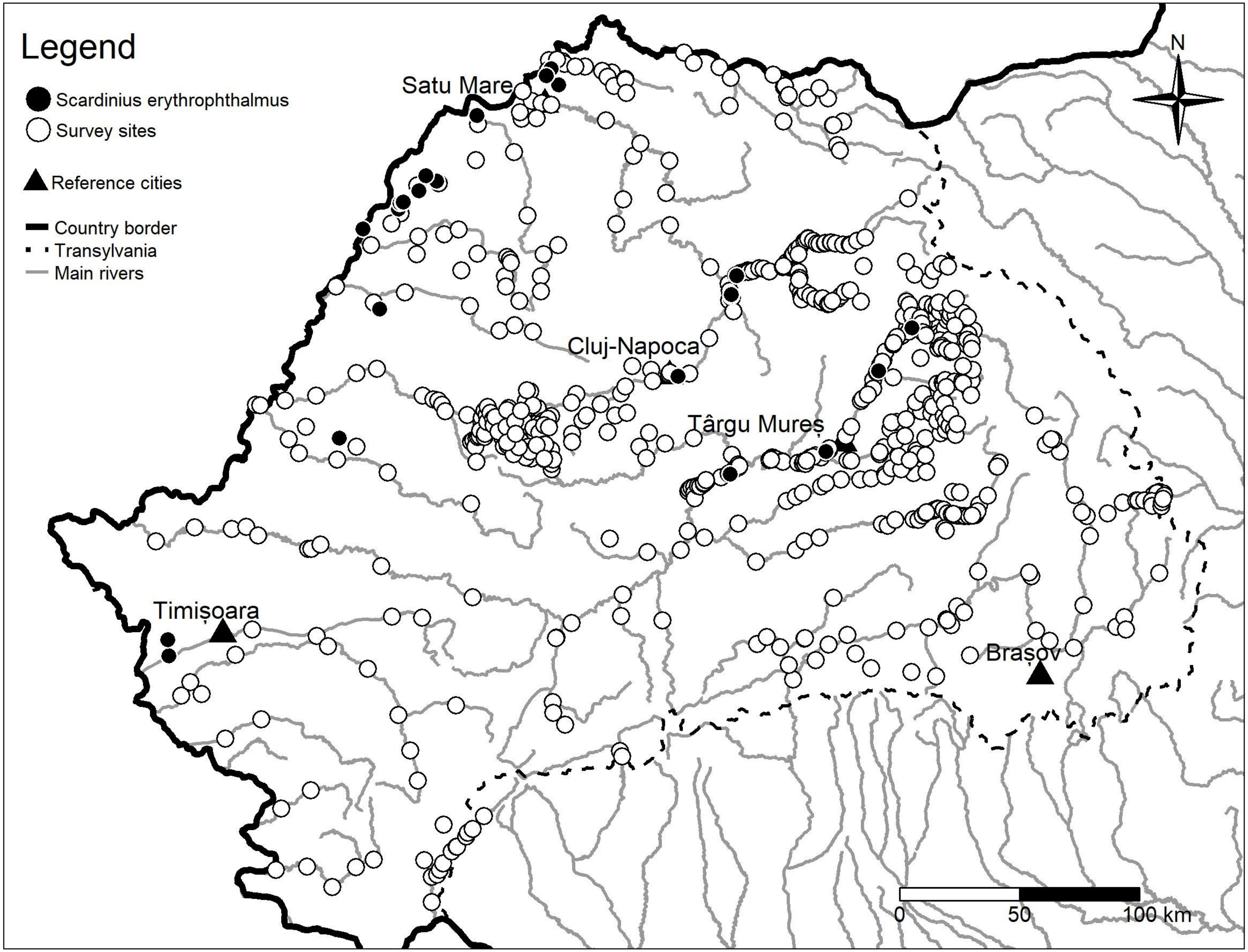

Map S68. Distribution of *Scardinius erythrophthalmus*

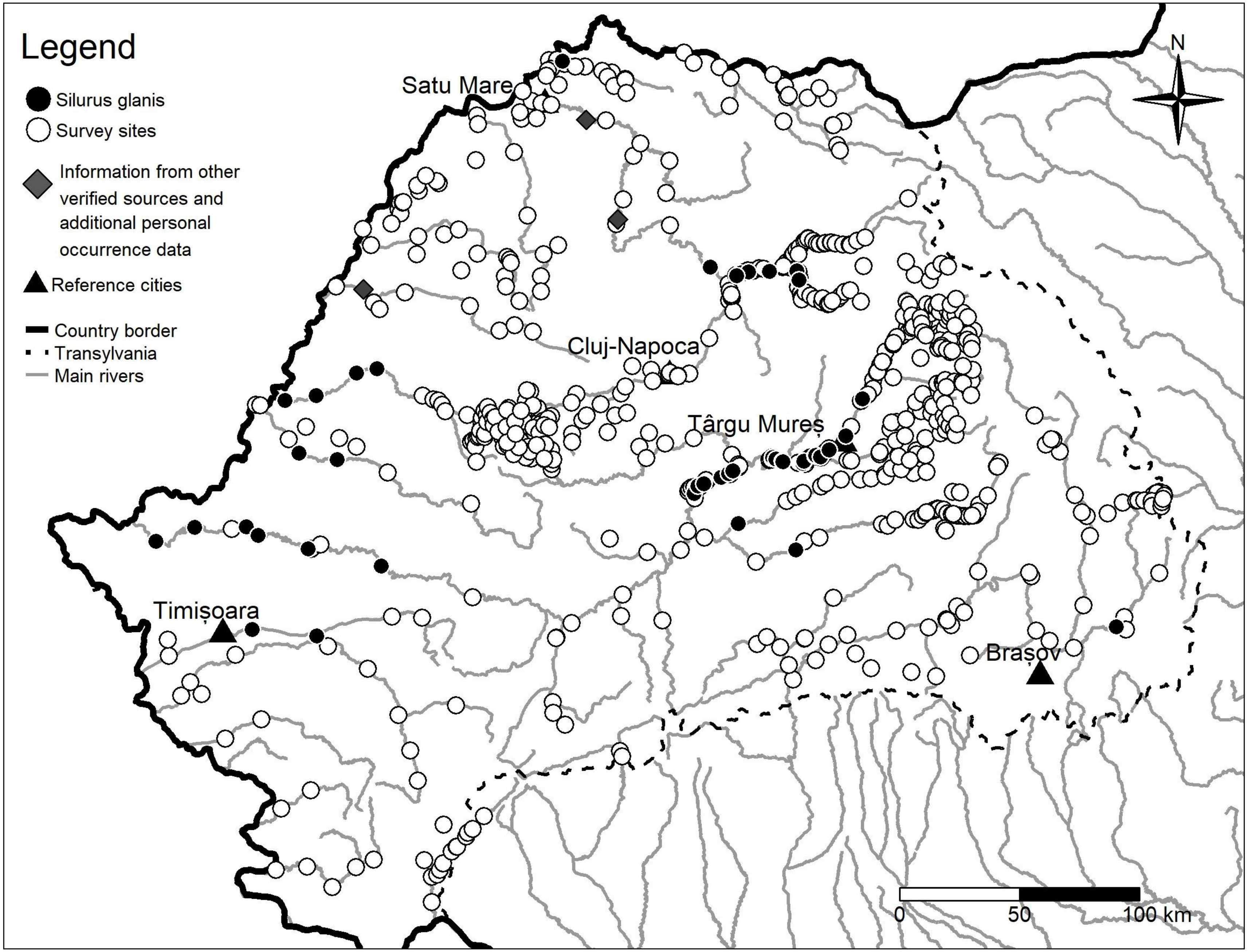

Map S69. Distribution of *Silurus glanis*

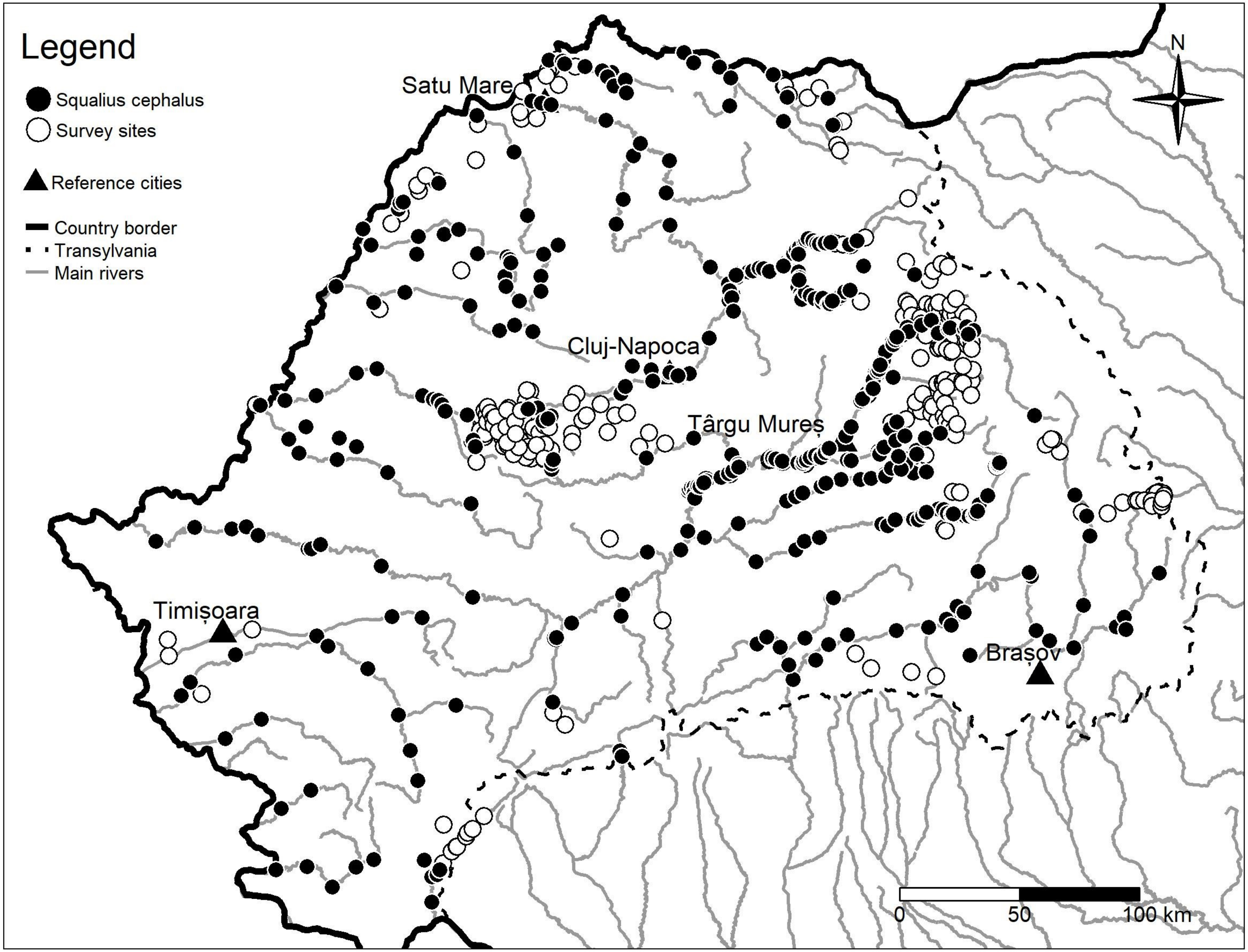

Map S70. Distribution of *Squalius cephalus*

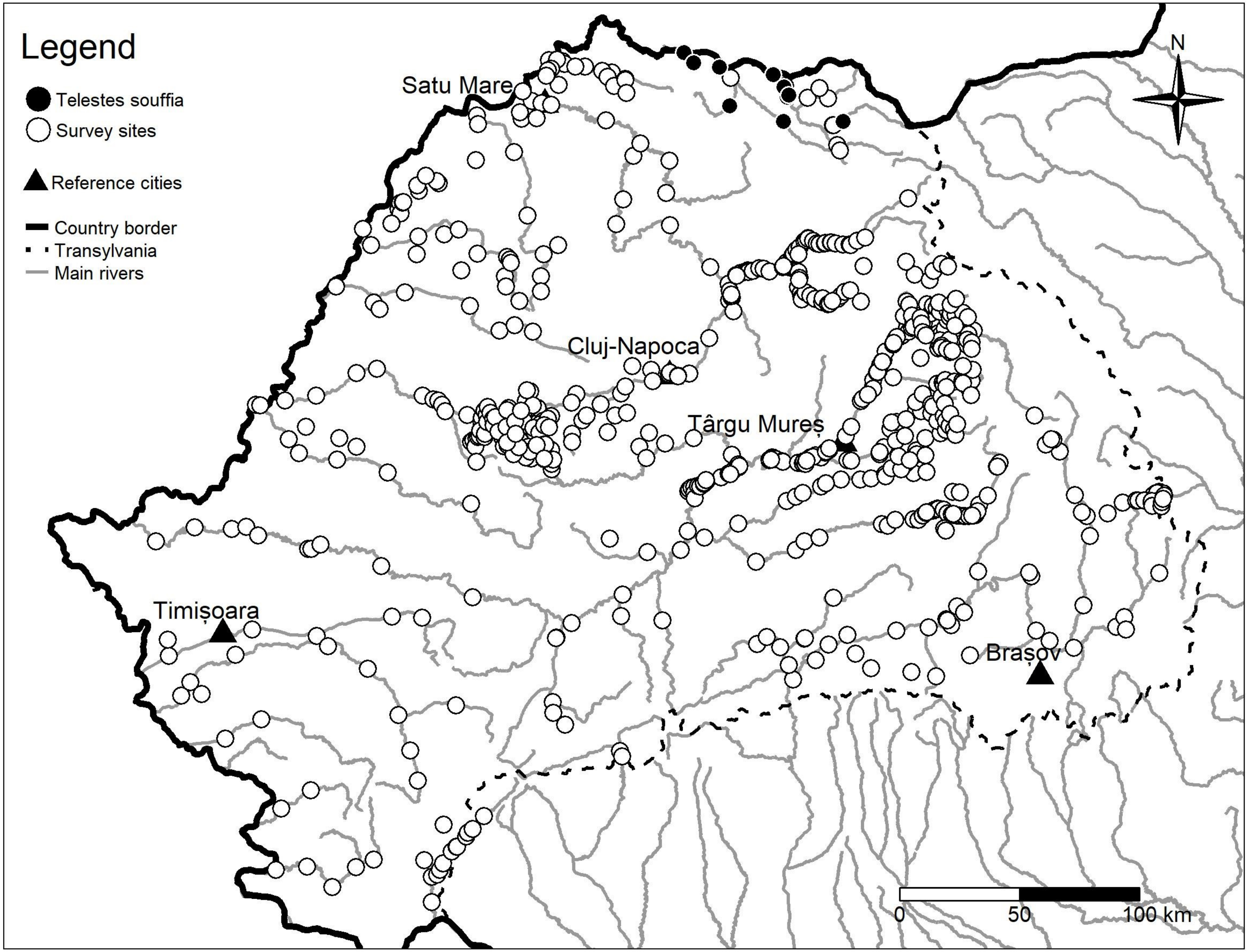

Map S71. Distribution of *Telestes souffia*

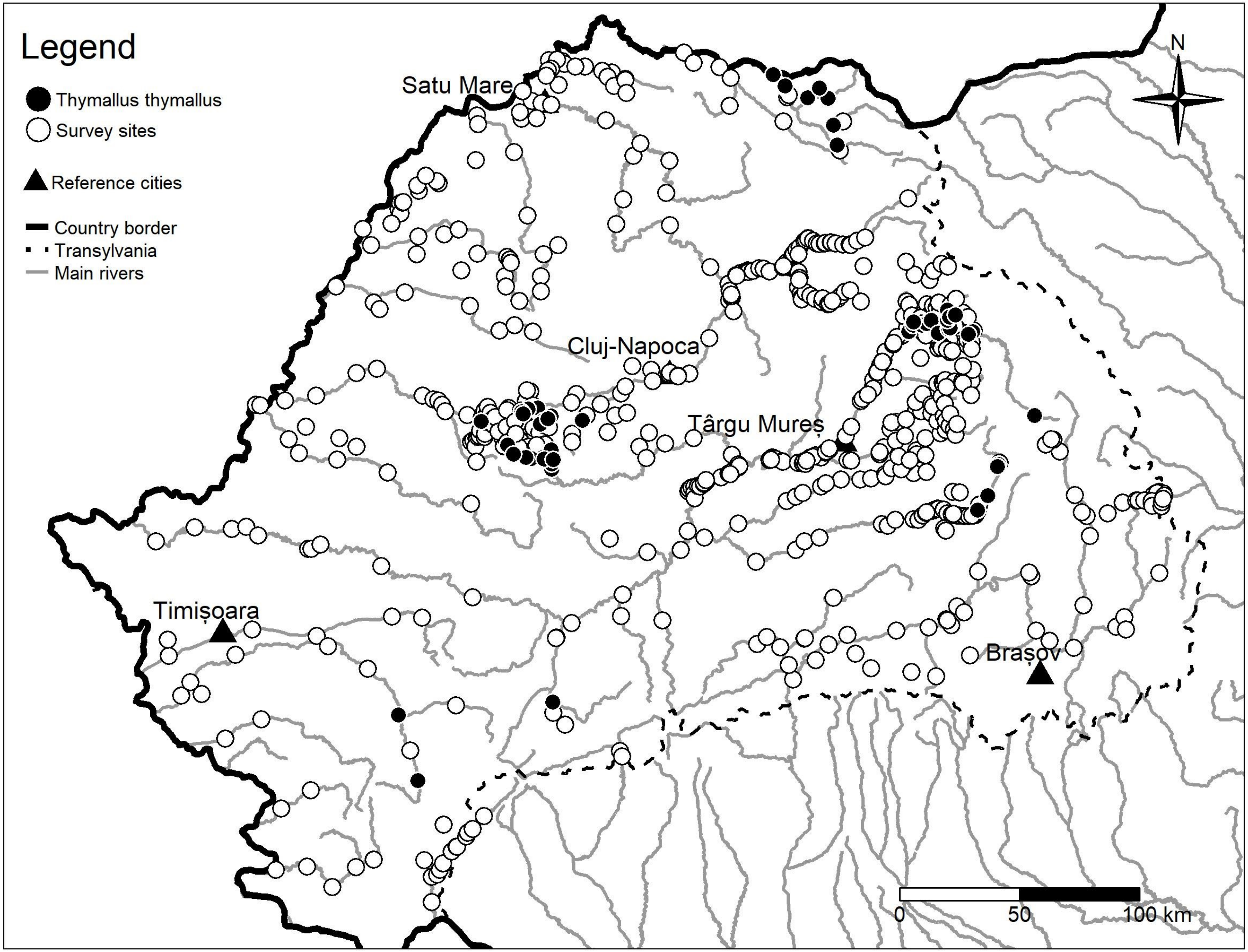

Map S72. Distribution of *Thymallus thymallus*

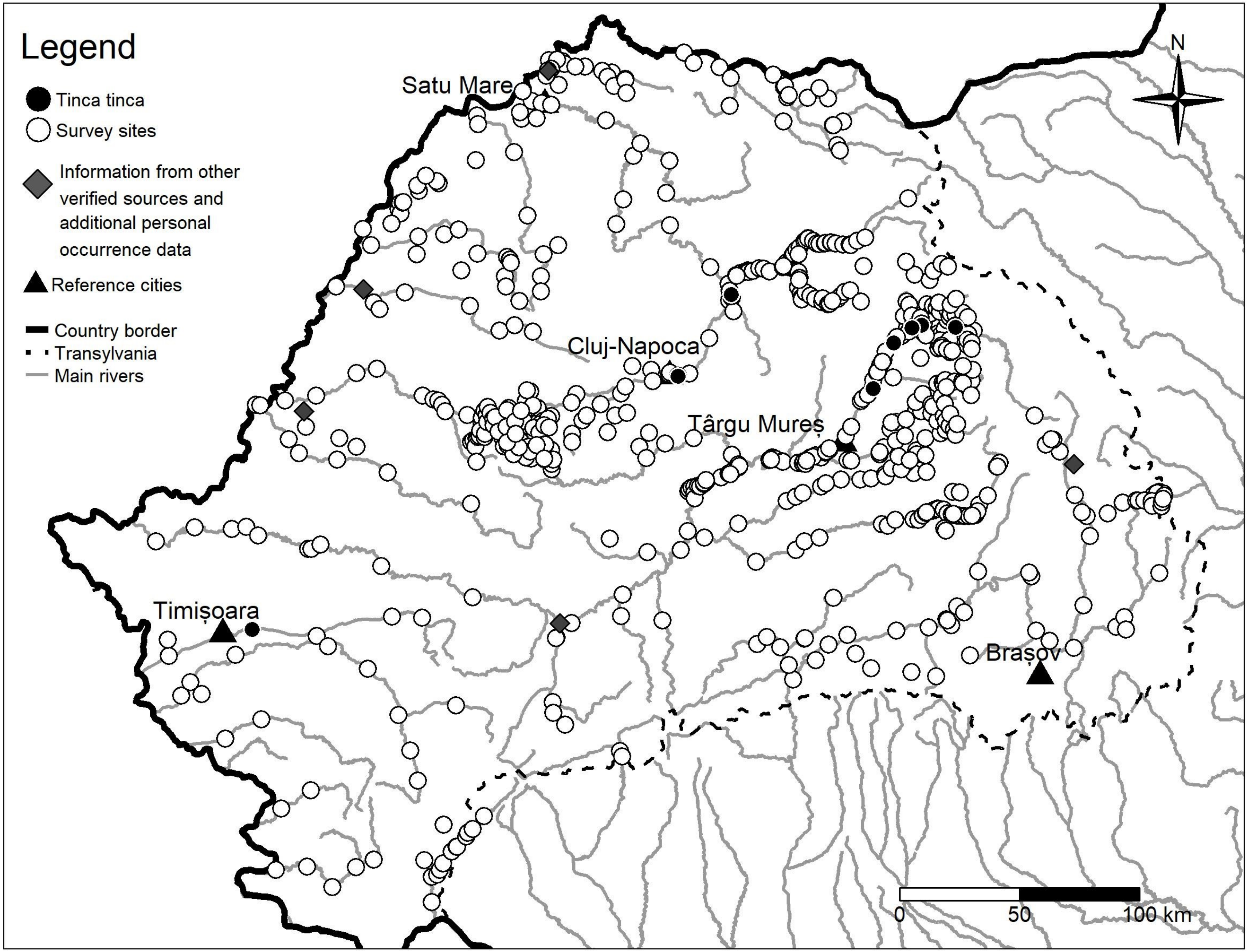

Map S73. Distribution of *Tinca tinca*

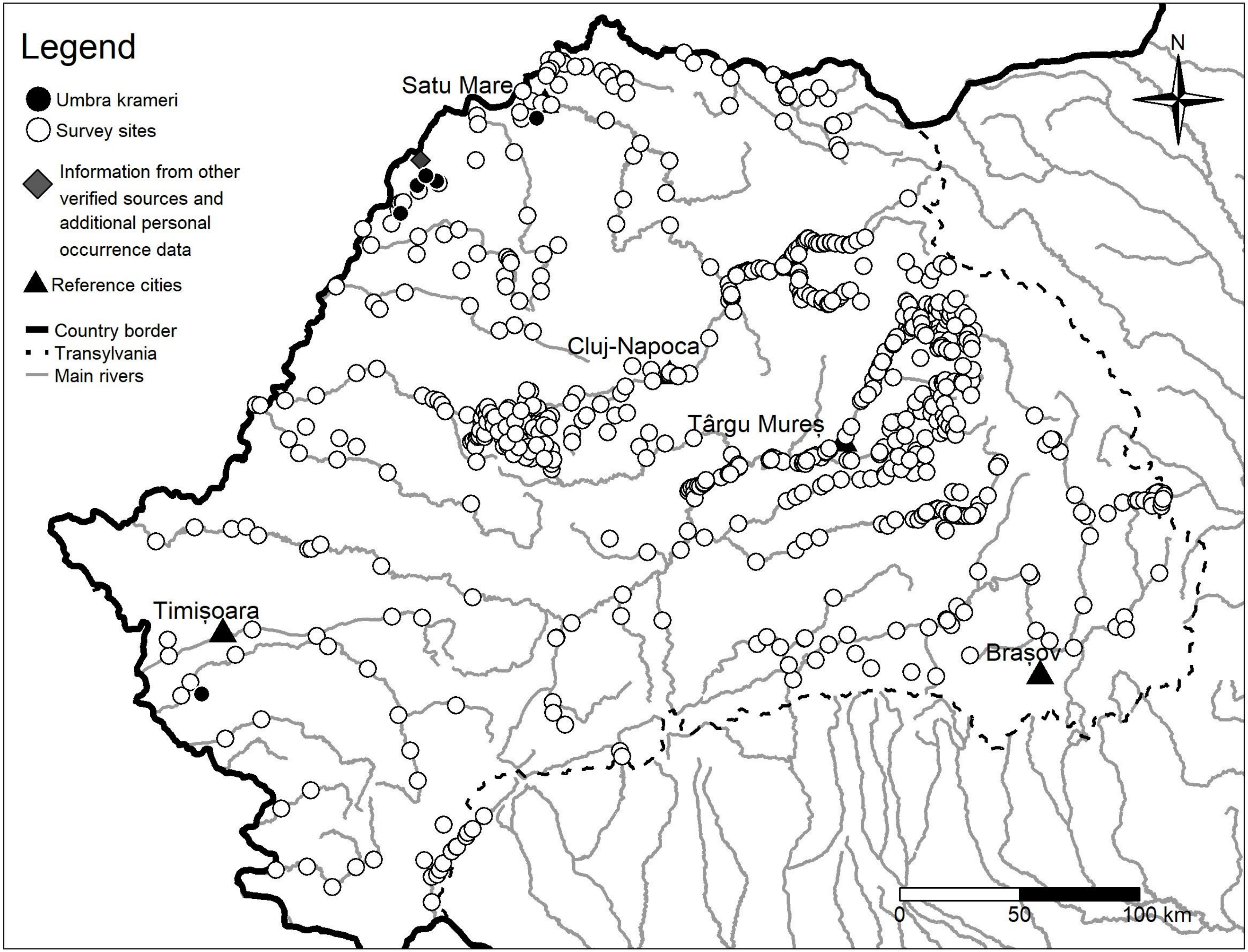

Map S74. Distribution of *Umbra krameri*

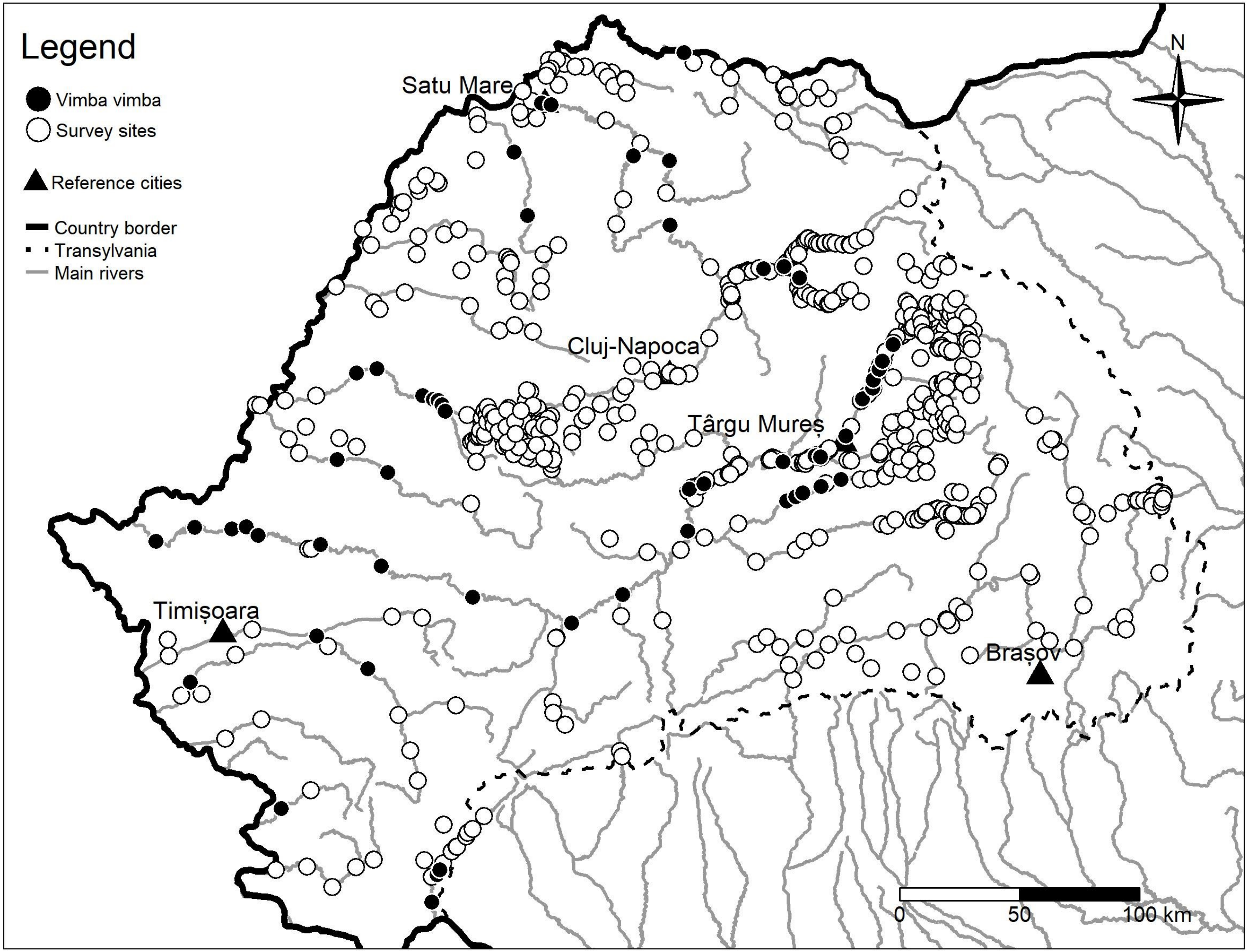

Map S75. Distribution of *Vimba vimba*

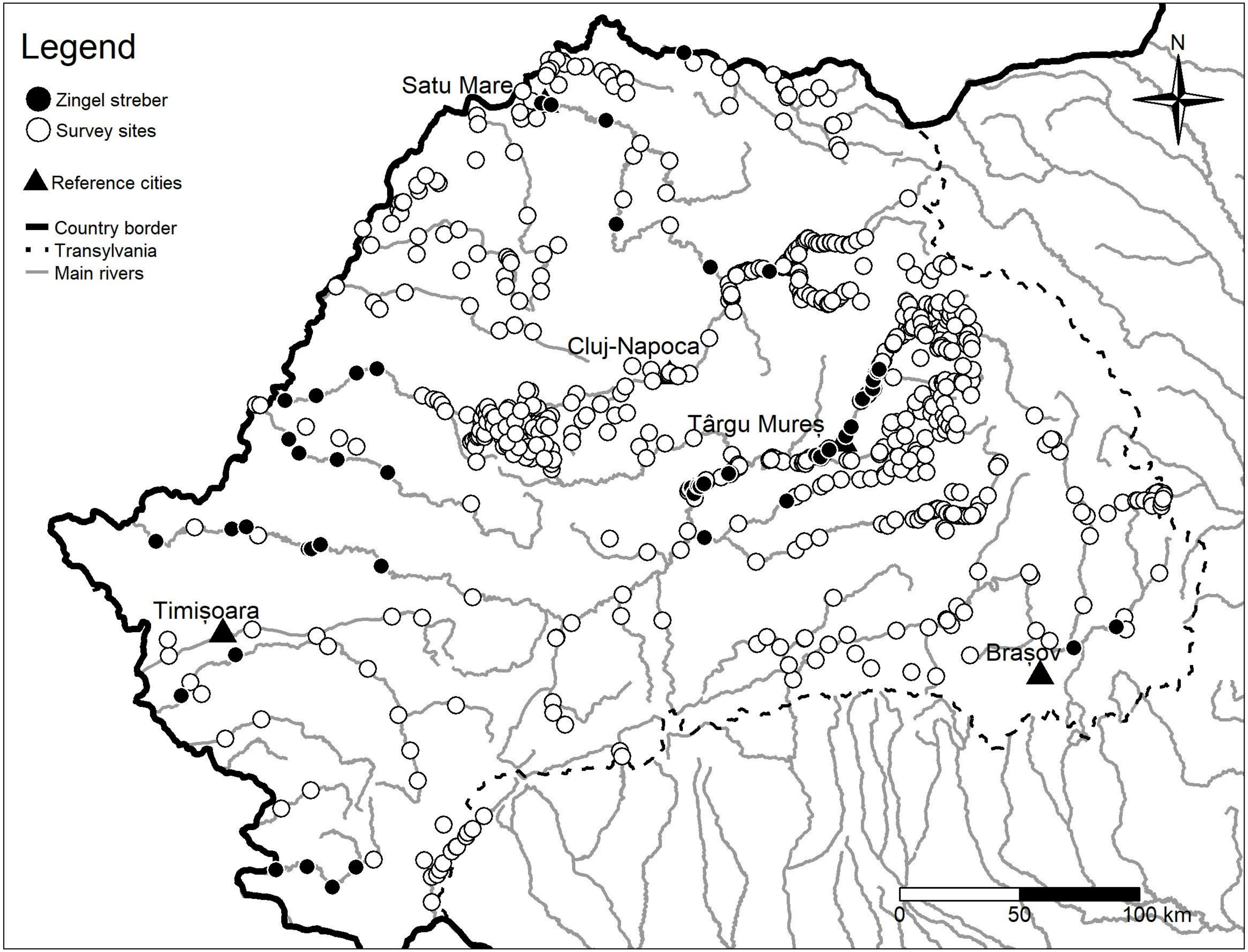

Map S76. Distribution of *Zingel streber*

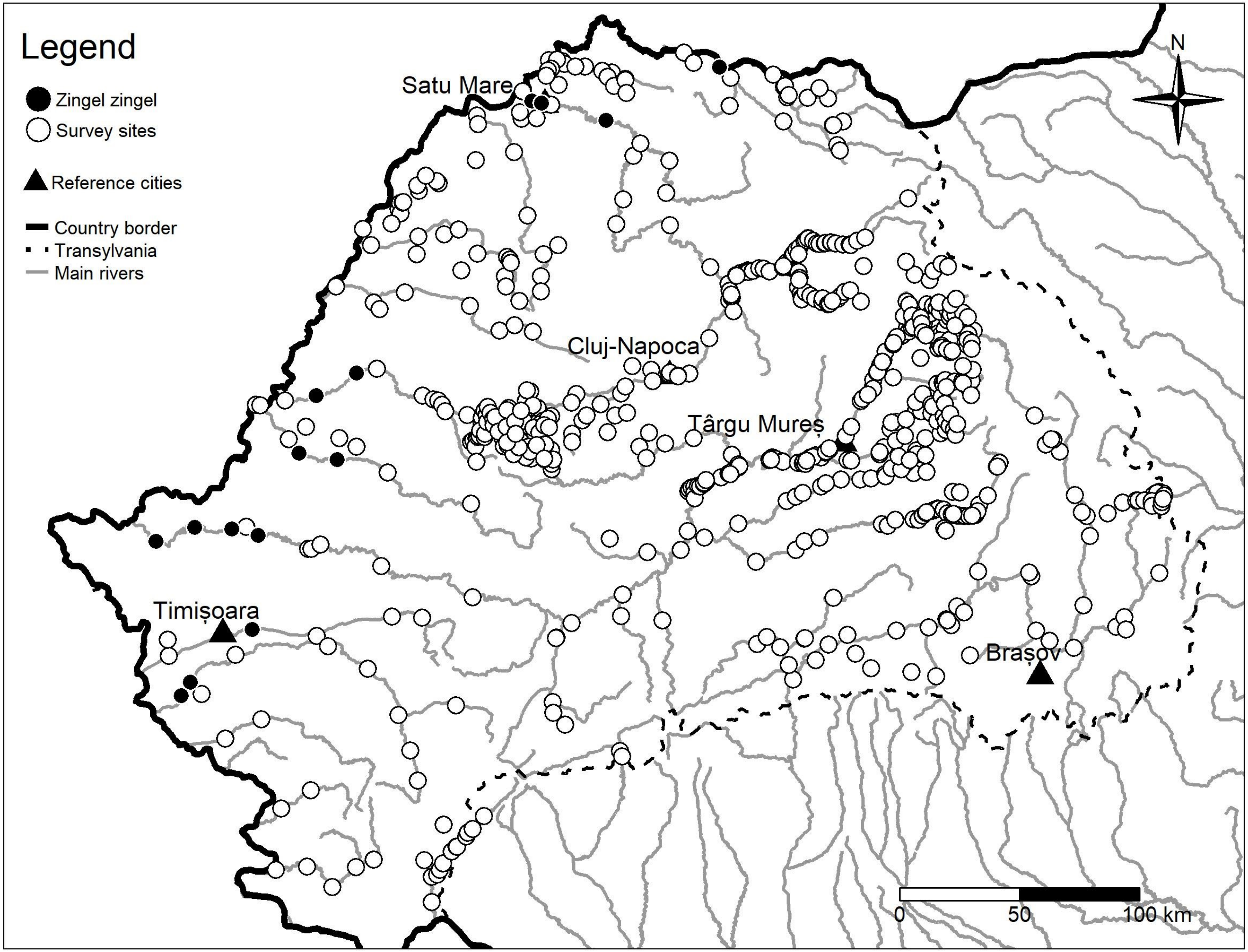

Map S77. Distribution of *Zingel zingel*
